# Autofluorescence as a noninvasive biomarker of senescence and advanced glycation end products in *Caenorhabditis elegans*

**DOI:** 10.1038/s41514-021-00061-y

**Published:** 2021-06-07

**Authors:** Tomomi Komura, Mikihiro Yamanaka, Kohji Nishimura, Keita Hara, Yoshikazu Nishikawa

**Affiliations:** 1 Faculty of Human Life and Environment, Nara Women’s University, Nara, Japan; 2 Graduate School of Human Life Science, Osaka City University, Osaka, Japan; 3 Department of Bioscience, School of Agriculture, Tokai University, Kumamoto, Japan; 4 Department of Molecular and Functional Genomics, Interdisciplinary Center for Science Research, Organization of Research, Shimane University, Shimane, Japan; 5 Faculty of Life and Environmental Science, Shimane University, Shimane, Japan; 6 Air Water Biodesign Inc., Hyogo, Japan; 7 Present Address: Faculty of Human Sciences, Tezukayamagakuin University, Osaka, Japan

**Keywords:** Caenorhabditis elegans, Biomarkers, Ageing

## Abstract

To assess the utility of autofluorescence as a noninvasive biomarker of senescence in *Caenorhabditis elegans*, we measured the autofluorescence of individual nematodes using spectrofluorometry. The fluorescence of each worm increased with age. Animals with lower fluorescence intensity exhibited longer life expectancy. When proteins extracted from worms were incubated with sugars, the fluorescence intensity and the concentration of advanced glycation end products (AGEs) increased over time. Ribose enhanced these changes not only in vitro but also in vivo. The glycation blocker rifampicin suppressed this rise in fluorescence. High-resolution mass spectrometry revealed that vitellogenins accumulated in old worms, and glycated vitellogenins emitted six-fold higher fluorescence than naive vitellogenins. The increase in fluorescence with ageing originates from glycated substances, and therefore could serve as a useful noninvasive biomarker of AGEs. *C. elegans* can serve as a new model to look for anti-AGE factors and to study the relationship between AGEs and senescence.

## Introduction

Mean lifespan in humans has been dramatically extended in developed countries during the last half century. However, progressive damage with aging causes detrimental effects on physiological functions, known as senescence, and senescence-related diseases also are increasing in proportion with longevity. Extension of the “healthspan” is now necessary rather than extending the lifespan alone. Interventions that slow senescence and extend the healthspan are desired.

Brenner et al. introduced *Caenorhabditis elegans*, which is a small, free-living soil nematode that feeds on bacteria, for use in molecular genetics studies of differentiation and development^1^. Subsequently, the worm has been used extensively as an experimental system for biological studies, including research on senescence, because of this animal’s simplicity, transparency, ease of cultivation, short lifespan, and suitability for genetic analysis^2^. In the worm, senescence can be examined by attenuation of locomotive ability, stress resistance, cognitive ability, and pharyngeal pumping, and by accumulation of the so-called age pigment lipofuscin^3–5^.

A variety of chemicals, including antioxidants and several drugs, are candidates to prevent senescence^6,7^. Furthermore, since we reported that the healthspan and lifespan of nematodes could be extended by feeding on probiotic bacteria^8,9^, the longevity effects of beneficial bacteria have also been demonstrated by a number of laboratories^10–12^. The longevity effects have been examined by survival curves, but such studies in worms require more than 3 weeks, despite the relatively short lifespan of *C. elegans*. To evaluate the effects of interventions against senescence, convenient and noninvasive indicators of senescence are desired to trace the influence in individual worms and (ultimately) in humans as the final target.

Lipofuscin, a so-called age pigment, has been extensively used as an indicator of senescence. However, the exact relationship among autofluorescence, senescence, and lifespan in the worm has remained unclear. Gerstbrein et al. reported that age pigments are valid reporters of nematode healthspan^13^, whereas Coburn et al. reported that blue fluorescence (excitation/emission wavelengths centered on 340/430 nm) serves as a death marker for several hours before and after death in *C. elegans*^14^. It has been suggested that the increases in blue autofluorescence over time observed in populations of aging *C. elegans* might reflect not the aging rate or health state of the population per se, but instead the fraction of dead or almost-dead individuals in the sample^15^.

Advanced glycation end products (AGEs) have attracted scientific attention^16^ because they are formed in high amounts in diabetes cases, but also during physiological aging, and lead to oxidative stress. AGEs are complex compounds formed by non-enzymatic reactions between reducing sugars and amino groups in proteins, lipids, or nucleic acids; Schiff bases and Amadori products then are formed through Maillard reactions, yielding AGEs. AGE content is high in patients with diabetes, rheumatoid arthritis, Alzheimer’s disease, and other diseases^17–19^. In patients with diabetes, AGE accumulation correlates well with the severity of the microvascular disease and rises with age. However, whether AGEs represent a cause of pathogenesis or are only secondary products remains to be elucidated. AGEs are also considered as possible causes of senescence^20,21^, as suggested from findings in a *C. elegans* model of sugar toxicity^22,23^.

A systematic enzyme-linked immunosorbent assay (ELISA) method has been developed by employing antibodies with specificity for several AGE compounds^24–26^. However, noninvasive methods to permit rough estimations of AGE levels are also required. Since several AGEs are reportedly fluorescent and their associated fluorescence intensity shows significant correlation with the levels measured by ELISA^27^, we examined whether this fluorescence could be employed as an alternative marker for tracing the status of AGEs and senescence in a *C. elegans* model. To verify whether the observed fluorescence merely reflects the death fluorescence reported by Coburn et al., worms were examined by tracing the fluorescence of individual worms while timing the demise of the respective animals. Furthermore, the effects of inhibitors of AGE formation on fluorescence and AGE accumulation were also investigated to validate the utility of *C. elegans* as a model animal for AGE research.

## Results

### Autofluorescence of extracted proteins as a biomarker of aging and AGEs in worms

**Fig. 1:**
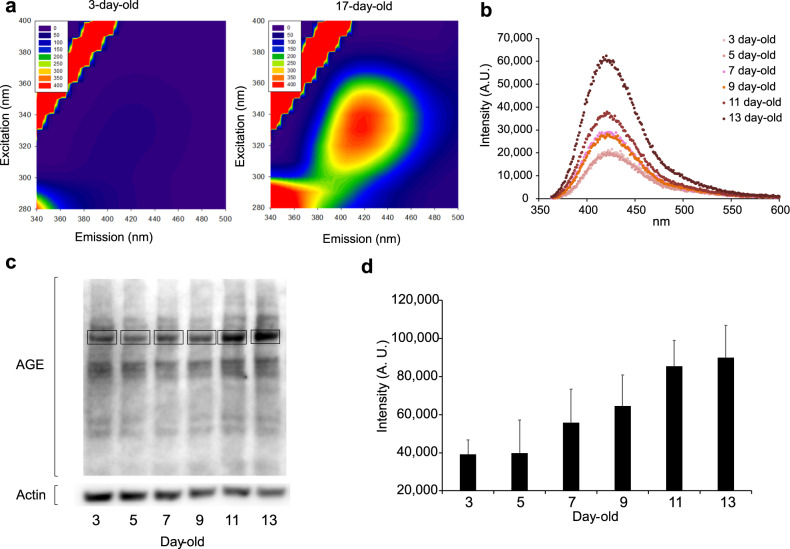
Fluorescence spectrophotometry and the AGEs of worm protein samples. **a** Excitation emission matrix (EEM) plots of autofluorescence from lysed worms at 3-day old and 17-day olds. The fluorescence levels at the emission wavelength in the EEM plots are displayed as colored heatmaps. One hundred worms (wild-type N2) each of 3-day-old and 17-day-old were lysed in 3 µL of lysis buffer and 4 µL of 15% SDS. Proteins were released by freeze-thaw cycles, and the photoluminescent spectra were collected with a fluorescence spectrophotometer (Hitachi FL-4500). Fluorescence properties of lysed nematodes were analyzed using EEM fluorescence spectroscopy. Excitation at 325–365 nm made 17-day-old worms fluoresce with the highest emissions in the 400–430 nm interval corresponding trajectories as a series of fluorescence intensities, whereas 3-day-old young worms did not fluoresce. Multivariate analysis (single-wavelength excitation with multiple-wavelength emission, and synchronous-scanning fluorometry) yielded EEM plots consisting of single-scan excitation. **b** Levels of autofluorescence (ex 340/em 360–600) by worm protein samples extracted from animals of each age group (3–13 days old). Worms (wild-type N2) were collected in tubes, washed five times, and lysed in each tube. Samples were then ground using a Mini Cordless Grinder (Funakoshi, Tokyo, Japan) to release protein. Protein contents were quantitatively measured as described in the Methods. A fluorescence spectrum was determined for each 30-µL sample (containing 1.5 μg of total protein) using a multimode grating microplate reader model SH-9000Lab (Corona Electric, Ibaraki, Japan). Each measurement was carried out three times. **c** Western blotting analysis of the AGE CML in samples extracted from worm (wild-type N2) populations of different ages. A representative photograph of three reproducible experiments is shown. Blots derived from different parts of the same gel (shown in Supplementary Fig. 1) and were processed in parallel. **d** Quantification of AGEs in western blots using densitometry. The bands surrounded by rectangular frames in Fig. 1c were quantified, and data from three reproducible experiments is shown as mean ± SE.

Fluorescence spectrophotometry of extracted proteins indicated that excitation at 325–365 nm made 17-day-old worms fluoresce with the highest emission in the 400–430 nm (peak 420 nm) interval (Fig. 1a). The blue fluorescence was comparatively lower during young adulthood (3-day-old to 5-day-old) but rose over time (in 7-day-old animals) (Fig. 1b). Lipofuscin, a so-called age pigment, emits red fluorescence (excitation at 530–560 nm, emission at 585–645 nm)^15^; in our previous study, this marker was detected in late-life (more than 13-day-old) worms^9^. Since the autofluorescence found in the present study appeared at an earlier stage and increased over time, blue autofluorescence was expected to be a better biomarker for tracing senescence.

Since several AGE compounds are known to be autofluorescent^27^, we examined the relationship between AGE contents and blue fluorescence by western blotting in which an antibody to Nε-(carboxymethyl) lysine (CML), a representative AGE, was used. The blotting showed that an outstanding band was amplified over time (Fig. 1c and Supplementary Fig. 1). Indeed, the quantification of the region indicated increased AGE (Fig. 1d), although CML is not autofluorescent.

**Fig. 2:**
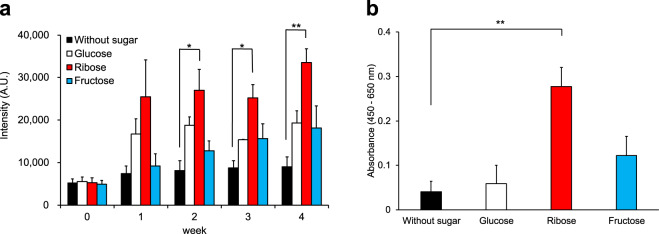
Blue fluorescence and AGEs of worms’ extracted proteins after in vitro glycation. **a** Autofluorescence (ex 340/em 430) from worm proteins (wild-type N2) after incubation with sugars for four weeks in vitro. Two-factor factorial ANOVA was applied with Scheffe’s *F* test. **b** AGE levels on the fourth week were also measured by ELISA with anti-CML antibody and expressed as the absorbance. Data are shown as mean ± SE. Single-factor ANOVA was applied with Dunnett’s test; * and ** indicate statistically significant differences at *p* values < 0.05 and < 0.01, respectively.

To determine if the autofluorescence indirectly indicated an increase in AGEs formed by the Maillard reaction, proteins extracted from worm homogenates were aseptically incubated for up to 4 weeks with reducing sugars such as glucose, ribose, and fructose. The fluorescence of the proteins rose over time in the presence of sugars, while there was no marked increase in fluorescence in reactions performed without sugars (Fig. 2a). Worm proteins became fluorescent via glycation even in vitro. Among the reactions performed with each of the three sugars, those with ribose produced the highest fluorescence, followed by the two other sugars. This finding could be explained by a report by Bunn and Higgins in which the reactivity of each monosaccharide with amino groups to form Schiff base linkages is dependent on the extent to which it exists in the open (carbonyl) structure rather than in a ring (hemiacetal or hemiketal) structure, and the rates of reactivity increased in the order of ribose (10.0), fructose (4.5), and glucose (0.6) (×10^−3^ mM^−1^ h^−1^), respectively^28^.

To directly assess if the increases in fluorescence in the presence of sugars were due to AGE formation, ELISA was used to detect the representative AGE compound CML. CML increased in these reactions, and the level in samples incubated with ribose was up to nearly seven-fold higher than those in controls incubated without sugars for 4 weeks (Fig. 2b). Compared to ribose, the amounts of CML produced with glucose or fructose were low, similar to CML in the control; this finding is concordant with a previous report^29^.

### Autofluorescence as a biomarker of aging and AGEs in vivo

**Fig. 3:**
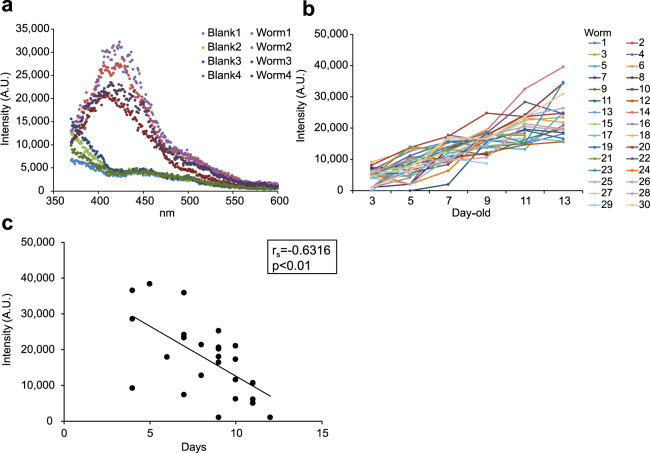
Fluorescence spectrophotometry of individual worms measured by the noninvasive wrap-drop method. Randomly selected worms (wild-type N2) were washed with M9 buffer and then placed in 1.0 µL of M9 buffer on cling film stretched over a 384-well black plate. The autofluorescence in the body of each worm was captured with a multimode grating microplate reader to test whether the fluorescence was detected not only from extracted proteins but in living worms. **a** Data for four 13-day-old worms were produced by a fluorescence spectrophotometer (ex 340/em 360–600). The highest fluorescence was found at a similar wavelength for autofluorescence of extracted proteins. **b** Changes in fluorescence intensity of individual worms over time from 3 days old to 13 days old (ex 340/em 430). After measurement, each worm was individually maintained on a plate covered with OP50 at 25 °C. Each assay was carried out on a minimum of 20 worms and repeated twice; data for worms that died within 2 days were omitted to exclude the influence of death fluorescence. **c** Life expectancy of each 13-day-old worm was inversely associated with the intensity of autofluorescence (ex 340/em 430). Autofluorescence of 13-day-old worms (*n* = 27) was measured, and each intensity was plotted against the period (days) that the worm survived afterward. *r*_*s*_ indicates Spearman’s rank-correlation coefficient. The *p* value was calculated using the test of no correlation.

We next tested whether the fluorescence could be detected not only from extracted proteins but also in living worms. When the autofluorescence of individual worms was read on plastic wrap film stretched on 384-well plates with a multimode grating microplate reader, the spectrophotometry results were similar to those of extracted proteins (Fig. 3a). The individual intensity of blue fluorescence increased over time (Fig. 3b and Supplementary Fig. 2), while no red fluorescence was detected with this method. Since blue fluorescence (340/430 or 350/460 nm) has been previously reported to be associated with death^14,15^, the analysis was performed by excluding data obtained within 2 days prior to an animal’s death. However, the blue fluorescence of the worms still increased over time: the increase of fluorescence must therefore be partially independent of the death fluorescence. To test if the autofluorescence could be a biomarker of senescence, we examined how many days the worms could survive after the autofluorescence was measured on 13-day-old worms. The life expectancy of each worm was inversely related to the intensity of autofluorescence (Fig. 3c).

**Fig. 4:**
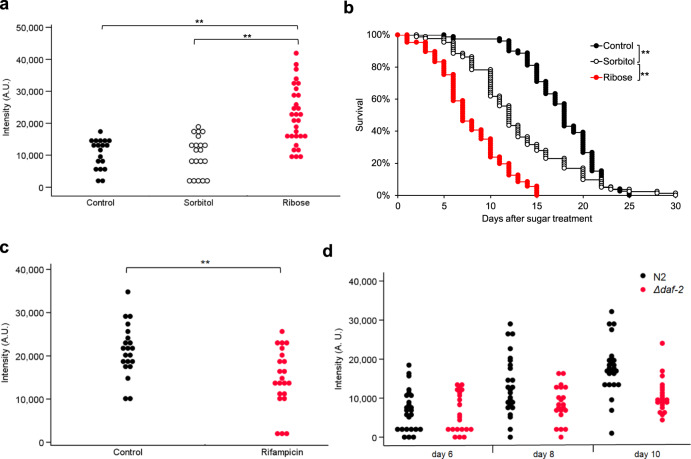
Effects of AGEs-accelerating sugar, an AGEs blocker, and the lifespan-extending mutation *daf-2* on blue fluorescence in individual worms. **a** Autofluorescence (ex 340/em 430) of individual worms (wild-type N2) that were incubated for 4 days on mNGM containing 400 mM ribose (*n* = 30) or 400 mM sorbitol (*n* = 20) to match the osmolality of high ribose; the number of control worms was 19. The autofluorescence values were compared using the nonparametric Steel–Dwass method (**p < 0.01). **b** Survival curve of worms (wild-type N2) grown in the presence of supplemental ribose (*n* = 74); the numbers of worms provided as the control and those cultured with sorbitol were 79 and 84, respectively. Worms were 3 days old on Day 0. Nematode survival was calculated by the Kaplan–Meier method, and the statistical significance of differences was tested by the log-rank test (**p < 0.01). **c** Autofluorescence (ex 340/em 430) in worms (wild-type N2) grown in the presence of rifampicin (*n* = 22), an AGEs blocker, or DMSO as the solvent control (*n* = 21). The autofluorescence values were compared using Student’s *t* test (**p < 0.01). **d** The longevity mutant *daf-2* (*n* = 21) showed significantly lower autofluorescence (ex 340/em 430) than the wild-type N2 (*n* = 25), while the fluorescence intensity of the wild-type increased with aging. Repeated measure two-factor ANOVA indicated that the age and the strains of worms significantly influenced the autofluorescence (*p* < 0.01).

The fluorescence intensity was also elevated in worms grown with ribose (Fig. 4a); the lifespan of these worms was significantly shorter than that of control worms (Fig. 4b). In contrast, worms grown on medium containing rifampicin, an AGEs blocker, emitted less fluorescence compared to control worms grown without rifampicin (Fig. 4c); the prolongevity effect of rifampicin has been previously reported^30^. Furthermore, *daf-2*, a representative prolongevity mutant, also autofluoresced less compared to the wild-type (Fig. 4d), while the fluorescence intensity of the wild-type increased with aging.

**Fig. 5:**
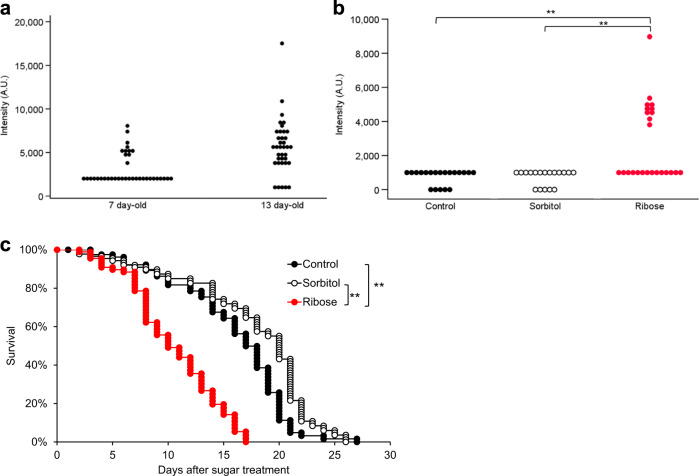
Blue fluorescence from *kynu-1* mutants, which should not emit death fluorescence. **a** Fluorescence intensity (ex 340/em 430) of 7-day-old and 13-day-old *kynu-1* mutants (*n* = 37 each); older worms still emitted more fluorescence than younger worms, in spite of the *kynu-1* mutation. However, no significant difference was detected in the autofluorescence values by the Mann–Whitney *U* test. **b** A 7-day-old worm maintained with ribose emitted more blue fluorescence in spite of the *kynu-1* mutation. Blue fluorescence of *C. elegans* grown with ribose (*n* = 24) or sorbitol (*n* = 18) was compared with that of control worms without the sugars (*n* = 20). The autofluorescence values were compared using the nonparametric Steel–Dwass method (**p < 0.01). **c** Survival curves of *C. elegans* grown with ribose (*n* = 62) or sorbitol (*n* = 84) were compared with that of control worms without the sugars (*n* = 64). Worms were 3 days old on Day 0. Nematode survival was calculated by the Kaplan–Meier method, and survival differences were tested for significance by use of the log-rank test (**p < 0.01).

A portion of the fluorescence is expected to be derived from compounds produced in association with animal death. The blue fluorescence could be the death marker that Coburn et al. observed in *C. elegans* for several hours before and after death; the blue light would come from anthranilic acid that is formed immediately after worms die^14^. Reportedly, death fluorescence is emitted by the glycosylated form of anthranilic acid produced by the kynurenine pathway^14^. *kynu-1* mutant worms, which lack the kynureninase, were accordingly expected not to emit the death fluorescence. However, even in the presence of this mutation, aged worms still appeared to emit higher fluorescence than younger worms (Fig. 5a), and worms grown on nematode growth medium (NGM) supplemented with ribose exhibited significantly higher fluorescence than those grown in the absence of supplemental ribose or with the control sugar sorbitol (Fig. 5b). The lifespan of the worms maintained with ribose was obviously shorter than that of the others (Fig. 5c), suggesting pro-aging effects of AGEs in worms.

### Identification of the autofluorescent materials

To identify substances that fluoresced in aging animals, an LC-MALDI assay was performed on the extracted proteins. The six most-abundant proteins found in each sample are listed in Table 1; notably, vitellogenins and elongation factors were enriched among the top-45 fluorescing proteins in older worms. Furthermore, using high-resolution MS and the Mascot computational environment, we searched for proteins that were more abundant in 15-day-old worms than in 3-day-old worms, and identified 180 proteins (Supplementary Tables 1–3). Although vitellogenins again were enriched (present at six-fold higher levels in older worms than in young adults), the levels of elongation factors were similar in the two groups(Supplementary Fig. 3).

**Table 1:** Proteins that were found more in older worms than in young worms.

	3-day-old	17-day-old
1	Myosin, essential light chain	Vitellogenin-6
	OS = *Caenorhabditis elegans*	OS = *Caenorhabditis elegans*
	GN = mlc-3 PE = 2 SV = 1	GN = vit-6 PE = 1 SV = 5
2	Fatty-acid and retinol-binding protein 2	Vitellogenin-2
	OS = *Caenorhabditis elegans*	OS = *Caenorhabditis elegans*
	GN = far-2 PE = 1 SV = 1	GN = vit-2 PE = 1 SV = 5
3	Paramyosin	Vitellogenin-5
	OS = *Caenorhabditis elegans*	OS = *Caenorhabditis elegans*
	GN = unc-15 PE = 1 SV = 1	GN = vit-5 PE = 2 SV = 2
4	Myosin-4	Elongation factor 1 alpha
	OS = *Caenorhabditis elegans*	OS = *Caenorhabditis elegans*
	GN = unc-54 PE = 1 SV = 1	GN = eft-3 PE = 2 SV = 1
5	Tropomyosin isoforms a/b/d/f	Myosin-4
	OS = *Caenorhabditis elegans*	OS = *Caenorhabditis elegans*
	GN = lev-11 PE = 1 SV = 1	GN = unc-54 PE = 1 SV = 1
6	Actin, nonmuscle	Probable elongation factor 1-beta/1-delta 2
	OS = *Halocynthia roretzi*	OS = *Caenorhabditis elegans*
	GN = CA1 PE = 3 SV = 1	GN = Y41E3.10 PE = 1 SV = 4

*GN* gene name, *PE* protein existence (PE1 indicates that there is clear experimental evidence for the existence of the protein; PE2 means that the existence of a protein has not been strictly proven but that expression data indicate the existence of a transcript; PE3 indicates that the existence of a protein is probable because clear orthologs exist in closely related species), *SV* sequence version.

**Fig. 6:**
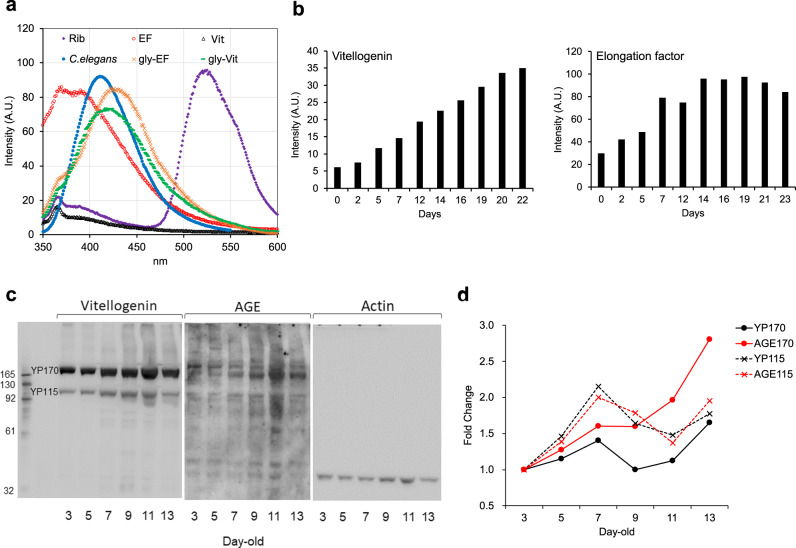
Autofluorescence of vitellogenin (Vit) and elongation factor (EF). **a** Spectrophotometry (ex 325/em 350–600) of vitellogenin and elongation factor before and after glycation in vitro by ribose for 14 days: gly-Vit and gly-EF are glycated vitellogenin and glycate elongation factor, respectively. Riboflavin (Rib) is shown as a representative fluorescent compound. Extract from 17-day-old worms (wild-type N2) is shown for comparison (*C. elegans*). **b** In vitro glycation of vitellogenin and elongation factor by ribose yielded increases in autofluorescence over time. **c** Western blotting analysis of AGE and vitellogenins in samples extracted from worm (wild-type N2) populations of different ages. Blots derived from the same gel and were processed with each antibody in order. **d** Quantification of vitellogenins and AGEs from western blotting using densitometry. Experiments was repeated twice, and the data from a representative experiment are shown. Both line and dotted line in red color show the fold changes of AGEs, while those in black color indicate amounts of vitellogenins. Closed circles and crosses are for the data of bands matching to YP170 and YP115, respectively.

To verify whether these proteins could be glycated to form autofluorescent AGEs, purified elongation factor and vitellogenin were incubated with ribose in vitro. The fluorescence emitted from glycated vitellogenins exhibited spectra similar to those obtained with crude extracts recovered from the 17-day-old worms (Fig. 6a). Although riboflavin is known to fluoresce, we found that the emission wavelength of riboflavin was distinct from that of worm extracts. The fluorescence of glycated vitellogenin and elongation factor were six- and three-fold (respectively) those of the non-glycated proteins by 23 days in vitro (Fig. 6b).

The AGEs detected by western blotting with anti-CML antibody also increased over time from 3-day-old to 13-day-old worms in vivo (Fig. 1c). By simultaneous western blotting with anti-vitellogenin antibodies, we found that the anti-CML signals were the same sized bands as vitellogenins (Fig. 6c). Although the vitellogenins YP170 and YP115 accumulated with age during this period, the intensity of the AGE signals was enhanced higher than the increase in the amount of vitellogenin YP170 (Fig. 6d).

**Fig. 7:**
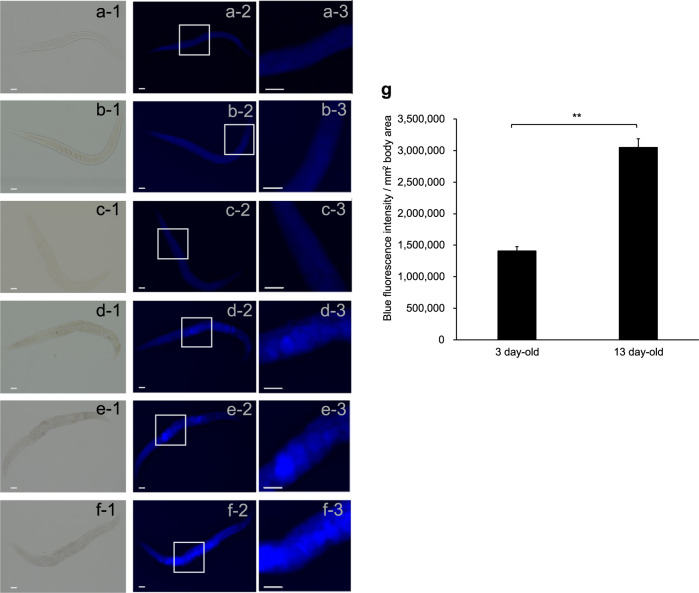
Fluorescence images of 3-day-old and 13-day-old *kynu-1* mutants. **a**–**c** Three-day-old worms and **d**–**f** 13-day-old worms, with raw images in column 1. To exclude the possible effects of death fluorescence that starts from 2 days before the worms’ demise, the mutant *kynu-1* was used. Autofluorescence is seen in the images in columns 2 and 3. Photos in column 3 were magnified from the indicated (by rectangles) areas of the images in column 2. Aged worms (13-day-old) autofluoresced significantly higher than young worms (3-day-old). Each scale bar indicates 50 μm. **g** Quantification of blue fluorescence using ImageJ and ImageQuant TL software. Each bar represents the average values of fluorescence intensity per 1 mm^2^ of nine worms. ** indicates a statistically significant difference between 3-day-old and 13-days worms at a *p* value < 0.01. Error bars represent the SE.

Fluorescence microscopy revealed that 13-day-old worms (Fig. 7d–f, columns 2 and 3) emitted significantly more intense blue light than 3-day-old young adults (Fig. 7a–c, columns 2 and 3; Fig. 7g); the gonads also looked brighter (Fig. 7d–f, columns 1 and 2). CML is a representative AGE, although CML is not itself fluorescent. Pentosidine, another AGE, is known to be fluorescent. However, immunostaining with neither anti-CML nor anti-pentosidine antibodies provided prominent differences between old and young worms (data not shown). Anti-CML however showed diffuse spreading across tissues, while anti-pentosidine hardly showed this.

## Discussion

Previously, autofluorescence has been used to monitor lipofuscin, the age pigment, as a biomarker of senescence^4,13^. Although we observed red fluorescence indicative of lipofuscin as seen in previous studies^9^, blue fluorescence was detected at an earlier stage of life when worms were examined using an M165 FC fluorescence stereomicroscope (Leica Microsystems, Tokyo, Japan) (data not shown). The purpose of the present study was to examine if the blue autofluorescence could serve as a more sensitive marker for tracing the senescence of individual worms.

The fluorescence measured in individual worms increased with age; the less worms fluoresced, the longer their life expectancy. Thus, the blue fluorescence may provide an alternative biomarker for tracing senescence. However, Pincus et al. reported that worms that subsequently died (within 24 h) fluoresced due to the biosynthesis of kynurenine; those researchers named this blue light “death fluorescence”. Reportedly, death fluorescence reflects emission by a glycosylated form of anthranilic acid produced by the kynurenine pathway; the autofluorescing material is detected in lysosome-like gut granules^31^. This blue light could be the same death marker that Coburn et al. observed in *C. elegans* for several hours before and after death, and that was reportedly seen in both young worms subjected to lethal injury and worms dying naturally of old age. Indeed, we observed that the *kynu-1* mutant, which lacks kynureninase activity, autofluoresced less than the wild-type. However, our results indicated that some portion of the blue fluorescence is associated with aging rather than with death. Notably, as shown in Fig. 3b, aging worms exhibited increased blue fluorescence even when excluding data obtained within the 2 days preceding death. Furthermore, worms still fluoresced more with aging, irrespective of the state of the *kynu-1* gene. Reduced fluorescence in the *kynu-1* mutant should thus be due to the loss of acceleration of AGE synthesis by kynurenins rather than the lack of death fluorescence^32^.

Certain AGE compounds are fluorescent^27^. We inferred that the blue fluorescence may include emission from those AGEs, separate from that attributable to death fluorescence. When extracted worm proteins were incubated with sugars in vitro, the fluorescence intensity and the levels of AGEs increased over time. Worms cultured on medium containing ribose fluoresced more than control animals grown in the absence of supplemental ribose, even when *kynu-1* was mutated. In contrast, worms cultured in the presence of rifampicin, a known inhibitor of AGEs production, exhibited decreased levels of blue fluorescence. Indeed, fluorescence microscopy indicated that 13-day-old worms emitted more blue light than 3-day-old young adults. Immunostaining with anti-CML or anti-pentosidine antibodies failed to show the presence of diffusely spreading AGEs in a distribution pattern resembling that of blue fluorescence. The fluorescence could originate from other abundant fluorescent AGEs such as vesperlysine A, LM-1, and argpyrimidine^33,34^.

To provide the yolk for oocysts, *C. elegans* hermaphrodites consume their own intestinal biomass, which results in intestinal atrophy and ectopic yolk deposition in later life. Vitellogenins (yolk proteins) were present at two-fold higher levels in old worms (compared to younger animals), as reported before^35^, and exhibited six-fold greater fluorescence after glycation in vitro. These data suggested that vitellogenins themselves would generate 12-fold increased fluorescence in older worms compared to young adults theoretically.

Western blotting showed that the main bands reacting with the anti-CML antibody were the yolk proteins. This finding matches the previous reports of Nakamura et al., who detected heavy glycation of vitellogenin^36^, and Golegaonkar et al., who detected decreased glycation in vitellogenin-2, vitellognin-6, and elongation factor 1 alpha in rifampicin-treated nematodes^30^. Reportedly, vitellogenins act as antioxidants and contribute to the longevity of honey bee queens^37^. In *C. elegans*, vitellogenins play a crucial role in stress resistance^38^. Since antioxidants can be oxidized easily themselves, the accumulation of glycated vitellogenin may impair protection from oxidative damage, resulting in the inverse relationship between life expectancy and the intensity of blue fluorescence observed in the present study. However, Sornda et al. recently reported that lifespan is unrelated to oxidative stress resistance mediated by YP115/YP88 (vitellogenin-6)^39^. Those researchers proposed that the accumulation of vitellogenin YP170, derived from vitellogenins 1–5, causes intestinal atrophy and decreased lifespan, while the accumulation of YP115/YP88 might retard intestinal atrophy and extend lifespan. Glycation of vitellogenin-6 and the resulting dysfunction therefore may contribute to senescence. Although elongation factors fluoresced three-fold more intensely after in vitro glycation, the amounts of these proteins were similar between young and old worms. Therefore, the contribution of this factor to enhanced autofluorescence may be smaller than that attributable to vitellogenins.

*C. elegans* has been used generally as a model to study senescence and anti-senescence interventions. We proposed the use of the worm as a model to investigate the prolongevity effects of lactic acid bacteria^8^. Given the demonstration (in the present study) that blue fluorescence in nematodes is a possible indicator of AGEs accumulation, we propose that *C. elegans* hermaphrodites can serve as a model for investigating the utility of anti-senescence interventions that act via the suppression of AGEs production. In contrast, the autofluorescence is unlikely to be a biomarker of senescence for male worms because vitellogenins must be scarce.

## Methods

### Nematodes

*C. elegans* Bristol strain N2 and its derivative mutant strains were kindly provided by the Caenorhabditis Genetics Center, University of Minnesota. The mutations used in this study were CB1370 *daf-2 (e1370)* and CB1003 *kynu-1 (e1003)*. Nematodes were maintained and propagated on NGM according to standard techniques^40^. Worm eggs were recovered from adult worms after exposure to a sodium hypochlorite/sodium hydroxide solution as previously described^41^. Egg suspensions were incubated overnight at 25 °C to allow hatching, and the suspension of L1-stage worms was centrifuged at 156 × *g* for 1 min. The supernatant was removed, and the remaining larvae were transferred onto fresh peptone-free modified NGM (mNGM) plates covered with *E. coli* strain OP50 (OP50). Strains were grown on mNGM agar plates at 25 °C except for the *daf-2* strain, which was grown at 20 °C until L4 stage. All assays were begun with young adult worms that started egg laying at 25 °C. No ethical approval was required for the nematodes.

### Bacterial strains

OP50 was used as the internationally established food of nematodes. Tryptone soya agar (Nissui Pharmaceutical, Tokyo, Japan) was used to culture OP50. Bacteria (100 mg wet weight) were suspended in 0.5 mL of M9 buffer; a 50-µL aliquot of the bacterial suspension then was spread on the mNGM in 5.5-cm-diameter plates unless otherwise stated.

### Multivariate analysis of autofluorescence

Populations of 100 adults each were recovered at 3 and 17 days of age, washed three times, concentrated in 3 µL of M9 buffer, and stored frozen at –80 °C until use. Before extraction, 3 µL of lysis buffer (consisting of 50 mM Tris-HCl buffer (pH 7.5), 150 mM NaCl, 0.1% sodium dodecyl sulfate (SDS), 1% Triton X-100, 1 mM phenylmethylsulfonyl fluoride (PMSF), and 2% 2-mercaptoethanol) and 4 µL of 15% SDS were distributed to each tube. Samples then were subjected to five freeze-thaw cycles to release protein. Photoluminescent spectra were collected with a fluorescence spectrophotometer (Hitachi FL-4500) with data interpreting software (FL Solutions ver. 2.0). Fluorescence properties of nematode suspensions were analyzed using excitation emission matrix (EEM) fluorescence spectroscopy. Multivariate analysis (single-wavelength excitation with multiple-wavelength emission, and synchronous-scanning fluorometry) yielded EEM plots consisting of single-scan excitation, and the synchronous fluorescence spectra of each series were drawn.

### Fluorescence spectra of aging worms

Worms (3–13 days old) were recovered from growth medium containing 2’-deoxy-5-fluorouridine (Tokyo Chemical Industry, Tokyo, Japan). This compound blocks embryonic development of progeny, thereby precluding contamination by offspring. Individual aging worms were collected in tubes, washed five times, concentrated in 3 µL of M9 buffer, and stored frozen at –80 °C until use. Before extraction, 100 µL of lysis buffer (consisting of 50 mM Tris-HCl buffer (pH 7.5), 150 mM NaCl, 10 mM dithiothreitol (DTT), 1 mM PMSF, and 1 µL protease inhibitor cocktail (Sigma, St. Louis, MO, USA)) were added to each tube. Samples then were ground using a Mini Cordless Grinder (Funakoshi, Tokyo, Japan) to release protein. Protein contents were quantitatively measured using the Pierce™ 660 nm Protein Assay Reagent (Thermo Fisher Scientific Meridian, Waltham, MA, USA) and a NanoDrop OneC instrument (Thermo Fisher Scientific). The fluorescence spectrum was determined for each 30-µL sample (containing 1.5 μg of total protein) using a multimode grating microplate reader model SH-9000Lab with SF6 Data Treatment Software (Corona Electric, Ibaraki, Japan). Each measurement was carried out three times.

### Measurement of autofluorescence of individual living worms (wrap-drop method)

Randomly selected worms were washed with M9 buffer, and then individual worms were placed in 1.0 µL of M9 buffer on Saran Wrap cling film (Asahi Kasei, Tokyo, Japan) stretched over a 384-well black plate (STEM, Tokyo, Japan). Small hollows were formed on each well using a replicator (Watson, Kobe, Japan). This modification was to recover worms safely after the measurement so that the age-dependent change of autofluorescence of each worm could be traced. The cling film was chosen because it autofluoresced less than other commercially available films. However, the blank (M9 buffer only) data were checked three times for each well, considering the fluctuation from well to well. Minimal detection limits and quantifiable limits were determined on the basis of blank data on each day as μ (mean of the blank) + 3.29σ (standard deviation) and μ + √2 × 10σ, respectively. The autofluorescence in the body of each worm was captured with a multimode grating microplate reader. After measurement, each worm was individually maintained on a 4.0-cm diameter plate covered with OP50 (2 mg/10 μL M9 buffer) at 25 °C. Each assay was carried out on a minimum of 20 worms and repeated twice.

### Life expectancy measurement

Worms were maintained on mNGM plates covered with OP50 at 25 °C until 13 days old. After evaluation by fluorescence assay, 30 worms each were moved onto separate new mNGM plates covered with OP50. The plates were incubated at 25 °C, and live and dead worms were scored every 24 h. A worm was considered dead when it failed to respond to a gentle touch with a worm picker.

### AGEs after in vitro glycation

Protein extraction was performed as previously described. Three-day-old worms were used for artificial glycation. The protein samples (2 mg/mL) were incubated with 500 mM glucose, 100 mM ribose, or 500 mM fructose (final concentration). All samples were incubated at 37 °C for 0–4 weeks. Aliquots (50 μL) were dispensed to the wells of a polystyrene plate (Sumitomo Bakelite, Tokyo, Japan) and incubated for 2 h at 37 °C. After removal of the sample solution, the plate was washed with PBS-T (phosphate-buffered saline (PBS), 0.05% Tween 20); 250 μL of 3% skim milk was added to each well, and the plate was incubated for 2 h at 37 °C. Each well then was washed with PBS-T, and 100 μL of an anti-AGE (anti-CML) monoclonal antibody (Clone No. 6D12, Trans Genic, Fukuoka, Japan; 75 ng/mL) was dispensed to each well; the plate then was incubated at room temperature for 1 h. After washing with PBS-T, 100 μL of a peroxidase-conjugated goat anti-mouse IgG antibody (Rockland Immunochemicals, Inc., Limerick, PA, USA; 1:150,000) was dispensed to each well, and the plate was incubated at room temperature for 1 h. After washing with PBS-T, 100 μL of a working solution (tetramethylbenzidine (TMB) staining solution:peroxide solution = 1:1; ELISA POD substrate TMB kit, Popular, Nacalai Tesque, Kyoto, Japan) was dispensed to each well, and the plate was maintained at room temperature in the dark until quenching by the addition of 100 μL of 1 mol/L sulfuric acid per well. The absorbance of each well at 450 nm (sub: 650 nm) then was measured immediately with a microplate reader. ELISA was performed three times for each of the test conditions.

### Western blotting for aging worms

Samples were treated with 4× Bolt™ LDS Sample Buffer (Thermo Fisher Scientific) and 10× Bolt™ Sample Reducing Agent (Thermo Fisher Scientific), and each sample containing 1.5 μg of total protein was run on a Bolt™ 4–12% Bis-Tris Plus Gel (Thermo Fisher Scientific). Then the proteins were transferred from the gel to a polyvinylidene fluoride membrane (ATTO, Tokyo, Japan) and blocked with 5% skim milk in PBS-0.1% Tween 20 for 1 h. The membrane was incubated overnight at 4 °C with an anti-AGE (anti-CML) monoclonal antibody (Clone No. 6D12 at 1:1000), washed with PBS-T, and then incubated at room temperature for 1 h with a peroxidase-conjugated goat anti-mouse IgG antibody (1:25,000). After washing with PBS-T, the membrane was incubated with the ECL prime western blotting detection reagent (GE Healthcare UK, Little Chalfont, England) and scanned using a ChemiDoc Touch Imaging System (Bio-Rad, Hercules, CA, USA) or ImageQuant LAS 500 (GE Healthcare UK). Membranes then were washed with PBS-T, incubated with Western BLoT Stripping Buffer (Takara, Shiga, Japan) for 30 min at room temperature, and washed again with PBS-T. The membrane was re-probed by anti-vitellogenin antibodies YP115 and YP170 (each 1:10,000) at 4 °C overnight^42^, washed with PBS-T, and incubated with goat anti-rat IgG antibody conjugated with peroxidase (1:2000) (Proteintech, Rosemont, IL, USA) at room temperature for 2 h. For loading controls, the membranes were re-probed using an anti-actin antibody (Clone No. C4; Merck KGaA, Darmstadt, Germany) and peroxidase-conjugated anti-mouse antibody (GE Healthcare UK). The densities of the vitellogenin (YP170 and YP115) and CML bands in each lane were normalized against those of the actin bands in the respective lane. Band densities were analyzed using ImageQuant TL software (GE Healthcare). Each assay was performed twice.

### Effects of ribose and rifampicin on AGEs in vivo

Three-day-old worms were cultured on mNGM containing 400 mM ribose or 400 mM sorbitol to match the osmolarity of high ribose; worm food was provided as heat (100 °C, 10 min)-killed OP50 to prevent the bacteria from fermenting the sugar. To prevent contamination by progeny, worms were transferred to fresh plates daily for 4 days until they had completely finished egg laying (at 7 days old). To assess the influence of ribose, we performed survival assays and measured autofluorescence. A worm was considered dead when it failed to respond to a gentle touch with a worm picker. Worms that died as a result of adhering to the wall of the plate were not included in the analysis and censored. In a separate experiment, mNGM containing 50 µM (final concentration) rifampicin (dissolved in DMSO) was used. Each assay was repeated twice.

### Identification of old worm-specific proteins

Mass spectrometry analysis of extracts from 3- and 17-day-old nematodes was performed on an AB SCIEX 5800 LC-MALDI-TOF mass spectrometer (Newark, NJ, USA) with Protein Pilot version 4.0. Both matrices were digested by trypsin and were mixed with a matrix solution (7 mg/L of α-cyano-4-hydroxycinnamic acid in 0.1% (v/v) trifluoroacetic acid (TFA), 70% (v/v) acetonitrile (ACN)). The flow rate used for separation was 300 nL/min, and separation was achieved using a linear gradient of two mobile phases (0.1% (v/v) TFA in 2% ACN (solvent A) and 0.1% (v/v) TFA in 80% ACN (solvent B)) in the following proportions: A/B = 100/0–50/50 (60 min), A/B = 50/50–0/100 (20 min), A/B = 0/100 (10 min). A MALDI-TOF Mass system was used to obtain a peptide-mass fingerprint. Peptide matching and protein searches were performed against the Swiss-Prot version 57.0 database.

Extracted proteins were dissolved in 50 mM ammonium bicarbonate (AmBic) to yield protein concentrations between 0.1 and 1 µg/µL. In order to maximize the solubility of proteins, a calculated volume of Waters Rapigest (Waters Corporation, Milford, MA, USA) was added to yield final concentrations of 0.1–0.2% Rapigest in pre-digestion samples. The samples were heated at 40 °C with shaking for 10 min; the contents then were centrifuged, and the resulting pellet was suspended in AmBic containing 10 mM DTT. The samples were heated at 80 °C for 15 min and pelleted at room temperature. An aliquot of 200 mM iodoacetamide (IAM) in 50 mM AmBic was added to each sample to yield a final IAM concentration of 20 mM (in the presence of a 2× molar excess of DTT); the mixture then was incubated in the dark at room temperature for 30 min to permit the alkylation reaction to proceed. Trypsin in 50 mM AmBic was mixed with each solution at 1:50 (trypsin:protein concentration). Samples were digested for at least 4 h or overnight at 37 °C with shaking. Following centrifugation, TFA and ACN were added to yield final concentrations of 0.5–1.0% TFA and 2% ACN by volume. Samples were shaken for 2 h at 60 °C. After centrifugation at 22,200 × *g* for 5 min, the supernatant was pipetted into an autosampler vial. Proteins were digested with trypsin prior to analysis by reverse-phase liquid chromatography using an Ultimate 3000RSnanoLC system (Thermo Fisher Scientific) coupled to an ESI-Q-TOF system (Impact II Bruker Daltonics). The yeast alcohol dehydrogenase (ADH1_YEAST) protein was spiked in the samples as an internal standard; spiking was performed to provide a quantity of approximately 50 fmol of the standard on the column.

### Autofluorescence after in vitro glycation

Vitellogenin in PBS solution (0.053 µM) was purchased from Biosense Laboratories AS (Bergen, NORWAY). Elongation factor solution (1.17 µM) was purchased from Abnova Corporation (Taipei City, Taiwan). These solutions were glycated with 0.1 mM ribose for 23 days at 37 °C. Riboflavin was purchased from Sigma (Tokyo, Japan) and dissolved in PBS at 0.1 mM. Each solution was measured by fluorescence spectrophotometry under the same conditions.

### Immunofluorescence by young and old worms

Age-synchronized CB1003 fed OP50 were incubated at 25 °C. Three-day-old and 13-day-old adults were permeabilized using the Bouin’s tube fixation protocol^43^.

Samples were fixed in Bouin’s fixative with methanol/β-mercaptoethanol at room temperature for 30 min. To crack the cuticle, worms were placed in isopropanol and stored at –80 °C for 10 min, then incubated at room temperature for another 30 min. The fixative solution was removed and replaced with BTB solution (25 mM borate buffer, 0.5% Triton X-100, 2% β-mercaptoethanol) at room temperature for 1 h; incubation in BTB was repeated for a total of three times. The worms then were transferred from BTB to BT (25 mM borate buffer, 0.5% Triton X-100), incubated in antibody buffer solution (1× PBS, 0.5% Triton X-100, 0.1 mM EDTA, 0.1% bovine serum albumin (BSA), 0.05% sodium azide, pH 7.2) at room temperature for 1 h, and blocked with antibody buffer solution containing 10% goat serum (Cosmo Bio, Tokyo, Japan) at 4 °C overnight. The primary antibodies consisted of mouse monoclonal anti-AGE (anti-CML) antibody (Clone No. 6D12; 1:125) and anti-pentosidine antibody (Clone No. PEN-12, Trans Genic; 1:50). The secondary antibody consisted of Alexa Fluor 555-conjugated goat anti-mouse antibody (Abcam, Cambridge, England; 1:100). All antibody dilutions were performed using antibody buffer containing 0.5% BSA. Samples were mounted onto glass slides using VECTASHIELD antifade mounting medium (Vector Laboratories, Burlingame, CA, USA).

### Fluorescence microscopy

Fluorescence images of 3- or 13-day-old adult worms, mounted on glass slides as described above, were recorded using an inverted fluorescence microscope (BZ-X700; Keyence) with a 10× objective lens (CFI Plan Fluor DL 10×/0.30). Autofluorescence of worms and red fluorescence from Alexa Fluor 555 were viewed with BZ-X DAPI (excitation wavelength (Ex), 360 ± 20 nm; emission wavelength (Em), 460 ± 25 nm) and TRITC (Ex, 545 ± 12.5 nm; Em, 605 ± 35 nm) filters, respectively. Section images were captured by the Sectioning mode with one-dimensional slit type with width 32, and Z-stack pitch 0.7 μm for 40-μm-thick samples. To quantify the blue fluorescence, the fluorescence intensities measured by ImageQuant TL software (GE Healthcare) were normalized to density values per 1 mm^2^ of a worm’s projection area. The body size was determined with Adobe Photoshop Elements and ImageJ software developed by the National Institutes of Health. In this system, the area of a worm’s projection was estimated automatically and used as an index of body size.

### Statistical analysis

Correlation of the life expectancy was calculated using Spearman’s rank-correlation coefficient. The autofluorescence levels after in vitro glycation were compared using two-factor factorial ANOVA and Scheffe’s *F* test. The ELISA values after in vitro glycation were compared using single-factor ANOVA and the Dunnett test for multiple comparisons. Nematode survival was calculated by the Kaplan–Meier method, and survival differences were tested for significance by use of the log-rank test. The autofluorescence values following rifampicin treatment and aging of the *daf-2* and *kynu-1* mutants were compared for each using Student’s *t* test, repeated measure two-factor ANOVA, and the Mann–Whitney *U* test. The autofluorescence values following ribose treatments for N2 or the *kynu-1* mutant were compared using the nonparametric Steel–Dwass method. The densities from autofluorescence microscopy were compared using Student’s *t* test. Where significance was observed, data were classified as *p < 0.05 and **p < 0.01. All statistical analyses were performed with Microsoft Excel supplemented with the add-in software +Statcel 3 (OMS, Tokyo, Japan) and JSTAT for Windows (Nankodo, Tokyo, Japan).

### Reporting summary

Further information on research design is available in the Nature Research Reporting Summary linked to this article.

## Supplementary information

Reporting Summary

Supplemental Material

## Data Availability

The datasets generated during the current study are available from the corresponding author on reasonable request.
