# Supplementary material for: Autofluorescence as a noninvasive biomarker of senescence and advanced glycation end products in Caenorhabditis elegans
Source: NPJ Aging Mech Dis. 2021 Jun 7;7:12. doi: 10.1038/s41514-021-00061-y (PMC8184826; doi:10.1038/s41514-021-00061-y)
Supplement: Supplementary file 1 — Reporting Summary [file 41514_2021_61_MOESM1_ESM.pdf]

nature research

Corresponding author(s): Yoshikazu Nishikawa, NPJAMD-00241R

Last updated by author(s): Feb 3, 2021

Reporting Summary

Nature Research wishes to improve the reproducibility of the work that we publish. This form provides structure for consistency and transparency in reporting. For further information on Nature Research policies, see our Editorial Policies and the Editorial Policy Checklist.

Statistics

For all statistical analyses, confirm that the following items are present in the figure legend, table legend, main text, or Methods section.

n/a	Confirmed	
☐	☒	The exact sample size (*n*) for each experimental group/condition, given as a discrete number and unit of measurement
☐	☒	A statement on whether measurements were taken from distinct samples or whether the same sample was measured repeatedly
☐	☒	The statistical test(s) used AND whether they are one- or two-sided<br>*Only common tests should be described solely by name; describe more complex techniques in the Methods section.*
☐	☒	A description of all covariates tested
☐	☒	A description of any assumptions or corrections, such as tests of normality and adjustment for multiple comparisons
☐	☒	A full description of the statistical parameters including central tendency (e.g. means) or other basic estimates (e.g. regression coefficient) AND variation (e.g. standard deviation) or associated estimates of uncertainty (e.g. confidence intervals)
☒	☐	For null hypothesis testing, the test statistic (e.g. F, t, r) with confidence intervals, effect sizes, degrees of freedom and P value noted<br>*Give P values as exact values whenever suitable.*
☒	☐	For Bayesian analysis, information on the choice of priors and Markov chain Monte Carlo settings
☒	☐	For hierarchical and complex designs, identification of the appropriate level for tests and full reporting of outcomes
☒	☐	Estimates of effect sizes (e.g. Cohen's d, Pearson's r), indicating how they were calculated

Our web collection on statistics for biologists contains articles on many of the points above.

Software and code

Policy information about availability of computer code

Data collection	Band densities in western blotting and intensities of fluorescence microscopy were analyzed using ImageQuant TL software (GE Healthcare).<br>The body size of each worm was determined with Adobe Photoshop Elements and ImageJ software developed by the National Institutes of Health<br>The fluorescence spectrum was determined using a multimode grating microplate reader model SH-9000Lab with SF6 Data Treatment Software (Corona Electric, Ibaraki, Japan)<br>Fluorescence spectrophotometer (Hitachi FL-4500) was with data interpreting software (FL Solutions ver. 2.0).<br>AB SCIEX 5800 LC-MALDI-TOF mass spectrometer (Newark, NJ, USA) was used with Protein Pilot version 4.0.<br>Inverted fluorescence microscope (Keyence BZ-X700) was operated by software (BZ-X Viewer) and captured images were analyzed by software (BZ-X Analyzer).
Data analysis	Microsoft Excel supplemented with the add-in software +Statcel 3 (OMS, Tokyo, Japan) and JSTAT for Windows (Nankodo, Tokyo, Japan).

For manuscripts utilizing custom algorithms or software that are central to the research but not yet described in published literature, software must be made available to editors and reviewers. We strongly encourage code deposition in a community repository (e.g. GitHub). See the Nature Research guidelines for submitting code & software for further information.

Data

Policy information about availability of data

All manuscripts must include a data availability statement. This statement should provide the following information, where applicable:

- Accession codes, unique identifiers, or web links for publicly available datasets
- A list of figures that have associated raw data
- A description of any restrictions on data availability

The datasets generated during the current study are available from the corresponding author on reasonable request.
Raw data on Figures 7A-F are provided as Fig7_A-1_raw_image.tif, Fig7_A-2_raw_image.tif, and so on.

Field-specific reporting

Please select the one below that is the best fit for your research. If you are not sure, read the appropriate sections before making your selection.

☒ Life sciences ☐ Behavioural & social sciences ☐ Ecological, evolutionary & environmental sciences

For a reference copy of the document with all sections, see nature.com/documents/nr-reporting-summary-flat.pdf

Life sciences study design

All studies must disclose on these points even when the disclosure is negative.

Sample size	No statistical method was used to predetermine sample size. Sample size was based on our previous experience and the reports published by laboratories performing these types of assays with C. elegans.
Data exclusions	For lifespan studies, nematodes that died due to internal hatching, crawled off plates or lost were excluded from analysis. As we wrote in the legend of Fig. 3B, data for worms that died within two days were omitted to exclude the influence of death fluorescence, considering the report of Coburn et al (14).
Replication	The results could be reproduced with similar tendencies.
Randomization	Nematodes were assigned into experimental groups randomly. The worms used in this study belonged to a clonal group and were all age-synchronized. They displayed voluntary movement and no symptoms of physiological problem.
Blinding	When these experiments were carried out, the operator was not informed about if the worms belonged to the treatment group or control.

Reporting for specific materials, systems and methods

We require information from authors about some types of materials, experimental systems and methods used in many studies. Here, indicate whether each material, system or method listed is relevant to your study. If you are not sure if a list item applies to your research, read the appropriate section before selecting a response.

Materials & experimental systems

n/a	Involved in the study	
☐	☒	Antibodies
☒	☐	Eukaryotic cell lines
☒	☐	Palaeontology and archaeology
☐	☒	Animals and other organisms
☒	☐	Human research participants
☒	☐	Clinical data
☒	☐	Dual use research of concern

Methods

n/a	Involved in the study	
☒	☐	ChIP-seq
☒	☐	Flow cytometry
☒	☐	MRI-based neuroimaging

Antibodies

Antibodies used	Anti AGEs Monoclonal Antibody Clone No. 6D12 Trans Genic Inc. Cat# KH001 Lot# TG150618<br>Anti Pentosidine Monoclonal Antibody Clone No. PEN-12 Trans Genic Inc. Cat# KH012 Lot#TG091118<br>Anti-Actin Antibody,clone C4 Millipore Cat# MAB1501R Lot# LV1746179<br>Anti-MOUSE IgG (H&L) (GOAT) Antibody Peroxidase Conjugated , Cat# 610-1319 Lot# 33004, Rockland Immunochemicals, Inc.<br>Peroxidase-labeled anti-mouse antibody, Cat# NA931 Lot# 382001, GE Healthcare Bio-Sciences<br>Goat anti-rat IgG (H+L), HRP conjugate, Cat# SA00001-15 Lot# 20000161, Proteintech Group, Inc<br>Goat Anti-Mouse IgG H&L (Alexa Fluor® 555) preadsorbed, Cat# ab150118 Lot# GR299321-5, Abcam<br>Anti-vitellogenin antibodies YP115 and YP170, kind gifts from Prof. Emeritus Thomas Blumenthal from the Dept. of Molecular,

	Cellular, and Developmental Biology, Boulder, University of Colorado
Validation	Anti AGEs Monoclonal Antibody; http://www.transgenic.co.jp/en/products/antibodies-product/transgenic/pdf/KH001.pdf<br>Anti Pentosidine Monoclonal Antibody; http://www.transgenic.co.jp/en/products/antibodies-product/transgenic/pdf/KH012-01.pdf<br>Anti-Actin Antibody; https://www.merckmillipore.com/JP/ja/product/Anti-Actin-Antibodyclone-C4,MM_NF-MAB1501R#anchor_DS<br>Anti-MOUSE IgG (H&L) (GOAT) Antibody Peroxidase Conjugated; https://rockland-inc.com/store/Whole-IgG-Affinity-Purified-Secondary-Antibodies-610-1319-O4L_12096.aspx<br>Peroxidase-labeled anti-mouse antibody; https://www.gelifesciences.com/en/us/shop/protein-analysis/blotting-and-detection/blotting-standards-and-reagents/amersham-ecl-hrp-conjugated-antibodies-p-06260<br>Goat anti-rat IgG (H+L), HRP conjugate; https://www.ptglab.co.jp/products/Goat-anti-rat-IgG--H-L-,-HRP-conjugate-secondary-antibody.htm<br>Goat Anti-Mouse IgG H&L (Alexa Fluor® 555); https://www.abcam.co.jp/goat-mouse-igg-hl-alexa-fluor-555-ab150114.html<br>Anti-vitellogenin antibodies YP115 and YP170, The reference No. 42: Molecular and Cellular Biology 5, 2495-2501 (1985).

Animals and other organisms

Policy information about studies involving animals; ARRIVE guidelines recommended for reporting animal research

Laboratory animals	Caenorhabditis elegans was used in this study: wild type (N2), CB1370(daf-2) and CB1003(kynu-1)
Wild animals	No wild animals were used in this study.
Field-collected samples	No samples collected from the field were used in this study.
Ethics oversight	No ethical approval was required for the nematodes.

Note that full information on the approval of the study protocol must also be provided in the manuscript.
