# Supplementary material for: Autofluorescence as a noninvasive biomarker of senescence and advanced glycation end products in Caenorhabditis elegans
Source: NPJ Aging Mech Dis. 2021 Jun 7;7:12. doi: 10.1038/s41514-021-00061-y (PMC8184826; doi:10.1038/s41514-021-00061-y)
Supplement: Supplementary file 2 — Supplemental Material [file 41514_2021_61_MOESM2_ESM.pdf]

Supplementary Table 1. Proteins identified in 15-day-old worms
(In order of MASCOT score)

Row	Accession	Protein	MW [kDa]	pI	#Alt. Proteins	Scores	#Peptides	SC [%]	RMS90 [ppm]	Rank
1	VIT6_CAEEL	Vitellogenin-6 OS=Caenorhabditis elegans GN=vit-6 PE=1 SV=5	193.2	6.9	1	6712.3 (M:6712.3)	165	61.8	3.66	1
2	VIT2_CAEEL	Vitellogenin-2 OS=Caenorhabditis elegans GN=vit-2 PE=1 SV=5	187.6	6.2	1	5271.4 (M:5271.4)	120	54.5	3.62	2
3	VIT5_CAEEL	Vitellogenin-5 OS=Caenorhabditis elegans GN=vit-5 PE=2 SV=2	186.3	6.6	1	3554.8 (M:3554.8)	88	45.2	3.42	3
4	VIT3_CAEEL	Vitellogenin-3 OS=Caenorhabditis elegans GN=vit-3 PE=1 SV=1	186.4	6.5	1	2698.7 (M:2698.7)	5	3.4	5.40	4
5	VIT4_CAEEL	Vitellogenin-4 OS=Caenorhabditis elegans GN=vit-4 PE=1 SV=3	186.2	6.6	1	2646.7 (M:2646.7)	3	2.9	7.23	5
6	VIT1_CAEEL	Vitellogenin-1 OS=Caenorhabditis elegans GN=vit-1 PE=1 SV=2	187.9	6.5	1	2597.6 (M:2597.6)	3	4.0	7.70	6
7	MYO4_CAEEL	Myosin-4 OS=Caenorhabditis elegans GN=unc-54 PE=1 SV=2	224.6	5.6	1	1454.0 (M:1454.0)	33	22.7	3.85	7
8	HSP7A_CAEEL	Heat shock 70 kDa protein A OS=Caenorhabditis elegans GN=hsp-1 PE=1 SV=2	69.7	5.4	1	908.2 (M:908.2)	22	34.7	4.43	8
9	ACT1_CAEEL	Actin-1 OS=Caenorhabditis elegans GN=act-1 PE=1 SV=1	41.8	5.2	2	906.9 (M:906.9)	21	50.5	3.66	9
10	ACT2_CAEEL	Actin-2 OS=Caenorhabditis elegans GN=act-2 PE=3 SV=3	41.8	5.2	1	766.1 (M:766.1)	1	6.9	2.47	10
11	EF1A_CAEEL	Elongation factor 1-alpha OS=Caenorhabditis elegans GN=eft-3 PE=3 SV=1	50.6	9.1	1	744.5 (M:744.5)	21	43.8	2.75	11
12	TBB2_CAEEL	Tubulin beta-2 chain OS=Caenorhabditis elegans GN=tbb-2 PE=3 SV=1	50.3	4.8	2	732.8 (M:732.8)	17	44.9	4.48	12
13	HSP90_CAEEL	Heat shock protein 90 OS=Caenorhabditis elegans GN=daf-21 PE=1 SV=1	80.2	5.0	1	708.0 (M:708.0)	16	28.1	4.06	13
14	TBA2_CAEEL	Tubulin alpha-2 chain OS=Caenorhabditis elegans GN=tba-2 PE=2 SV=1	49.9	4.9	3	670.8 (M:670.8)	16	36.4	4.65	14
15	EF2_CAEEL	Elongation factor 2 OS=Caenorhabditis elegans GN=eef-2 PE=1 SV=4	94.7	6.1	1	638.4 (M:638.4)	17	23.8	3.80	15
16	CH60_CAEEL	Chaperonin homolog Hsp-60, mitochondrial OS=Caenorhabditis elegans GN=hsp-60 PE=1 SV=2	60.1	5.3	1	632.8 (M:632.8)	14	32.0	3.67	16
17	14331_CAEEL	14-3-3-like protein 1 OS=Caenorhabditis elegans GN=par-5 PE=1 SV=2	28.2	4.7	1	588.1 (M:588.1)	17	55.6	3.70	17
18	U375A_CAEEL	UPF0375 protein C08F11.11 OS=Caenorhabditis elegans GN=C08F11.11 PE=1 SV=1	11.8	5.7	1	581.9 (M:581.9)	14	72.1	4.39	18
19	TCTP_CAEEL	Translationally-controlled tumor protein homolog OS=Caenorhabditis elegans GN=tct-1 PE=1 SV=1	20.5	4.8	1	547.7 (M:547.7)	9	55.8	3.46	19
20	ALF2_CAEEL	Fructose-bisphosphate aldolase 2 OS=Caenorhabditis elegans GN=aldo-2 PE=2 SV=1	38.8	7.7	1	539.5 (M:539.5)	11	35.8	3.45	20
21	PPN1_CAEEL	Papilin OS=Caenorhabditis elegans GN=mig-6 PE=1 SV=1	237.4	4.9	1	502.4 (M:502.4)	10	9.1	6.03	21
22	HSP7D_CAEEL	Heat shock 70 kDa protein D OS=Caenorhabditis elegans GN=hsp-4 PE=1 SV=2	72.2	5.0	1	495.4 (M:495.4)	9	17.7	3.08	22
23	ATPA_CAEEL	ATP synthase subunit alpha, mitochondrial OS=Caenorhabditis elegans GN=H28O16.1 PE=1 SV=1	57.8	9.5	1	490.4 (M:490.4)	13	29.0	3.51	23
24	14332_CAEEL	14-3-3-like protein 2 OS=Caenorhabditis elegans GN=ftt-2 PE=1 SV=1	28.0	4.8	1	480.9 (M:480.9)	5	29.4	2.41	24
25	MYSP_CAEEL	Paramyosin OS=Caenorhabditis elegans GN=unc-15 PE=1 SV=1	101.9	5.3	1	470.8 (M:470.8)	11	16.8	2.72	25
26	SAHH_CAEEL	Adenosylhomocysteinase OS=Caenorhabditis elegans GN=ahcy-1 PE=1 SV=1	47.5	5.9	1	465.2 (M:465.2)	9	30.7	2.62	26
27	F37C4_CAEEL	Protein F37C4.5 OS=Caenorhabditis elegans GN=F37C4.5 PE=1 SV=3	61.4	5.5	1	464.1 (M:464.1)	12	26.4	3.38	27
28	ACON_CAEEL	Probable aconitate hydratase, mitochondrial OS=Caenorhabditis elegans GN=aco-2 PE=1 SV=2	84.0	8.2	1	460.9 (M:460.9)	11	19.9	8.19	28
29	PDI1_CAEEL	Protein disulfide-isomerase 1 OS=Caenorhabditis elegans GN=pdi-1 PE=3 SV=1	53.4	4.6	1	457.0 (M:457.0)	11	33.8	3.47	29
30	CYP7_CAEEL	Peptidyl-prolyl cis-trans isomerase 7 OS=Caenorhabditis elegans GN=cyn-7 PE=1 SV=2	18.4	8.7	3	451.1 (M:451.1)	11	57.9	2.35	30
31	SKPO1_CAEEL	Peroxidase skpo-1 OS=Caenorhabditis elegans GN=skpo-1 PE=2 SV=1	73.4	8.8	1	450.8 (M:450.8)	10	21.1	4.44	31
32	GBLP_CAEEL	Guanine nucleotide-binding protein subunit beta-2-like 1 OS=Caenorhabditis elegans GN=rack-1 PE=1 SV=3	35.8	6.4	1	447.0 (M:447.0)	9	37.8	4.26	32
33	HSP7F_CAEEL	Heat shock 70 kDa protein F, mitochondrial OS=Caenorhabditis elegans GN=hsp-6 PE=1 SV=2	70.8	5.9	1	429.2 (M:429.2)	8	18.0	6.62	33
34	KARG1_CAEEL	Probable arginine kinase F46H5.3 OS=Caenorhabditis elegans GN=F46H5.3 PE=1 SV=2	44.1	6.8	1	427.4 (M:427.4)	9	31.8	3.59	34
35	IFB1_CAEEL	Intermediate filament protein ifb-1 OS=Caenorhabditis elegans GN=ifb-1 PE=1 SV=1	67.1	5.9	2	423.3 (M:423.3)	13	25.5	3.00	35
36	VDAC_CAEEL	Probable voltage-dependent anion-selective channel OS=Caenorhabditis elegans GN=vdac-1 PE=3 SV=2	29.9	9.2	1	408.9 (M:408.9)	11	42.8	2.82	36
37	RSSA_CAEEL	40S ribosomal protein SA OS=Caenorhabditis elegans GN=rps-0 PE=1 SV=3	30.7	5.5	1	401.1 (M:401.1)	10	40.9	5.78	37
38	FABP2_CAEEL	Fatty acid-binding protein homolog 2 OS=Caenorhabditis elegans GN=lbp-2 PE=1 SV=1	18.8	6.2	1	394.0 (M:394.0)	10	46.0	4.04	38
39	SIP1_CAEEL	Stress-induced protein 1 OS=Caenorhabditis elegans GN=sip-1 PE=1 SV=1	17.8	7.9	1	389.1 (M:389.1)	10	49.1	3.34	39
40	ATPB_CAEEL	ATP synthase subunit beta, mitochondrial OS=Caenorhabditis elegans GN=atp-2 PE=1 SV=2	57.5	5.5	1	376.0 (M:376.0)	8	21.7	2.70	40
41	YZ10_CAEEL	Uncharacterized protein F08B12.4 OS=Caenorhabditis elegans GN=F08B12.4 PE=4 SV=2	10.3	6.4	1	368.4 (M:368.4)	12	88.2	5.46	41
42	TTR2_CAEEL	Transthyretin-like protein 2 OS=Caenorhabditis elegans GN=ttr-2 PE=1 SV=1	15.7	8.3	2	365.2 (M:365.2)	7	52.7	3.33	42
43	TBB4_CAEEL	Tubulin beta-4 chain OS=Caenorhabditis elegans GN=tbb-4 PE=3 SV=1	49.8	4.8	1	348.7 (M:348.7)	0	0.0	5.23	43
44	TERA1_CAEEL	Transitional endoplasmic reticulum ATPase homolog 1 OS=Caenorhabditis elegans GN=cdc-48.1 PE=1 SV=1	89.7	5.2	1	330.0 (M:330.0)	11	17.9	6.71	44
45	ENO_CAEEL	Enolase OS=Caenorhabditis elegans GN=enol-1 PE=1 SV=3	46.6	5.5	1	315.7 (M:315.7)	8	26.5	3.00	45
46	CHITL_CAEEL	Chitinase-like protein C25A8.4 OS=Caenorhabditis elegans GN=cht-3 PE=1 SV=3	120.1	8.9	1	312.9 (M:312.9)	9	11.0	2.46	46
47	SYDC_CAEEL	Aspartate--tRNA ligase, cytoplasmic OS=Caenorhabditis elegans GN=drs-1 PE=3 SV=1	59.9	6.0	1	310.6 (M:310.6)	8	20.5	4.32	47
48	FABP1_CAEEL	Fatty acid-binding protein homolog 1 OS=Caenorhabditis elegans GN=lbp-1 PE=1 SV=1	18.3	8.5	1	309.8 (M:309.8)	2	10.7	3.04	48
49	HSP7C_CAEEL	Heat shock 70 kDa protein C OS=Caenorhabditis elegans GN=hsp-3 PE=1 SV=2	73.0	5.0	1	296.8 (M:296.8)	1	3.0	3.22	49
50	RL4_CAEEL	60S ribosomal protein L4 OS=Caenorhabditis elegans GN=rpl-4 PE=1 SV=3	38.6	11.2	1	288.6 (M:288.6)	7	26.4	3.25	50
51	RLA1_CAEEL	60S acidic ribosomal protein P1 OS=Caenorhabditis elegans GN=rla-1 PE=3 SV=2	11.3	4.2	1	279.3 (M:279.3)	4	45.9	6.67	51
52	FAR2_CAEEL	Fatty-acid and retinol-binding protein 2 OS=Caenorhabditis elegans GN=far-2 PE=1 SV=1	20.0	5.7	1	278.8 (M:278.8)	7	43.4	2.80	52
53	MDHM_CAEEL	Probable malate dehydrogenase, mitochondrial OS=Caenorhabditis elegans GN=mdh-2 PE=3 SV=1	35.1	9.4	1	276.9 (M:276.9)	10	34.6	2.98	53
54	LMN1_CAEEL	Lamin-1 OS=Caenorhabditis elegans GN=lmn-1 PE=1 SV=2	64.0	5.4	1	275.3 (M:275.3)	5	14.5	4.41	54
55	PDI2_CAEEL	Protein disulfide-isomerase 2 OS=Caenorhabditis elegans GN=pdi-2 PE=1 SV=1	55.1	4.7	1	264.4 (M:264.4)	8	18.1	2.95	55
56	YUW5_CAEEL	Uncharacterized serine carboxypeptidase F41C3.5 OS=Caenorhabditis elegans GN=F41C3.5 PE=1 SV=1	53.6	6.2	1	261.3 (M:261.3)	7	15.4	3.42	56
57	RS8_CAEEL	40S ribosomal protein S8 OS=Caenorhabditis elegans GN=rps-8 PE=3 SV=1	23.7	10.6	1	261.1 (M:261.1)	6	31.2	4.13	57
58	G3P1_CAEEL	Glyceraldehyde-3-phosphate dehydrogenase 1 OS=Caenorhabditis elegans GN=gpd-1 PE=1 SV=1	36.4	7.7	2	260.3 (M:260.3)	8	33.4	2.63	58
59	IPYR_CAEEL	Probable inorganic pyrophosphatase 1 OS=Caenorhabditis elegans GN=pyp-1 PE=3 SV=4	46.4	5.8	1	259.7 (M:259.7)	5	19.9	3.21	59
60	CYP3_CAEEL	Peptidyl-prolyl cis-trans isomerase 3 OS=Caenorhabditis elegans GN=cyn-3 PE=1 SV=1	18.5	8.9	1	258.6 (M:258.6)	1	6.9	0.67	60
61	RLA2_CAEEL	60S acidic ribosomal protein P2 OS=Caenorhabditis elegans GN=rpa-2 PE=3 SV=2	10.8	4.5	1	254.8 (M:254.8)	4	51.4	3.44	61
62	TTR16_CAEEL	Transthyretin-like protein 16 OS=Caenorhabditis elegans GN=ttr-16 PE=1 SV=2	14.7	5.2	1	253.8 (M:253.8)	8	54.5	3.75	62
63	UNC87_CAEEL	Protein unc-87 OS=Caenorhabditis elegans GN=unc-87 PE=1 SV=3	62.7	9.1	1	251.0 (M:251.0)	7	17.0	3.07	63
64	LEC1_CAEEL	32 kDa beta-galactoside-binding lectin OS=Caenorhabditis elegans GN=lec-1 PE=1 SV=1	31.8	6.1	1	249.3 (M:249.3)	7	22.2	3.43	64
65	FABP9_CAEEL	Fatty acid-binding protein homolog 9 OS=Caenorhabditis elegans GN=lbp-9 PE=3 SV=1	16.9	7.6	1	249.1 (M:249.1)	5	43.4	4.49	65
66	RS2_CAEEL	40S ribosomal protein S2 OS=Caenorhabditis elegans GN=rps-2 PE=3 SV=1	28.9	10.1	1	246.7 (M:246.7)	7	28.3	2.87	66
67	RLA0_CAEEL	60S acidic ribosomal protein P0 OS=Caenorhabditis elegans GN=rpa-0 PE=1 SV=3	33.8	6.3	1	242.3 (M:242.3)	7	24.0	5.21	67
68	RS3_CAEEL	40S ribosomal protein S3 OS=Caenorhabditis elegans GN=rps-3 PE=3 SV=1	27.3	9.6	1	235.6 (M:235.6)	6	31.2	3.00	68
69	RL7_CAEEL	60S ribosomal protein L7 OS=Caenorhabditis elegans GN=rpl-7 PE=3 SV=1	28.1	10.2	1	230.9 (M:230.9)	6	29.1	3.88	69
70	MYO1_CAEEL	Myosin-1 OS=Caenorhabditis elegans GN=let-75 PE=1 SV=3	223.2	5.8	1	228.0 (M:228.0)	1	0.5	0.70	70
71	PCNA_CAEEL	Proliferating cell nuclear antigen OS=Caenorhabditis elegans GN=pcn-1 PE=1 SV=3	29.0	4.4	1	227.9 (M:227.9)	6	18.6	6.79	71
72	MYO2_CAEEL	Myosin-2 OS=Caenorhabditis elegans GN=myo-2 PE=1 SV=2	222.9	6.0	1	218.9 (M:218.9)	3	1.9	6.48	72

73	EF1B2_CAEEL	Probable elongation factor 1-beta/1-delta 2 OS=Caenorhabditis elegans GN=eef-1B.2 PE=1 SV=4	28.2	4.9	1	215.2 (M:215.2)	5	28.9	3.15	73
74	TERA2_CAEEL	Transitional endoplasmic reticulum ATPase homolog 2 OS=Caenorhabditis elegans GN=cdc-48.2 PE=1 SV=2	89.6	5.3	1	213.0 (M:213.0)	1	2.3	13.14	74
75	CALR_CAEEL	Calreticulin OS=Caenorhabditis elegans GN=crt-1 PE=1 SV=1	45.6	4.6	1	213.0 (M:213.0)	7	31.4	4.50	75
76	TPM1_CAEEL	Tropomyosin isoforms a/b/d/f OS=Caenorhabditis elegans GN=lev-11 PE=1 SV=1	33.0	4.7	2	212.4 (M:212.4)	6	25.0	3.89	76
77	H2B3_CAEEL	Probable histone H2B 3 OS=Caenorhabditis elegans GN=his-41 PE=3 SV=3	13.5	10.3	4	209.2 (M:209.2)	6	35.0	3.08	77
78	MPCP_CAEEL	Phosphate carrier protein, mitochondrial OS=Caenorhabditis elegans GN=F01G4.6 PE=2 SV=1	36.6	9.1	1	207.0 (M:207.0)	5	16.2	7.42	78
79	BTF3_CAEEL	Transcription factor BTF3 homolog OS=Caenorhabditis elegans GN=icd-1 PE=1 SV=1	17.5	8.7	1	205.0 (M:205.0)	4	26.7	6.14	79
80	CATA2_CAEEL	Catalase-2 OS=Caenorhabditis elegans GN=ctl-1 PE=2 SV=3	57.3	6.4	1	200.4 (M:200.4)	5	12.1	5.22	80
81	EF1B1_CAEEL	Probable elongation factor 1-beta/1-delta 1 OS=Caenorhabditis elegans GN=eef-1B.1 PE=1 SV=1	22.7	4.4	1	199.5 (M:199.5)	1	6.1	6.00	81
82	U375E_CAEEL	UPF0375 protein Y45F10C.2 OS=Caenorhabditis elegans GN=Y45F10C.2 PE=3 SV=1	12.8	4.9	2	197.8 (M:197.8)	6	59.8	3.79	82
83	RS4_CAEEL	40S ribosomal protein S4 OS=Caenorhabditis elegans GN=rps-4 PE=1 SV=1	29.0	10.5	1	195.1 (M:195.1)	6	24.7	3.32	83
84	G3P2_CAEEL	Glyceraldehyde-3-phosphate dehydrogenase 2 OS=Caenorhabditis elegans GN=gpd-2 PE=3 SV=2	36.4	6.8	2	194.4 (M:194.4)	4	16.1	4.91	84
85	YSX1_CAEEL	Uncharacterized protein T28D9.1 OS=Caenorhabditis elegans GN=T28D9.1 PE=4 SV=2	13.7	4.0	1	193.4 (M:193.4)	4	46.5	7.98	85
86	DLDH_CAEEL	Dihydrolipoyl dehydrogenase, mitochondrial OS=Caenorhabditis elegans GN=dld-1 PE=3 SV=2	52.6	7.6	1	191.5 (M:191.5)	4	13.3	6.67	86
87	RL19_CAEEL	60S ribosomal protein L19 OS=Caenorhabditis elegans GN=rpl-19 PE=3 SV=1	23.6	11.4	1	182.8 (M:182.8)	5	12.6	6.60	87
88	RS3A_CAEEL	40S ribosomal protein S3a OS=Caenorhabditis elegans GN=rps-1 PE=3 SV=2	28.9	9.6	1	179.4 (M:179.4)	7	28.8	3.36	88
89	RS12_CAEEL	40S ribosomal protein S12 OS=Caenorhabditis elegans GN=rps-12 PE=1 SV=2	15.1	6.2	1	179.1 (M:179.1)	4	30.7	7.46	89
90	RS14_CAEEL	40S ribosomal protein S14 OS=Caenorhabditis elegans GN=rps-14 PE=3 SV=1	16.2	10.4	1	176.3 (M:176.3)	4	36.2	7.71	90
91	FUMH_CAEEL	Probable fumarate hydratase, mitochondrial OS=Caenorhabditis elegans GN=fum-1 PE=1 SV=1	53.6	7.8	1	176.0 (M:176.0)	5	13.4	4.65	91
92	RS7_CAEEL	40S ribosomal protein S7 OS=Caenorhabditis elegans GN=rps-7 PE=3 SV=1	22.0	9.9	1	175.5 (M:175.5)	4	25.8	3.48	92
93	HSP11_CAEEL	Heat shock protein Hsp-16.1/Hsp-16.11 OS=Caenorhabditis elegans GN=hsp-16.1 PE=3 SV=1	16.2	5.4	1	172.9 (M:172.9)	4	42.1	2.76	93
94	RL7A_CAEEL	60S ribosomal protein L7a OS=Caenorhabditis elegans GN=rpl-7A PE=1 SV=3	30.2	10.8	1	172.2 (M:172.2)	5	20.8	2.87	94
95	HSP12_CAEEL	Heat shock protein Hsp-16.2 OS=Caenorhabditis elegans GN=hsp-16.2 PE=3 SV=1	16.2	5.3	1	167.8 (M:167.8)	1	10.3	3.42	95
96	ETFA_CAEEL	Probable electron transfer flavoprotein subunit alpha, mitochondrial OS=Caenorhabditis elegans GN=F27D4.1 PE=3 SV=2	34.4	9.2	1	166.6 (M:166.6)	3	16.3	9.06	96
97	YLA4_CAEEL	Uncharacterized protein C30C11.4 OS=Caenorhabditis elegans GN=C30C11.4 PE=1 SV=1	86.8	5.3	1	166.0 (M:166.0)	7	13.4	5.74	97
98	H4_CAEEL	Histone H4 OS=Caenorhabditis elegans GN=his-1 PE=1 SV=2	11.4	11.2	1	165.6 (M:165.6)	4	40.8	2.86	98
99	RL5_CAEEL	60S ribosomal protein L5 OS=Caenorhabditis elegans GN=rpl-5 PE=3 SV=1	33.4	9.8	1	165.5 (M:165.5)	4	16.4	4.86	99
100	CISY_CAEEL	Probable citrate synthase, mitochondrial OS=Caenorhabditis elegans GN=cts-1 PE=1 SV=1	51.5	7.7	1	165.0 (M:165.0)	5	15.2	11.42	100
101	IFA2_CAEEL	Intermediate filament protein ifa-2 OS=Caenorhabditis elegans GN=ifa-2 PE=1 SV=1	67.1	5.9	2	163.0 (M:163.0)	4	8.1	3.62	101
102	MLE_CAEEL	Myosin, essential light chain OS=Caenorhabditis elegans GN=mlc-3 PE=1 SV=1	17.1	4.6	1	160.3 (M:160.3)	4	31.4	5.45	102
103	SYV_CAEEL	Valine--tRNA ligase OS=Caenorhabditis elegans GN=vrs-2 PE=1 SV=1	118.8	6.3	1	159.4 (M:159.4)	3	4.3	2.36	103
104	TCPQ_CAEEL	T-complex protein 1 subunit theta OS=Caenorhabditis elegans GN=cct-8 PE=1 SV=3	59.7	5.8	1	156.4 (M:156.4)	5	11.9	4.09	104
105	EIF3A_CAEEL	Eukaryotic translation initiation factor 3 subunit A OS=Caenorhabditis elegans GN=egl-45 PE=3 SV=1	124.3	9.1	1	156.3 (M:156.3)	3	2.8	10.12	105
106	TBH1_CAEEL	Tyramine beta-hydroxylase OS=Caenorhabditis elegans GN=tbh-1 PE=1 SV=2	66.7	6.2	1	153.7 (M:153.7)	5	9.9	8.03	106
107	OAT_CAEEL	Probable ornithine aminotransferase, mitochondrial OS=Caenorhabditis elegans GN=C16A3.10 PE=3 SV=3	46.4	8.7	1	152.3 (M:152.3)	3	10.2	2.99	107
108	ASP6_CAEEL	Aspartic protease 6 OS=Caenorhabditis elegans GN=asp-6 PE=3 SV=1	41.5	5.6	1	149.9 (M:149.9)	3	13.6	3.27	108
109	HEXA_CAEEL	Beta-hexosaminidase A OS=Caenorhabditis elegans GN=hex-1 PE=1 SV=1	64.3	5.4	1	149.7 (M:149.7)	4	10.8	8.95	109
110	RL6_CAEEL	60S ribosomal protein L6 OS=Caenorhabditis elegans GN=rpl-6 PE=1 SV=1	24.3	11.0	1	148.3 (M:148.3)	4	20.7	3.15	110
111	CYC21_CAEEL	Cytochrome c 2.1 OS=Caenorhabditis elegans GN=cyc-2.1 PE=1 SV=2	12.2	9.9	1	143.6 (M:143.6)	4	40.5	2.94	111
112	MYO3_CAEEL	Myosin-3 OS=Caenorhabditis elegans GN=myo-3 PE=2 SV=1	225.4	5.5	1	141.3 (M:141.3)	3	2.0	10.78	112
113	KARG2_CAEEL	Probable arginine kinase ZC434.8 OS=Caenorhabditis elegans GN=ZC434.8 PE=3 SV=1	40.4	9.3	1	139.7 (M:139.7)	3	11.7	9.37	113
114	RS5_CAEEL	40S ribosomal protein S5 OS=Caenorhabditis elegans GN=rps-5 PE=3 SV=1	23.1	9.8	1	137.1 (M:137.1)	4	23.8	1.76	114
115	ADF1_CAEEL	Actin-depolymerizing factor 1, isoforms a/b OS=Caenorhabditis elegans GN=unc-60 PE=1 SV=2	23.6	5.6	1	136.5 (M:136.5)	4	26.9	6.47	115
116	RL8_CAEEL	60S ribosomal protein L8 OS=Caenorhabditis elegans GN=rpl-8 PE=3 SV=1	28.2	11.1	1	136.4 (M:136.4)	3	20.0	2.76	116
117	RL18_CAEEL	60S ribosomal protein L18 OS=Caenorhabditis elegans GN=rpl-18 PE=3 SV=1	21.0	11.4	1	132.3 (M:132.3)	3	17.6	3.03	117
118	RS6_CAEEL	40S ribosomal protein S6 OS=Caenorhabditis elegans GN=rps-6 PE=1 SV=1	28.1	10.8	1	131.2 (M:131.2)	4	17.5	3.22	118
119	PSMD3_CAEEL	26S proteasome non-ATPase regulatory subunit 3 OS=Caenorhabditis elegans GN=rpn-3 PE=1 SV=1	57.5	7.1	1	130.2 (M:130.2)	3	9.1	6.18	119
120	TTR15_CAEEL	Transthyretin-like protein 15 OS=Caenorhabditis elegans GN=ttr-15 PE=3 SV=1	14.5	7.6	1	130.0 (M:130.0)	4	36.2	2.99	120
121	CALM_CAEEL	Calmodulin OS=Caenorhabditis elegans GN=cmd-1 PE=1 SV=3	16.8	4.1	1	128.7 (M:128.7)	5	40.3	6.45	121
122	ATPD_CAEEL	ATP synthase subunit delta, mitochondrial OS=Caenorhabditis elegans GN=F58F12.1 PE=1 SV=1	16.9	4.8	1	126.6 (M:126.6)	3	21.5	3.61	122
123	CAV1_CAEEL	Caveolin-1 OS=Caenorhabditis elegans GN=cav-1 PE=2 SV=1	26.3	5.3	1	126.1 (M:126.1)	3	23.0	7.50	123
124	PROF1_CAEEL	Profilin-1 OS=Caenorhabditis elegans GN=pfn-1 PE=2 SV=1	14.2	5.2	1	125.5 (M:125.5)	4	34.1	5.18	124
125	CRI3_CAEEL	Conserved regulator of innate immunity protein 3 OS=Caenorhabditis elegans GN=cri-3 PE=1 SV=2	26.4	4.9	1	123.1 (M:123.1)	3	23.7	2.11	125
126	CHIT_CAEEL	Probable endochitinase OS=Caenorhabditis elegans GN=cht-1 PE=1 SV=1	66.8	8.0	1	120.6 (M:120.6)	4	10.5	9.62	126
127	PSMD1_CAEEL	26S proteasome non-ATPase regulatory subunit 1 OS=Caenorhabditis elegans GN=rpn-2 PE=3 SV=4	105.9	7.5	1	120.1 (M:120.1)	5	5.0	6.53	127
128	U376B_CAEEL	UPF0376 protein C17B7.2 OS=Caenorhabditis elegans GN=C17B7.2 PE=1 SV=1	38.9	5.6	1	118.0 (M:118.0)	3	13.4	6.15	128
129	HSP17_CAEEL	Heat shock protein Hsp-16.48/Hsp-16.49 OS=Caenorhabditis elegans GN=hsp-16.48 PE=2 SV=1	16.3	5.5	1	116.9 (M:116.9)	2	25.2	2.79	129
130	RL22_CAEEL	60S ribosomal protein L22 OS=Caenorhabditis elegans GN=rpl-22 PE=1 SV=3	14.9	9.8	1	116.7 (M:116.7)	3	23.8	4.84	130
131	FABP6_CAEEL	Fatty acid-binding protein homolog 6 OS=Caenorhabditis elegans GN=lbp-6 PE=1 SV=1	15.6	6.8	1	116.2 (M:116.2)	3	25.9	4.79	131
132	RL40_CAEEL	Ubiquitin-60S ribosomal protein L40 OS=Caenorhabditis elegans GN=ubq-2 PE=3 SV=2	14.6	10.6	2	116.0 (M:116.0)	3	26.6	3.57	132
133	AN322_CAEEL	Acidic leucine-rich nuclear phosphoprotein 32-related protein 2 OS=Caenorhabditis elegans GN=T19H12.2 PE=3 SV=1	24.4	4.1	1	115.8 (M:115.8)	3	20.4	2.52	133
134	FAR1_CAEEL	Fatty-acid and retinol-binding protein 1 OS=Caenorhabditis elegans GN=far-1 PE=3 SV=1	20.1	7.0	1	113.7 (M:113.7)	3	18.1	3.67	134
135	YZVL_CAEEL	Uncharacterized NOP5 family protein K07C5.4 OS=Caenorhabditis elegans GN=K07C5.4 PE=3 SV=1	54.5	8.7	1	113.5 (M:113.5)	4	13.4	3.00	135
136	CGH1_CAEEL	ATP-dependent RNA helicase cgh-1 OS=Caenorhabditis elegans GN=cgh-1 PE=1 SV=1	48.7	8.3	1	111.7 (M:111.7)	4	8.8	1.75	136
137	PSA1_CAEEL	Proteasome subunit alpha type-1 OS=Caenorhabditis elegans GN=pas-6 PE=1 SV=1	28.3	6.5	1	111.5 (M:111.5)	2	13.8	6.00	137
138	ACBP1_CAEEL	Acyl-CoA-binding protein homolog 1 OS=Caenorhabditis elegans GN=acbp-1 PE=3 SV=1	9.4	6.2	1	111.2 (M:111.2)	3	46.5	4.14	138
139	SYSC_CAEEL	Probable serine--tRNA ligase, cytoplasmic OS=Caenorhabditis elegans GN=srs-2 PE=3 SV=1	55.2	5.9	1	110.6 (M:110.6)	3	8.6	9.01	139
140	RL9_CAEEL	60S ribosomal protein L9 OS=Caenorhabditis elegans GN=rpl-9 PE=3 SV=1	21.5	10.1	1	108.7 (M:108.7)	4	32.8	1.53	140
141	RL10A_CAEEL	60S ribosomal protein L10a OS=Caenorhabditis elegans GN=rpl-10a PE=3 SV=1	24.1	9.9	1	108.5 (M:108.5)	3	11.6	2.22	141
142	GSTP1_CAEEL	Glutathione S-transferase P OS=Caenorhabditis elegans GN=gst-1 PE=1 SV=1	23.9	5.9	1	106.9 (M:106.9)	2	13.5	3.38	142
143	TTR5_CAEEL	Transthyretin-like protein 5 OS=Caenorhabditis elegans GN=ttr-5 PE=3 SV=1	15.0	5.9	1	106.7 (M:106.7)	2	23.7	4.83	143
144	RL12_CAEEL	60S ribosomal protein L12 OS=Caenorhabditis elegans GN=rpl-12 PE=3 SV=1	17.8	9.5	1	106.2 (M:106.2)	2	17.0	3.83	144
145	LMP1_CAEEL	LAMP family protein lmp-1 OS=Caenorhabditis elegans GN=lmp-1 PE=2 SV=1	25.8	5.2	1	105.2 (M:105.2)	2	11.0	3.08	145
146	DIM_CAEEL	Disorganized muscle protein 1 OS=Caenorhabditis elegans GN=dim-1 PE=1 SV=3	71.8	8.1	1	104.8 (M:104.8)	3	7.0	12.30	146
147	VATA_CAEEL	V-type proton ATPase catalytic subunit A OS=Caenorhabditis elegans GN=vha-13 PE=1 SV=3	66.4	5.1	1	103.4 (M:103.4)	4	12.2	3.98	147
148	RS19_CAEEL	40S ribosomal protein S19 OS=Caenorhabditis elegans GN=rps-19 PE=2 SV=1	16.3	10.3	1	102.3 (M:102.3)	3	21.2	3.37	148
149	HSP16_CAEEL	Heat shock protein Hsp-16.41 OS=Caenorhabditis elegans GN=hsp-16.41 PE=2 SV=1	16.2	5.9	1	102.2 (M:102.2)	1	9.8	0.68	149

150	DCTN2_CAEEL	Probable dynactin subunit 2 OS=Caenorhabditis elegans GN=dnc-2 PE=1 SV=1	37.1	5.1	1	101.1 (M:101.1)	2	9.1	3.89	150
151	RS28_CAEEL	40S ribosomal protein S28 OS=Caenorhabditis elegans GN=rps-28 PE=3 SV=1	7.4	10.7	1	101.0 (M:101.0)	2	35.4	2.44	151
152	YH24_CAEEL	Putative aminopeptidase W07G4.4 OS=Caenorhabditis elegans GN=lap-2 PE=1 SV=1	56.1	6.6	1	100.9 (M:100.9)	2	5.4	2.65	152
153	YSD2_CAEEL	RutC family protein C23G10.2 OS=Caenorhabditis elegans GN=C23G10.2 PE=3 SV=3	18.0	5.5	1	99.6 (M:99.6)	2	14.6	5.23	153
154	RL24_CAEEL	60S ribosomal protein L24 OS=Caenorhabditis elegans GN=rpl-24.1 PE=3 SV=1	17.8	11.3	1	99.2 (M:99.2)	3	17.0	3.90	154
155	RL17_CAEEL	60S ribosomal protein L17 OS=Caenorhabditis elegans GN=rpl-17 PE=3 SV=1	21.5	10.3	1	98.9 (M:98.9)	3	12.3	4.28	155
156	RL13_CAEEL	60S ribosomal protein L13 OS=Caenorhabditis elegans GN=rpl-13 PE=3 SV=1	23.7	11.1	1	98.8 (M:98.8)	2	12.6	3.42	156
157	MCCB_CAEEL	Probable methylcrotonoyl-CoA carboxylase beta chain, mitochondrial OS=Caenorhabditis elegans GN=F02A9.4 PE=3 SV=1	66.5	8.6	1	97.4 (M:97.4)	2	5.9	11.88	157
158	U375F_CAEEL	UPF0375 protein Y45F10C.4 OS=Caenorhabditis elegans GN=Y45F10C.4 PE=1 SV=1	13.5	5.4	1	97.1 (M:97.1)	2	18.5	6.26	158
159	PGK_CAEEL	Probable phosphoglycerate kinase OS=Caenorhabditis elegans GN=pgk-1 PE=1 SV=1	44.1	6.5	1	94.9 (M:94.9)	5	13.9	2.80	159
160	RL15_CAEEL	60S ribosomal protein L15 OS=Caenorhabditis elegans GN=rpl-15 PE=3 SV=1	24.1	11.6	1	93.1 (M:93.1)	2	6.9	4.11	160
161	RS25_CAEEL	40S ribosomal protein S25 OS=Caenorhabditis elegans GN=rps-25 PE=3 SV=1	12.9	10.1	1	92.1 (M:92.1)	2	20.5	3.25	161
162	RS21_CAEEL	40S ribosomal protein S21 OS=Caenorhabditis elegans GN=rps-21 PE=1 SV=1	9.7	6.5	1	91.9 (M:91.9)	2	31.8	7.63	162
163	ODP2_CAEEL	Dihydrolipoyllysine-residue acetyltransferase component of pyruvate dehydrogenase complex, mitochondrial OS=Caenorhabditis elegans GN=F23B12.5 PE=1 SV=1	53.4	8.4	1	91.8 (M:91.8)	2	4.7	5.20	163
164	PDIA6_CAEEL	Probable protein disulfide-isomerase A6 OS=Caenorhabditis elegans GN=tag-320 PE=3 SV=1	47.7	5.8	1	91.5 (M:91.5)	2	9.8	7.37	164
165	HEH1_CAEEL	Putative protein heh-1 OS=Caenorhabditis elegans GN=heh-1 PE=3 SV=2	16.6	8.2	1	90.4 (M:90.4)	3	20.1	8.17	165
166	IFA1_CAEEL	Intermediate filament protein ifa-1 OS=Caenorhabditis elegans GN=ifa-1 PE=1 SV=2	66.5	6.1	1	89.5 (M:89.5)	2	3.5	3.95	166
167	TPIS_CAEEL	Triosephosphate isomerase OS=Caenorhabditis elegans GN=tpi-1 PE=1 SV=2	26.6	6.2	1	89.2 (M:89.2)	2	11.7	3.06	167
168	UBP14_CAEEL	Ubiquitin carboxyl-terminal hydrolase 14 OS=Caenorhabditis elegans GN=usp-14 PE=2 SV=2	55.8	5.4	1	88.8 (M:88.8)	2	6.1	6.66	168
169	BAF1_CAEEL	Barrier-to-autointegration factor 1 OS=Caenorhabditis elegans GN=baf-1 PE=1 SV=3	9.9	5.0	1	87.9 (M:87.9)	2	28.1	10.77	169
170	RL3_CAEEL	60S ribosomal protein L3 OS=Caenorhabditis elegans GN=rpl-3 PE=2 SV=1	45.6	10.4	1	87.9 (M:87.9)	2	6.7	5.32	170
171	MLRH_CAEEL	Probable myosin regulatory light chain OS=Caenorhabditis elegans GN=mlc-4 PE=3 SV=1	19.9	4.6	1	87.3 (M:87.3)	2	12.8	4.37	171
172	EF1G_CAEEL	Probable elongation factor 1-gamma OS=Caenorhabditis elegans GN=eef-1G PE=3 SV=1	44.4	6.3	1	87.1 (M:87.1)	2	4.8	2.87	172
173	RHO1_CAEEL	Ras-like GTP-binding protein rhoA OS=Caenorhabditis elegans GN=rho-1 PE=1 SV=1	21.6	6.0	1	86.7 (M:86.7)	2	8.9	9.50	173
174	PSA7_CAEEL	Proteasome subunit alpha type-7 OS=Caenorhabditis elegans GN=pas-4 PE=1 SV=1	28.2	5.9	1	86.6 (M:86.6)	3	16.2	6.72	174
175	FBRL_CAEEL	rRNA 2'-O-methyltransferase fibrillarin OS=Caenorhabditis elegans GN=fib-1 PE=3 SV=1	36.4	10.3	1	86.3 (M:86.3)	2	9.7	4.61	175
176	SYQ_CAEEL	Probable glutamine--tRNA ligase OS=Caenorhabditis elegans GN=ers-1 PE=3 SV=1	88.2	7.6	1	86.1 (M:86.1)	2	3.9	1.09	176
177	PSA5_CAEEL	Proteasome subunit alpha type-5 OS=Caenorhabditis elegans GN=pas-5 PE=1 SV=1	27.2	5.3	1	86.0 (M:86.0)	4	16.9	6.93	177
178	SYMC_CAEEL	Methionine--tRNA ligase, cytoplasmic OS=Caenorhabditis elegans GN=mrs-1 PE=3 SV=1	101.6	8.0	1	85.9 (M:85.9)	2	3.2	9.41	178
179	RL18A_CAEEL	60S ribosomal protein L18a OS=Caenorhabditis elegans GN=rpl-20 PE=3 SV=2	20.9	10.4	1	85.2 (M:85.2)	2	15.0	4.18	179
180	SYRC_CAEEL	Probable arginine--tRNA ligase, cytoplasmic OS=Caenorhabditis elegans GN=rrt-1 PE=3 SV=2	80.8	7.8	1	85.0 (M:85.0)	2	3.8	13.04	180
181	GPDH2_CAEEL	Probable glycerol-3-phosphate dehydrogenase 2 OS=Caenorhabditis elegans GN=gpdh-2 PE=3 SV=2	42.8	6.3	1	84.8 (M:84.8)	2	8.2	6.06	181
182	YC4B5_CAEEL	Uncharacterized protein C44B7.5 OS=Caenorhabditis elegans GN=C44B7.5 PE=1 SV=1	26.3	8.4	1	83.6 (M:83.6)	4	14.0	4.16	182
183	VATH2_CAEEL	Probable V-type proton ATPase subunit H 2 OS=Caenorhabditis elegans GN=vha-15 PE=3 SV=1	54.2	5.9	1	83.6 (M:83.6)	3	8.9	4.95	183
184	RFA1_CAEEL	Probable replication factor A 73 kDa subunit OS=Caenorhabditis elegans GN=rpa-1 PE=1 SV=1	73.2	5.7	1	80.9 (M:80.9)	1	3.4	4.93	184
185	IMA2_CAEEL	Importin subunit alpha-2 OS=Caenorhabditis elegans GN=ima-2 PE=1 SV=1	59.1	5.0	1	80.5 (M:80.5)	2	6.6	9.51	185
186	COX6A_CAEEL	Probable cytochrome c oxidase subunit 6A, mitochondrial OS=Caenorhabditis elegans GN=tag-174 PE=3 SV=1	14.7	9.6	1	80.2 (M:80.2)	2	18.8	6.02	186
187	IFA4_CAEEL	Intermediate filament protein ifa-4 OS=Caenorhabditis elegans GN=ifa-4 PE=1 SV=3	66.3	6.2	1	79.2 (M:79.2)	2	4.7	9.41	187
188	NACA_CAEEL	Nascent polypeptide-associated complex subunit alpha OS=Caenorhabditis elegans GN=Y65B4BR.5 PE=1 SV=1	22.1	5.0	1	79.0 (M:79.0)	2	12.2	11.80	188
189	PSB1_CAEEL	Proteasome subunit beta type-1 OS=Caenorhabditis elegans GN=pbs-6 PE=1 SV=2	28.9	5.6	1	78.4 (M:78.4)	2	9.7	3.17	189
190	ARF12_CAEEL	ADP-ribosylation factor 1-like 2 OS=Caenorhabditis elegans GN=arf-1.2 PE=2 SV=2	20.5	6.2	1	78.3 (M:78.3)	2	19.3	2.82	190
191	TCPE_CAEEL	T-complex protein 1 subunit epsilon OS=Caenorhabditis elegans GN=cct-5 PE=1 SV=1	59.4	5.4	1	78.0 (M:78.0)	2	4.1	3.84	191
192	RIR1_CAEEL	Ribonucleoside-diphosphate reductase large subunit OS=Caenorhabditis elegans GN=rnr-1 PE=3 SV=1	88.9	7.0	1	77.5 (M:77.5)	3	4.7	5.70	192
193	RS17_CAEEL	40S ribosomal protein S17 OS=Caenorhabditis elegans GN=rps-17 PE=3 SV=2	14.9	9.8	1	76.6 (M:76.6)	2	16.2	3.94	193
194	TCPB_CAEEL	T-complex protein 1 subunit beta OS=Caenorhabditis elegans GN=cct-2 PE=1 SV=2	56.9	5.7	1	75.9 (M:75.9)	3	7.9	3.42	194
195	EIF3B_CAEEL	Eukaryotic translation initiation factor 3 subunit B OS=Caenorhabditis elegans GN=eif-3.B PE=3 SV=2	83.0	5.7	1	75.2 (M:75.2)	2	4.4	6.39	195
196	PSA6_CAEEL	Proteasome subunit alpha type-6 OS=Caenorhabditis elegans GN=pas-1 PE=1 SV=1	27.0	8.2	1	73.7 (M:73.7)	3	12.6	4.06	196
197	UCR1_CAEEL	Cytochrome b-c1 complex subunit 1, mitochondrial OS=Caenorhabditis elegans GN=ucr-1 PE=3 SV=2	51.7	6.1	1	73.5 (M:73.5)	1	3.2	1.51	197
198	CPG1_CAEEL	Chondroitin proteoglycan 1 OS=Caenorhabditis elegans GN=cpg-1 PE=1 SV=1	61.8	3.7	1	72.6 (M:72.6)	1	3.9	3.75	198
199	PHB2_CAEEL	Mitochondrial prohibitin complex protein 2 OS=Caenorhabditis elegans GN=phb-2 PE=1 SV=2	32.6	9.7	1	72.2 (M:72.2)	2	7.1	2.62	199
200	YOQ7_CAEEL	Uncharacterized protein ZK512.7 OS=Caenorhabditis elegans GN=ZK512.7 PE=4 SV=1	16.0	6.7	1	71.9 (M:71.9)	2	10.6	13.11	200
201	RAN_CAEEL	GTP-binding nuclear protein ran-1 OS=Caenorhabditis elegans GN=ran-1 PE=1 SV=1	24.2	7.0	1	71.5 (M:71.5)	2	10.7	4.71	201
202	SODM1_CAEEL	Superoxide dismutase [Mn] 1, mitochondrial OS=Caenorhabditis elegans GN=sod-2 PE=1 SV=1	24.5	7.8	2	71.2 (M:71.2)	2	9.5	2.96	202
203	AN321_CAEEL	Acidic leucine-rich nuclear phosphoprotein 32-related protein 1 OS=Caenorhabditis elegans GN=F33H2.3 PE=3 SV=1	24.8	4.0	1	71.2 (M:71.2)	1	9.2	11.40	203
204	PLBL1_CAEEL	Putative phospholipase B-like 1 OS=Caenorhabditis elegans GN=Y37D8A.2 PE=1 SV=1	65.4	6.6	1	70.9 (M:70.9)	2	3.7	5.45	204
205	CLC87_CAEEL	C-type lectin domain-containing protein 87 OS=Caenorhabditis elegans GN=clec-87 PE=1 SV=1	26.7	5.2	1	70.8 (M:70.8)	2	11.2	10.60	205
206	GLYC_CAEEL	Serine hydroxymethyltransferase OS=Caenorhabditis elegans GN=mel-32 PE=1 SV=2	55.7	8.7	1	70.3 (M:70.3)	3	8.1	3.99	206
207	IF4A_CAEEL	Eukaryotic initiation factor 4A OS=Caenorhabditis elegans GN=inf-1 PE=2 SV=1	45.4	5.0	1	70.1 (M:70.1)	3	8.2	2.56	207
208	RS26_CAEEL	40S ribosomal protein S26 OS=Caenorhabditis elegans GN=rps-26 PE=3 SV=1	13.2	11.1	1	70.0 (M:70.0)	2	20.5	3.22	208
209	RL11_CAEEL	60S ribosomal protein L11 OS=Caenorhabditis elegans GN=rpl-11.1 PE=3 SV=1	22.7	10.0	1	69.8 (M:69.8)	3	10.7	2.47	209
210	PCCA_CAEEL	Propionyl-CoA carboxylase alpha chain, mitochondrial OS=Caenorhabditis elegans GN=pcca-1 PE=1 SV=1	79.7	7.6	1	68.9 (M:68.9)	3	5.7	2.42	210
211	OLA1_CAEEL	Obg-like ATPase 1 OS=Caenorhabditis elegans GN=ola-1 PE=3 SV=1	44.3	6.4	1	68.4 (M:68.4)	2	5.3	9.55	211
212	ODPA_CAEEL	Probable pyruvate dehydrogenase E1 component subunit alpha, mitochondrial OS=Caenorhabditis elegans GN=T05H10.6 PE=3 SV=1	43.8	7.1	1	67.9 (M:67.9)	1	3.3	4.35	212
213	TIM50_CAEEL	Mitochondrial import inner membrane translocase subunit TIM50 OS=Caenorhabditis elegans GN=scpl-4 PE=3 SV=1	51.3	6.7	1	67.8 (M:67.8)	1	5.1	4.62	213
214	SODC_CAEEL	Superoxide dismutase [Cu-Zn] OS=Caenorhabditis elegans GN=sod-1 PE=1 SV=2	18.7	6.1	1	67.4 (M:67.4)	1	9.4	2.63	214
215	RS13_CAEEL	40S ribosomal protein S13 OS=Caenorhabditis elegans GN=rps-13 PE=3 SV=2	17.3	10.7	1	67.3 (M:67.3)	3	25.8	2.33	215
216	RS16_CAEEL	40S ribosomal protein S16 OS=Caenorhabditis elegans GN=rps-16 PE=1 SV=3	16.3	10.3	1	63.2 (M:63.2)	3	17.4	2.87	216
217	RS23_CAEEL	40S ribosomal protein S23 OS=Caenorhabditis elegans GN=rps-23 PE=3 SV=1	15.9	10.5	1	63.2 (M:63.2)	1	7.7	2.31	217
218	RL26_CAEEL	60S ribosomal protein L26 OS=Caenorhabditis elegans GN=rpl-26 PE=3 SV=1	16.1	11.3	1	62.9 (M:62.9)	2	14.1	3.49	218
219	COPD_CAEEL	Probable coatomer subunit delta OS=Caenorhabditis elegans GN=C13B9.3 PE=3 SV=1	56.6	5.7	1	62.3 (M:62.3)	2	5.8	3.70	219
220	TNNT_CAEEL	Troponin T OS=Caenorhabditis elegans GN=mup-2 PE=2 SV=1	47.0	4.8	1	61.9 (M:61.9)	2	4.2	3.77	220
221	MLR2_CAEEL	Myosin regulatory light chain 2 OS=Caenorhabditis elegans GN=mlc-2 PE=1 SV=1	18.6	5.1	2	61.4 (M:61.4)	2	16.5	2.26	221
222	FRDA_CAEEL	Frataxin, mitochondrial OS=Caenorhabditis elegans GN=frh-1 PE=2 SV=1	15.7	5.3	1	61.2 (M:61.2)	1	12.5	4.73	222
223	STT3_CAEEL	Dolichyl-diphosphooligosaccharide--protein glycosyltransferase subunit STT3 OS=Caenorhabditis elegans GN=T12A2.2 PE=1 SV=1	85.1	8.9	1	60.5 (M:60.5)	1	2.0	1.43	223
224	UGDH_CAEEL	UDP-glucose 6-dehydrogenase OS=Caenorhabditis elegans GN=sqv-4 PE=1 SV=1	52.7	5.9	1	59.7 (M:59.7)	2	5.8	3.76	224
225	LDH_CAEEL	L-lactate dehydrogenase OS=Caenorhabditis elegans GN=ldh-1 PE=2 SV=1	36.0	6.4	1	59.5 (M:59.5)	2	6.6	3.98	225
226	VATG_CAEEL	Probable V-type proton ATPase subunit G OS=Caenorhabditis elegans GN=vha-10 PE=3 SV=1	14.5	9.6	1	58.7 (M:58.7)	2	15.9	4.04	226

227	UBP7_CAEEL	Ubiquitin carboxyl-terminal hydrolase 7 OS=Caenorhabditis elegans GN=usp-7 PE=3 SV=1	131.5	5.4	1	58.6 (M:58.6)	1	1.3	4.12	227
228	FABP3_CAEEL	Fatty acid-binding protein homolog 3 OS=Caenorhabditis elegans GN=lbp-3 PE=2 SV=2	19.1	8.4	2	57.9 (M:57.9)	3	20.0	3.82	228
229	CLC91_CAEEL	C-type lectin domain-containing protein 91 OS=Caenorhabditis elegans GN=clec-91 PE=1 SV=1	25.6	5.4	1	57.5 (M:57.5)	1	8.9	4.29	229
230	CCHL_CAEEL	Probable cytochrome c-type heme lyase OS=Caenorhabditis elegans GN=cchl-1 PE=3 SV=1	31.2	6.2	1	57.2 (M:57.2)	1	7.1	5.00	230
231	CPG8_CAEEL	Chondroitin proteoglycan 8 OS=Caenorhabditis elegans GN=cpg-8 PE=1 SV=2	12.2	4.2	1	56.8 (M:56.8)	1	13.9	2.26	231
232	RS9_CAEEL	40S ribosomal protein S9 OS=Caenorhabditis elegans GN=rps-9 PE=3 SV=1	21.9	10.5	1	56.6 (M:56.6)	2	9.0	4.93	232
233	GLH1_CAEEL	ATP-dependent RNA helicase glh-1 OS=Caenorhabditis elegans GN=glh-1 PE=1 SV=3	79.7	5.6	2	56.4 (M:56.4)	2	3.8	10.97	233
234	ROA1_CAEEL	Heterogeneous nuclear ribonucleoprotein A1 OS=Caenorhabditis elegans GN=hrp-1 PE=1 SV=1	36.3	8.4	1	56.3 (M:56.3)	1	4.6	11.88	234
235	IF5A1_CAEEL	Eukaryotic translation initiation factor 5A-1 OS=Caenorhabditis elegans GN=iff-1 PE=1 SV=1	17.9	5.1	1	55.9 (M:55.9)	2	20.5	4.93	235
236	NXT1_CAEEL	NTF2-related export protein OS=Caenorhabditis elegans GN=nxt-1 PE=1 SV=1	15.5	4.9	1	55.8 (M:55.8)	1	12.4	5.08	236
237	YOCA_CAEEL	Uncharacterized protein ZC395.10 OS=Caenorhabditis elegans GN=ZC395.10 PE=3 SV=1	19.4	4.3	1	55.2 (M:55.2)	2	16.6	6.34	237
238	SYB1_CAEEL	Synaptobrevin-1 OS=Caenorhabditis elegans GN=snb-1 PE=1 SV=1	12.0	9.2	1	55.0 (M:55.0)	1	11.9	3.86	238
239	NEPL1_CAEEL	Neprilysin-1 OS=Caenorhabditis elegans GN=nep-1 PE=1 SV=3	86.9	5.7	1	54.9 (M:54.9)	1	2.0	7.08	239
240	YOD2_CAEEL	Uncharacterized protein ZC262.2 OS=Caenorhabditis elegans GN=ZC262.2 PE=4 SV=1	26.8	9.9	1	54.7 (M:54.7)	1	10.7	0.13	240
241	IMDH_CAEEL	Inosine-5'-monophosphate dehydrogenase OS=Caenorhabditis elegans GN=T22D1.3 PE=3 SV=2	58.1	7.0	1	54.4 (M:54.4)	1	2.1	4.84	241
242	YP93_CAEEL	Uncharacterized helicase C28H8.3 OS=Caenorhabditis elegans GN=C28H8.3 PE=3 SV=2	194.0	6.3	1	52.9 (M:52.9)	1	1.2	5.47	242
243	RS15_CAEEL	40S ribosomal protein S15 OS=Caenorhabditis elegans GN=rps-15 PE=1 SV=3	17.2	10.3	1	52.2 (M:52.2)	1	7.9	2.58	243
244	RS27_CAEEL	40S ribosomal protein S27 OS=Caenorhabditis elegans GN=rps-27 PE=1 SV=3	9.3	9.4	1	52.0 (M:52.0)	2	25.3	2.70	244
245	STIP1_CAEEL	Stress-induced-phosphoprotein 1 OS=Caenorhabditis elegans GN=sti-1 PE=1 SV=1	36.9	6.6	1	50.9 (M:50.9)	1	3.1	4.25	245
246	EPI1_CAEEL	Laminin-like protein epi-1 OS=Caenorhabditis elegans GN=epi-1 PE=1 SV=1	404.0	5.2	1	50.2 (M:50.2)	2	1.0	14.29	246
247	TOP2_CAEEL	Probable DNA topoisomerase 2 OS=Caenorhabditis elegans GN=K12D12.1 PE=3 SV=2	172.2	8.6	1	49.7 (M:49.7)	1	0.7	4.16	247
248	YKA3_CAEEL	Uncharacterized protein B0303.3 OS=Caenorhabditis elegans GN=B0303.3 PE=3 SV=1	47.8	9.1	1	49.1 (M:49.1)	2	5.6	1.79	248
249	YWV3_CAEEL	Uncharacterized protein B0403.3 OS=Caenorhabditis elegans GN=B0403.3 PE=3 SV=2	31.8	5.9	1	49.0 (M:49.0)	2	7.4	4.70	249
250	TDX1_CAEEL	Probable peroxiredoxin prdx-3 OS=Caenorhabditis elegans GN=prdx-3 PE=1 SV=1	24.9	6.9	1	48.9 (M:48.9)	1	4.4	6.79	250
251	AT1B1_CAEEL	Sodium/potassium-transporting ATPase subunit beta-1 OS=Caenorhabditis elegans GN=nkb-1 PE=1 SV=1	36.6	7.5	1	48.8 (M:48.8)	1	6.6	2.84	251
252	SYTC_CAEEL	Threonine--tRNA ligase, cytoplasmic OS=Caenorhabditis elegans GN=trs-1 PE=3 SV=1	84.4	7.1	1	48.7 (M:48.7)	2	2.9	7.80	252
253	RPAB3_CAEEL	Probable DNA-directed RNA polymerases I, II, and III subunit RPABC3 OS=Caenorhabditis elegans GN=rpb-8 PE=1 SV=1	17.0	4.9	1	48.4 (M:48.4)	1	8.1	4.91	253
254	IDH3A_CAEEL	Probable isocitrate dehydrogenase [NAD] subunit alpha, mitochondrial OS=Caenorhabditis elegans GN=idha-1 PE=1 SV=3	38.4	7.0	1	48.0 (M:48.0)	2	8.1	4.08	254
255	RSMB_CAEEL	Probable small nuclear ribonucleoprotein-associated protein B OS=Caenorhabditis elegans GN=snr-2 PE=3 SV=1	16.7	10.0	1	47.6 (M:47.6)	1	6.9	0.86	255
256	PSA3_CAEEL	Proteasome subunit alpha type-3 OS=Caenorhabditis elegans GN=pas-7 PE=1 SV=3	27.7	5.0	1	47.2 (M:47.2)	1	5.6	3.93	256
257	ERH_CAEEL	Enhancer of rudimentary homolog OS=Caenorhabditis elegans GN=T21C9.4 PE=1 SV=1	12.3	5.1	1	47.1 (M:47.1)	1	16.5	11.95	257
258	YQ83_CAEEL	GYF domain-containing protein C18H9.3 OS=Caenorhabditis elegans GN=C18H9.3/C18H9.2 PE=3 SV=3	113.4	9.1	1	45.7 (M:45.7)	3	5.4	4.69	258
259	AMPL_CAEEL	Leucine aminopeptidase 1 OS=Caenorhabditis elegans GN=lap-1 PE=1 SV=1	52.4	6.2	1	45.5 (M:45.5)	1	2.4	4.63	259
260	RL35_CAEEL	60S ribosomal protein L35 OS=Caenorhabditis elegans GN=rpl-35 PE=3 SV=1	14.2	11.3	1	45.4 (M:45.4)	1	8.1	2.20	260
261	RL31_CAEEL	60S ribosomal protein L31 OS=Caenorhabditis elegans GN=rpl-31 PE=3 SV=1	14.3	10.1	1	44.7 (M:44.7)	2	15.6	2.65	261
262	MCM6_CAEEL	DNA replication licensing factor mcm-6 OS=Caenorhabditis elegans GN=mcm-6 PE=1 SV=1	91.1	5.2	1	44.6 (M:44.6)	2	3.5	4.03	262
263	PP2C2_CAEEL	Probable protein phosphatase 2C T23F11.1 OS=Caenorhabditis elegans GN=ppm-2 PE=3 SV=2	39.0	4.5	1	44.1 (M:44.1)	1	3.7	6.03	263
264	DIG1_CAEEL	Mesocentin OS=Caenorhabditis elegans GN=dig-1 PE=1 SV=2	1372.5	4.1	1	43.8 (M:43.8)	1	0.1	13.29	264
265	TCPD_CAEEL	T-complex protein 1 subunit delta OS=Caenorhabditis elegans GN=cct-4 PE=2 SV=1	58.4	6.1	1	43.6 (M:43.6)	2	6.9	2.66	265
266	MTSS1_CAEEL	Single-stranded DNA-binding protein, mitochondrial OS=Caenorhabditis elegans GN=mtss-1 PE=2 SV=2	19.2	9.8	1	43.5 (M:43.5)	1	7.1	8.64	266
267	DHX15_CAEEL	Putative pre-mRNA-splicing factor ATP-dependent RNA helicase F56D2.6 OS=Caenorhabditis elegans GN=F56D2.6 PE=3 SV=1	84.3	8.3	1	42.7 (M:42.7)	1	2.0	0.22	267
268	RL27_CAEEL	60S ribosomal protein L27 OS=Caenorhabditis elegans GN=rpl-27 PE=2 SV=1	15.7	10.8	1	42.6 (M:42.6)	1	6.6	4.21	268
269	PSA2_CAEEL	Proteasome subunit alpha type-2 OS=Caenorhabditis elegans GN=pas-2 PE=1 SV=1	25.3	5.7	1	42.3 (M:42.3)	1	8.7	4.69	269
270	GCP2_CAEEL	Glutamate carboxypeptidase 2 homolog OS=Caenorhabditis elegans GN=R57.1 PE=1 SV=2	85.4	7.4	1	42.2 (M:42.2)	1	1.8	4.04	270
271	ZPR1_CAEEL	Zinc finger protein ZPR1 homolog OS=Caenorhabditis elegans GN=W03F9.1 PE=3 SV=2	50.9	4.6	1	41.9 (M:41.9)	1	4.2	8.62	271
272	TIM9_CAEEL	Mitochondrial import inner membrane translocase subunit Tim9 OS=Caenorhabditis elegans GN=tin-9.1 PE=3 SV=1	10.2	7.7	1	41.8 (M:41.8)	1	12.2	12.56	272
273	RCC1_CAEEL	Regulator of chromosome condensation OS=Caenorhabditis elegans GN=ran-3 PE=3 SV=1	61.4	5.3	1	41.2 (M:41.2)	1	2.5	6.94	273
274	NSF_CAEEL	Vesicle-fusing ATPase OS=Caenorhabditis elegans GN=nsf-1 PE=1 SV=2	91.3	7.3	1	41.1 (M:41.1)	1	1.8	5.44	274
275	RL28_CAEEL	60S ribosomal protein L28 OS=Caenorhabditis elegans GN=rpl-28 PE=1 SV=3	13.7	11.7	1	40.2 (M:40.2)	2	12.7	4.51	275
276	GABT_CAEEL	Probable 4-aminobutyrate aminotransferase, mitochondrial OS=Caenorhabditis elegans GN=gta-1 PE=1 SV=1	53.0	8.7	1	40.1 (M:40.1)	2	4.8	5.15	276
277	RSP3_CAEEL	Probable splicing factor, arginine/serine-rich 3 OS=Caenorhabditis elegans GN=rsp-3 PE=1 SV=2	28.7	10.3	1	40.0 (M:40.0)	1	4.7	5.66	277
278	EIF2A_CAEEL	Eukaryotic translation initiation factor 2A OS=Caenorhabditis elegans GN=E04D5.1 PE=3 SV=2	64.0	9.2	1	39.9 (M:39.9)	1	3.0	5.07	278
279	RIR2_CAEEL	Ribonucleoside-diphosphate reductase small chain OS=Caenorhabditis elegans GN=rnr-2 PE=3 SV=1	44.3	4.8	1	39.1 (M:39.1)	1	2.9	2.37	279
280	EMR1_CAEEL	Emerin homolog 1 OS=Caenorhabditis elegans GN=emr-1 PE=1 SV=1	18.1	4.6	1	39.0 (M:39.0)	1	6.6	9.19	280
281	STIM1_CAEEL	Stromal interaction molecule 1 OS=Caenorhabditis elegans GN=stim-1 PE=1 SV=1	60.0	6.3	1	39.0 (M:39.0)	1	2.8	3.55	281
282	PRP8_CAEEL	Pre-mRNA-splicing factor 8 homolog OS=Caenorhabditis elegans GN=prp-8 PE=1 SV=1	271.9	8.9	1	38.5 (M:38.5)	1	0.5	1.60	282
283	H11_CAEEL	Histone H1.1 OS=Caenorhabditis elegans GN=his-24 PE=1 SV=4	21.4	10.9	1	37.9 (M:37.9)	1	6.2	2.74	283
284	YVRI_CAEEL	GILT-like protein F37H8.5 OS=Caenorhabditis elegans GN=F37H8.5 PE=1 SV=1	30.8	6.9	1	37.6 (M:37.6)	2	6.1	2.80	284
285	TMA7_CAEEL	Translation machinery-associated protein 7 homolog OS=Caenorhabditis elegans GN=F49C12.11 PE=3 SV=1	6.9	10.0	1	37.2 (M:37.2)	1	15.6	8.78	285
286	MGN_CAEEL	Protein mago nashi homolog OS=Caenorhabditis elegans GN=mag-1 PE=1 SV=2	17.6	5.1	1	36.9 (M:36.9)	1	8.6	0.94	286
287	AL7A1_CAEEL	Putative aldehyde dehydrogenase family 7 member A1 homolog OS=Caenorhabditis elegans GN=alh-9 PE=3 SV=2	57.0	6.3	1	36.7 (M:36.7)	1	2.3	3.03	287
288	MMSA_CAEEL	Probable methylmalonate-semialdehyde dehydrogenase [acylating], mitochondrial OS=Caenorhabditis elegans GN=alh-8 PE=1 SV=1	56.4	7.6	1	36.1 (M:36.1)	1	2.3	2.14	288
289	BRE1_CAEEL	E3 ubiquitin-protein ligase bre-1 OS=Caenorhabditis elegans GN=rfp-1 PE=1 SV=2	97.4	6.6	1	35.7 (M:35.7)	1	1.9	13.82	289
290	COX2_CAEEL	Cytochrome c oxidase subunit 2 OS=Caenorhabditis elegans GN=cox-2 PE=3 SV=2	26.5	4.8	1	35.6 (M:35.6)	1	4.8	3.98	290
291	RL23_CAEEL	60S ribosomal protein L23 OS=Caenorhabditis elegans GN=rpl-23 PE=3 SV=1	14.9	10.5	1	35.5 (M:35.5)	2	11.4	7.13	291
292	PAR1_CAEEL	Serine/threonine-protein kinase par-1 OS=Caenorhabditis elegans GN=par-1 PE=1 SV=1	126.3	9.5	1	35.4 (M:35.4)	1	1.6	10.86	292
293	PROF2_CAEEL	Profilin-2 OS=Caenorhabditis elegans GN=pfn-2 PE=2 SV=3	14.2	5.4	1	35.2 (M:35.2)	1	6.1	9.55	293
294	CSK2A_CAEEL	Casein kinase II subunit alpha OS=Caenorhabditis elegans GN=kin-3 PE=1 SV=1	42.2	6.4	1	35.1 (M:35.1)	1	6.7	3.69	294
295	SMD3_CAEEL	Small nuclear ribonucleoprotein Sm D3 OS=Caenorhabditis elegans GN=snr-1 PE=2 SV=2	14.8	10.8	1	34.8 (M:34.8)	1	14.7	9.02	295
296	WDR12_CAEEL	Ribosome biogenesis protein WDR12 homolog OS=Caenorhabditis elegans GN=tag-345 PE=3 SV=1	49.0	5.0	1	34.7 (M:34.7)	1	3.0	4.91	296
297	LARP1_CAEEL	La-related protein 1 OS=Caenorhabditis elegans GN=larp-1 PE=3 SV=2	128.2	9.0	1	34.4 (M:34.4)	2	2.6	2.48	297
298	SPCS2_CAEEL	Probable signal peptidase complex subunit 2 OS=Caenorhabditis elegans GN=Y37D8A.10 PE=1 SV=1	20.6	5.8	1	33.6 (M:33.6)	1	6.7	6.42	298
299	SYFB_CAEEL	Phenylalanine--tRNA ligase beta subunit OS=Caenorhabditis elegans GN=frs-2 PE=1 SV=2	66.0	5.7	1	33.3 (M:33.3)	1	2.0	5.54	299
300	H2A_CAEEL	Histone H2A OS=Caenorhabditis elegans GN=his-3 PE=1 SV=2	13.4	10.7	2	33.2 (M:33.2)	2	12.6	2.72	300
301	PCP5_CAEEL	Prolyl carboxy peptidase like protein 5 OS=Caenorhabditis elegans GN=pcp-5 PE=1 SV=1	56.9	6.0	1	33.1 (M:33.1)	1	2.2	3.67	301
302	CYP5_CAEEL	Peptidyl-prolyl cis-trans isomerase 5 OS=Caenorhabditis elegans GN=cyn-5 PE=1 SV=2	21.9	9.0	2	33.0 (M:33.0)	2	7.8	6.63	302
303	GPX2_CAEEL	Glutathione peroxidase 2 OS=Caenorhabditis elegans GN=gpx-2 PE=3 SV=1	18.1	6.7	1	32.9 (M:32.9)	1	9.2	3.88	303

304	VATF_CAEEL	Probable V-type proton ATPase subunit F OS=Caenorhabditis elegans GN=vha-9 PE=3 SV=1	13.3	5.0	1	32.0 (M:32.0)	1	14.0	15.53	304
305	CBP1_CAEEL	Protein cbp-1 OS=Caenorhabditis elegans GN=cbp-1 PE=3 SV=6	222.3	8.6	1	31.0 (M:31.0)	1	0.8	2.17	305
306	TPP2_CAEEL	Tripeptidyl-peptidase 2 OS=Caenorhabditis elegans GN=tpp-2 PE=2 SV=1	151.0	7.7	1	30.5 (M:30.5)	1	0.9	4.59	306
307	NTF2_CAEEL	Probable nuclear transport factor 2 OS=Caenorhabditis elegans GN=ran-4 PE=3 SV=1	15.1	4.8	1	30.3 (M:30.3)	1	17.3	10.85	307
308	PSB3_CAEEL	Proteasome subunit beta type-3 OS=Caenorhabditis elegans GN=pbs-3 PE=1 SV=1	22.7	5.3	1	29.8 (M:29.8)	1	6.4	1.59	308
309	6PGD_CAEEL	6-phosphogluconate dehydrogenase, decarboxylating OS=Caenorhabditis elegans GN=T25B9.9 PE=3 SV=2	53.2	6.4	1	29.6 (M:29.6)	1	2.3	10.22	309
310	EIF3C_CAEEL	Eukaryotic translation initiation factor 3 subunit C OS=Caenorhabditis elegans GN=eif-3.C PE=3 SV=2	103.8	5.8	1	29.5 (M:29.5)	2	2.4	13.29	310
311	SLBP_CAEEL	Histone RNA hairpin-binding protein OS=Caenorhabditis elegans GN=cdl-1 PE=2 SV=1	41.5	9.4	1	29.5 (M:29.5)	1	4.1	6.91	311
312	DRE2_CAEEL	Anamorsin homolog OS=Caenorhabditis elegans GN=T20B12.7 PE=3 SV=1	25.5	4.6	1	29.5 (M:29.5)	1	4.6	0.15	312
313	NDUS7_CAEEL	Probable NADH dehydrogenase [ubiquinone] iron-sulfur protein 7, mitochondrial OS=Caenorhabditis elegans GN=nduf-7 PE=3 SV=1	21.9	9.6	1	29.3 (M:29.3)	1	7.0	5.48	313
314	CPG2_CAEEL	Chondroitin proteoglycan-2 OS=Caenorhabditis elegans GN=cpg-2 PE=1 SV=3	53.6	3.8	1	29.0 (M:29.0)	1	1.5	3.99	314
315	SUCB2_CAEEL	Probable succinyl-CoA ligase [GDP-forming] subunit beta, mitochondrial OS=Caenorhabditis elegans GN=C50F7.4 PE=3 SV=1	45.1	5.6	1	28.9 (M:28.9)	1	4.8	10.47	315
316	NAS37_CAEEL	Zinc metalloproteinase nas-37 OS=Caenorhabditis elegans GN=nas-37 PE=1 SV=2	85.1	8.7	1	28.4 (M:28.4)	1	2.0	3.61	316
317	GMPR_CAEEL	Probable GMP reductase OS=Caenorhabditis elegans GN=F32D1.5 PE=3 SV=1	38.8	7.2	1	28.3 (M:28.3)	1	2.5	3.86	317
318	YC2BA_CAEEL	EGF-like domain-containing protein C02B10.3 OS=Caenorhabditis elegans GN=C02B10.3 PE=1 SV=2	27.3	7.4	1	28.2 (M:28.2)	1	4.9	11.08	318
319	PA2L_CAEEL	Phospholipase A2-like protein Y52B11A.8 OS=Caenorhabditis elegans GN=Y52B11A.8 PE=1 SV=1	18.8	5.1	1	28.1 (M:28.1)	1	8.6	6.03	319
320	LE767_CAEEL	Very-long-chain 3-oxooacyl-coA reductase let-767 OS=Caenorhabditis elegans GN=let-767 PE=1 SV=2	34.3	9.5	3	27.5 (M:27.5)	1	5.4	0.14	320
321	ACADM_CAEEL	Probable medium-chain specific acyl-CoA dehydrogenase 10, mitochondrial OS=Caenorhabditis elegans GN=acdh-10 PE=2 SV=1	44.8	8.4	1	27.0 (M:27.0)	1	1.9	4.87	321
322	PEBPH_CAEEL	Phosphatidylethanolamine-binding protein homolog F40A3.3 OS=Caenorhabditis elegans GN=F40A3.3 PE=3 SV=1	24.1	8.7	1	26.6 (M:26.6)	1	8.6	12.53	322
323	ALF1_CAEEL	Fructose-bisphosphate aldolase 1 OS=Caenorhabditis elegans GN=aldo-1 PE=1 SV=1	39.2	6.3	1	26.3 (M:26.3)	1	3.0	0.85	323
324	SYAC_CAEEL	Alanine--tRNA ligase, cytoplasmic OS=Caenorhabditis elegans GN=aars-2 PE=2 SV=1	106.7	5.5	1	26.1 (M:26.1)	2	1.7	11.83	324
325	CSN2_CAEEL	COP9 signalosome complex subunit 2 OS=Caenorhabditis elegans GN=csn-2 PE=1 SV=2	56.7	5.3	1	25.1 (M:25.1)	1	1.8	6.75	325
326	YZG1_CAEEL	Uncharacterized protein F13E6.1 OS=Caenorhabditis elegans GN=F13E6.1 PE=3 SV=2	21.3	5.6	1	25.0 (M:25.0)	1	8.7	3.93	326
327	DYL1_CAEEL	Dynein light chain 1, cytoplasmic OS=Caenorhabditis elegans GN=dlc-1 PE=1 SV=1	10.3	6.8	1	25.0 (M:25.0)	1	24.7	9.20	327
328	TNNI2_CAEEL	Troponin I 2 OS=Caenorhabditis elegans GN=unc-27 PE=2 SV=2	27.5	5.5	1	24.9 (M:24.9)	1	5.4	3.38	328
329	GCP_CAEEL	Bifunctional glyoxylate cycle protein OS=Caenorhabditis elegans GN=icl-1 PE=1 SV=2	108.6	6.4	1	24.4 (M:24.4)	1	1.0	3.86	329
330	G6PD_CAEEL	Glucose-6-phosphate 1-dehydrogenase OS=Caenorhabditis elegans GN=gspd-1 PE=3 SV=1	60.2	8.2	1	24.3 (M:24.3)	1	1.7	12.98	330
331	ANC1_CAEEL	Nuclear anchorage protein 1 OS=Caenorhabditis elegans GN=anc-1 PE=1 SV=3	955.9	4.7	1	24.3 (M:24.3)	1	0.1	4.29	331
332	SUP1_CAEEL	Protein SUP-1 OS=Caenorhabditis elegans GN=sup-1 PE=1 SV=1	11.3	7.5	1	24.3 (M:24.3)	1	13.6	4.07	332
333	METK1_CAEEL	Probable S-adenosylmethionine synthase 1 OS=Caenorhabditis elegans GN=sams-1 PE=1 SV=1	43.6	6.0	4	24.0 (M:24.0)	1	3.7	2.63	333
334	RL21_CAEEL	60S ribosomal protein L21 OS=Caenorhabditis elegans GN=rpl-21 PE=1 SV=3	18.3	11.1	1	24.0 (M:24.0)	1	11.8	2.89	334
335	FLP14_CAEEL	FMRFamide-like neuropeptides 14 OS=Caenorhabditis elegans GN=flp-14 PE=1 SV=2	15.9	8.5	1	23.5 (M:23.5)	1	14.0	15.23	335
336	YOT9_CAEEL	Uncharacterized protein ZK632.9 OS=Caenorhabditis elegans GN=ZK632.9 PE=4 SV=3	6.6	9.2	1	23.5 (M:23.5)	1	16.1	1.60	336
337	PSMD9_CAEEL	Probable 26S proteasome non-ATPase regulatory subunit 9 OS=Caenorhabditis elegans GN=psmd-9 PE=1 SV=2	22.1	5.5	1	23.0 (M:23.0)	1	8.1	3.28	337
338	KAD2_CAEEL	Adenylate kinase OS=Caenorhabditis elegans GN=let-754 PE=3 SV=2	27.9	9.0	1	22.5 (M:22.5)	1	4.4	3.65	338
339	RL44_CAEEL	60S ribosomal protein L44 OS=Caenorhabditis elegans GN=rpl-41 PE=3 SV=2	12.4	10.8	1	21.8 (M:21.8)	1	6.7	2.48	339
340	UBC2_CAEEL	Ubiquitin-conjugating enzyme E2 2 OS=Caenorhabditis elegans GN=let-70 PE=1 SV=1	16.7	6.8	1	21.7 (M:21.7)	1	8.8	1.02	340
341	PHB1_CAEEL	Mitochondrial prohibitin complex protein 1 OS=Caenorhabditis elegans GN=phb-1 PE=1 SV=1	30.0	6.9	1	21.7 (M:21.7)	1	3.3	9.22	341
342	YDJ1_CAEEL	Uncharacterized protein ZK1073.1 OS=Caenorhabditis elegans GN=ZK1073.1 PE=3 SV=1	35.6	6.9	1	21.3 (M:21.3)	1	5.8	2.35	342
343	YLZ2_CAEEL	Uncharacterized protein F42H10.2 OS=Caenorhabditis elegans GN=F42H10.2 PE=4 SV=1	12.7	4.3	1	21.1 (M:21.1)	1	12.2	3.66	343
344	CATA1_CAEEL	Peroxisomal catalase 1 OS=Caenorhabditis elegans GN=ctl-2 PE=2 SV=3	57.4	8.6	1	21.0 (M:21.0)	1	3.0	7.41	344
345	IF4E1_CAEEL	Eukaryotic translation initiation factor 4E-1 OS=Caenorhabditis elegans GN=ife-1 PE=1 SV=2	24.3	5.5	2	20.9 (M:20.9)	1	4.2	7.77	345
346	ADF2_CAEEL	Actin-depolymerizing factor 2, isoform c OS=Caenorhabditis elegans GN=unc-60 PE=1 SV=1	17.0	6.3	1	20.9 (M:20.9)	1	9.2	0.28	346
347	CED2_CAEEL	Cell death abnormality protein 2 OS=Caenorhabditis elegans GN=ced-2 PE=1 SV=1	30.9	5.8	1	20.7 (M:20.7)	1	5.7	3.24	347
348	TOM40_CAEEL	Mitochondrial import receptor subunit TOM40 homolog OS=Caenorhabditis elegans GN=C18E9.6 PE=3 SV=1	32.4	7.1	1	20.3 (M:20.3)	1	3.0	4.33	348
349	IF4E3_CAEEL	Eukaryotic translation initiation factor 4E-3 OS=Caenorhabditis elegans GN=ife-3 PE=1 SV=2	28.2	5.9	1	20.1 (M:20.1)	1	8.8	13.91	349
350	SDS22_CAEEL	Protein phosphatase 1 regulatory subunit SDS22 homolog OS=Caenorhabditis elegans GN=sds-22 PE=3 SV=1	37.3	4.9	1	20.0 (M:20.0)	1	3.7	0.82	350
351	SUCA_CAEEL	Probable succinyl-CoA ligase [ADP/GDP-forming] subunit alpha, mitochondrial OS=Caenorhabditis elegans GN=C05G5.4 PE=3 SV=1	33.8	9.2	1	19.9 (M:19.9)	1	2.8	1.42	351
352	SDHB_CAEEL	Succinate dehydrogenase [ubiquinone] iron-sulfur subunit, mitochondrial OS=Caenorhabditis elegans GN=sdhb-1 PE=2 SV=1	32.9	8.6	1	19.7 (M:19.7)	1	8.1	1.81	352
353	PXL1_CAEEL	Paxillin homolog 1 OS=Caenorhabditis elegans GN=pxl-1 PE=2 SV=2	46.4	8.2	1	18.8 (M:18.8)	1	3.6	4.50	353
354	MIC10_CAEEL	MICOS complex subunit Mic10 OS=Caenorhabditis elegans GN=F54A3.5 PE=3 SV=2	11.1	9.1	1	18.7 (M:18.7)	1	9.5	0.82	354
355	GDIR_CAEEL	Probable rho GDP-dissociation inhibitor OS=Caenorhabditis elegans GN=rhi-1 PE=3 SV=1	21.9	6.1	1	18.6 (M:18.6)	1	6.3	7.86	355
356	PGL1_CAEEL	P granule abnormality protein 1 OS=Caenorhabditis elegans GN=pgl-1 PE=1 SV=1	78.4	4.9	1	18.6 (M:18.6)	1	1.4	12.75	356
357	PAT3_CAEEL	Integrin beta pat-3 OS=Caenorhabditis elegans GN=pat-3 PE=1 SV=1	90.1	5.2	1	17.6 (M:17.6)	1	1.1	5.56	357
358	DAF19_CAEEL	RFX-like transcription factor daf-19 OS=Caenorhabditis elegans GN=daf-19 PE=2 SV=3	90.9	6.0	1	17.4 (M:17.4)	1	5.0	16.31	358
359	RL35A_CAEEL	60S ribosomal protein L35a OS=Caenorhabditis elegans GN=rpl-33 PE=1 SV=3	13.8	11.3	1	17.4 (M:17.4)	1	7.3	4.99	359
360	SF3B4_CAEEL	Splicing factor 3B subunit 4 OS=Caenorhabditis elegans GN=sap-49 PE=1 SV=2	41.0	7.1	1	17.1 (M:17.1)	1	3.6	8.03	360
361	DX39B_CAEEL	Spliceosome RNA helicase DDX39B homolog OS=Caenorhabditis elegans GN=hel-1 PE=2 SV=1	48.5	5.5	1	16.8 (M:16.8)	1	2.6	0.18	361
362	DSS1_CAEEL	Probable 26S proteasome complex subunit dss-1 OS=Caenorhabditis elegans GN=dss-1 PE=1 SV=2	9.5	4.3	1	16.6 (M:16.6)	1	22.0	12.87	362
363	NCS2_CAEEL	Neuronal calcium sensor 2 OS=Caenorhabditis elegans GN=ncs-2 PE=2 SV=2	22.0	5.1	1	16.6 (M:16.6)	1	5.8	0.88	363
364	EIF3G_CAEEL	Eukaryotic translation initiation factor 3 subunit G OS=Caenorhabditis elegans GN=eif-3.G PE=3 SV=2	29.9	5.9	1	16.5 (M:16.5)	1	3.4	2.55	364
365	YPP4_CAEEL	Uncharacterized protein F32A5.4 OS=Caenorhabditis elegans GN=F32A5.4 PE=1 SV=1	26.4	6.6	1	16.5 (M:16.5)	1	9.1	10.65	365
366	KC1A_CAEEL	Casein kinase I isoform alpha OS=Caenorhabditis elegans GN=kin-19 PE=3 SV=1	39.0	9.6	1	16.4 (M:16.4)	1	11.1	9.77	366
367	UN112_CAEEL	Protein unc-112 OS=Caenorhabditis elegans GN=unc-112 PE=1 SV=1	82.3	6.5	1	16.4 (M:16.4)	1	1.9	7.26	367
368	VATB_CAEEL	Probable V-type proton ATPase subunit B OS=Caenorhabditis elegans GN=vha-12 PE=1 SV=1	54.7	5.3	1	16.4 (M:16.4)	1	1.8	0.08	368
369	ATIF2_CAEEL	ATPase inhibitor mai-2, mitochondrial OS=Caenorhabditis elegans GN=mai-2 PE=3 SV=1	12.0	8.1	1	16.3 (M:16.3)	1	6.4	3.64	369
370	CWC22_CAEEL	Pre-mRNA-splicing factor CWC22 homolog OS=Caenorhabditis elegans GN=let-858 PE=2 SV=1	104.2	6.4	1	15.9 (M:15.9)	1	1.6	10.22	370
371	PURA_CAEEL	Adenylosuccinate synthetase OS=Caenorhabditis elegans GN=C37H5.6 PE=3 SV=3	50.1	6.8	1	15.8 (M:15.8)	1	3.7	1.46	371
372	UNC22_CAEEL	Twitchin OS=Caenorhabditis elegans GN=unc-22 PE=1 SV=3	788.5	5.8	1	15.3 (M:15.3)	1	0.2	0.93	372
373	PRS10_CAEEL	Probable 26S protease regulatory subunit 10B OS=Caenorhabditis elegans GN=rpt-4 PE=1 SV=2	45.8	7.6	1	15.2 (M:15.2)	1	3.0	12.52	373
374	SPT6H_CAEEL	Suppressor of Ty 6 homolog OS=Caenorhabditis elegans GN=emb-5 PE=1 SV=1	175.7	4.9	1	15.0 (M:15.0)	1	1.2	13.17	374
375	PRS6B_CAEEL	Probable 26S protease regulatory subunit 6B OS=Caenorhabditis elegans GN=rpt-3 PE=3 SV=1	46.3	5.1	1	14.3 (M:14.3)	1	2.2	2.71	375
376	GST7_CAEEL	Probable glutathione S-transferase 7 OS=Caenorhabditis elegans GN=gst-7 PE=3 SV=1	23.1	6.3	2	14.2 (M:14.2)	1	3.9	3.90	376
377	EXT2_CAEEL	Exostosin-2 OS=Caenorhabditis elegans GN=rib-2 PE=2 SV=2	94.1	6.0	1	14.0 (M:14.0)	1	5.5	6.95	377
378	SPD5_CAEEL	Spindle-defective protein 5 OS=Caenorhabditis elegans GN=spd-5 PE=1 SV=2	135.1	5.2	1	14.0 (M:14.0)	1	1.2	5.85	378
379	PRS7_CAEEL	26S protease regulatory subunit 7 OS=Caenorhabditis elegans GN=rpt-1 PE=1 SV=1	48.6	6.1	1	13.7 (M:13.7)	1	3.0	0.18	379
380	BRX1_CAEEL	Ribosome biogenesis protein BRX1 homolog OS=Caenorhabditis elegans GN=K12H4.3 PE=3 SV=1	40.2	9.1	1	13.5 (M:13.5)	1	4.8	3.82	380

381	LGG1_CAEEL	Protein lgg-1 OS=Caenorhabditis elegans GN=lgg-1 PE=1 SV=1	14.8	7.9	1	13.3 (M:13.3)	1	5.7	11.77	381
382	GLOD4_CAEEL	Glyoxalase 1 OS=Caenorhabditis elegans GN=glod-4 PE=2 SV=1	32.1	5.6	1	13.3 (M:13.3)	1	4.6	8.38	382
383	PSMD8_CAEEL	26S proteasome non-ATPase regulatory subunit 8 OS=Caenorhabditis elegans GN=rpn-12 PE=1 SV=1	28.8	5.6	1	13.1 (M:13.1)	1	4.4	1.58	383
384	CAPZA_CAEEL	F-actin-capping protein subunit alpha OS=Caenorhabditis elegans GN=cap-1 PE=2 SV=1	32.2	6.0	1	13.0 (M:13.0)	1	3.2	12.28	384

Supplementary Table 2. Proteins identified in 3-day-old worms
(In order of MASCOT score)

Row	User Flag	OK	Accession	Protein	MW [kDa]	pI	#Alt. Proteins	Scores	#Peptides	SC [%]	RMS90 [ppm]	Rank
1		true	VIT6_CAEEL	Vitellogenin-6 OS=Caenorhabditis elegans GN=vit-6 PE=1 SV=5	193.2	6.9	1	2937.4 (M:2937.4)	40	26.7	3.03	1
2		true	MYO4_CAEEL	Myosin-4 OS=Caenorhabditis elegans GN=unc-54 PE=1 SV=2	224.6	5.6	2	2271.3 (M:2271.3)	34	16.8	2.72	2
3		true	VIT2_CAEEL	Vitellogenin-2 OS=Caenorhabditis elegans GN=vit-2 PE=1 SV=5	187.6	6.2	1	1449.1 (M:1449.1)	29	19.2	1.76	3
4		true	VIT5_CAEEL	Vitellogenin-5 OS=Caenorhabditis elegans GN=vit-5 PE=2 SV=2	186.3	6.6	2	1172.4 (M:1172.4)	28	15.2	2.30	4
5		true	VIT1_CAEEL	Vitellogenin-1 OS=Caenorhabditis elegans GN=vit-1 PE=1 SV=2	187.9	6.5	1	1070.2 (M:1070.2)	1	1.7	7.42	5
6		true	HSP90_CAEEL	Heat shock protein 90 OS=Caenorhabditis elegans GN=daf-21 PE=1 SV=1	80.2	5.0	1	992.6 (M:992.6)	13	24.1	2.31	6
7		true	VIT3_CAEEL	Vitellogenin-3 OS=Caenorhabditis elegans GN=vit-3 PE=1 SV=1	186.4	6.5	1	985.7 (M:985.7)	3	1.7	3.20	7
8		true	RLA1_CAEEL	60S acidic ribosomal protein P1 OS=Caenorhabditis elegans GN=rla-1 PE=3 SV=2	11.3	4.2	1	793.8 (M:793.8)	7	45.9	2.55	8
9		true	ACT4_CAEEL	Actin-4 OS=Caenorhabditis elegans GN=act-4 PE=3 SV=2	41.8	5.2	3	639.5 (M:639.5)	9	26.6	2.40	9
10		true	EF2_CAEEL	Elongation factor 2 OS=Caenorhabditis elegans GN=eef-2 PE=1 SV=4	94.7	6.1	1	581.3 (M:581.3)	12	13.1	2.44	10
11		true	MYSP_CAEEL	Paramyosin OS=Caenorhabditis elegans GN=unc-15 PE=1 SV=1	101.9	5.3	1	576.4 (M:576.4)	12	15.5	2.21	11
12		true	CH60_CAEEL	Chaperonin homolog Hsp-60, mitochondrial OS=Caenorhabditis elegans GN=hsp-60 PE=1 SV=2	60.1	5.3	1	522.6 (M:522.6)	9	19.5	2.67	12
13		true	HSP7A_CAEEL	Heat shock 70 kDa protein A OS=Caenorhabditis elegans GN=hsp-1 PE=1 SV=2	69.7	5.4	1	476.5 (M:476.5)	11	25.0	1.95	13
14		true	CYP7_CAEEL	Peptidyl-prolyl cis-trans isomerase 7 OS=Caenorhabditis elegans GN=cyn-7 PE=1 SV=2	18.4	8.7	2	456.1 (M:456.1)	5	20.5	1.51	14
15		true	ATPA_CAEEL	ATP synthase subunit alpha, mitochondrial OS=Caenorhabditis elegans GN=H28O16.1 PE=1 SV=1	57.8	9.5	1	423.9 (M:423.9)	10	20.1	2.10	15
16		true	TCTP_CAEEL	Translationally-controlled tumor protein homolog OS=Caenorhabditis elegans GN=tct-1 PE=1 SV=1	20.5	4.8	1	414.8 (M:414.8)	5	24.9	3.42	16
17		true	TPM1_CAEEL	Tropomyosin isoforms a/b/d/f OS=Caenorhabditis elegans GN=lev-11 PE=1 SV=1	33.0	4.7	2	410.7 (M:410.7)	6	22.5	1.69	17
18		true	RSSA_CAEEL	40S ribosomal protein SA OS=Caenorhabditis elegans GN=rps-0 PE=1 SV=3	30.7	5.5	1	382.0 (M:382.0)	6	38.4	2.22	18
19		true	EF1G_CAEEL	Probable elongation factor 1-gamma OS=Caenorhabditis elegans GN=eef-1G PE=3 SV=1	44.4	6.3	1	373.8 (M:373.8)	8	20.1	2.66	19
20		true	HSP7F_CAEEL	Heat shock 70 kDa protein F, mitochondrial OS=Caenorhabditis elegans GN=hsp-6 PE=1 SV=2	70.8	5.9	1	365.5 (M:365.5)	4	7.8	2.13	20
21		true	RL7A_CAEEL	60S ribosomal protein L7a OS=Caenorhabditis elegans GN=rpl-7A PE=1 SV=3	30.2	10.8	1	360.6 (M:360.6)	5	21.1	3.79	21
22		true	YSX1_CAEEL	Uncharacterized protein T28D9.1 OS=Caenorhabditis elegans GN=T28D9.1 PE=4 SV=2	13.7	4.0	1	343.3 (M:343.3)	4	41.9	1.34	22
23		true	MLE_CAEEL	Myosin, essential light chain OS=Caenorhabditis elegans GN=mlc-3 PE=1 SV=1	17.1	4.6	1	343.0 (M:343.0)	4	28.8	2.66	23
24		true	ACON_CAEEL	Probable aconitate hydratase, mitochondrial OS=Caenorhabditis elegans GN=aco-2 PE=1 SV=2	84.0	8.2	1	342.3 (M:342.3)	3	5.3	9.99	24
25		true	SAHH_CAEEL	Adenosylhomocysteinase OS=Caenorhabditis elegans GN=ahcy-1 PE=1 SV=1	47.5	5.9	1	331.2 (M:331.2)	4	10.1	3.64	25
26		true	RS7_CAEEL	40S ribosomal protein S7 OS=Caenorhabditis elegans GN=rps-7 PE=3 SV=1	22.0	9.9	1	330.3 (M:330.3)	3	17.0	1.49	26
27		true	BTF3_CAEEL	Transcription factor BTF3 homolog OS=Caenorhabditis elegans GN=icd-1 PE=1 SV=1	17.5	8.7	1	320.5 (M:320.5)	3	23.0	2.74	27
28		true	EF1A_CAEEL	Elongation factor 1-alpha OS=Caenorhabditis elegans GN=eft-3 PE=3 SV=1	50.6	9.1	1	306.3 (M:306.3)	7	13.0	1.29	28
29		true	14331_CAEEL	14-3-3-like protein 1 OS=Caenorhabditis elegans GN=par-5 PE=1 SV=2	28.2	4.7	1	285.5 (M:285.5)	6	23.0	2.84	29
30		true	14332_CAEEL	14-3-3-like protein 2 OS=Caenorhabditis elegans GN=ftt-2 PE=1 SV=1	28.0	4.8	1	275.5 (M:275.5)	4	24.6	4.10	30
31		true	RS3_CAEEL	40S ribosomal protein S3 OS=Caenorhabditis elegans GN=rps-3 PE=3 SV=1	27.3	9.6	1	275.2 (M:275.2)	6	25.1	2.47	31
32		true	RS8_CAEEL	40S ribosomal protein S8 OS=Caenorhabditis elegans GN=rps-8 PE=3 SV=1	23.7	10.6	1	268.6 (M:268.6)	5	21.6	5.70	32
33		true	RLA0_CAEEL	60S acidic ribosomal protein P0 OS=Caenorhabditis elegans GN=rpa-0 PE=1 SV=3	33.8	6.3	1	261.1 (M:261.1)	5	19.2	1.52	33
34		true	G3P2_CAEEL	Glyceraldehyde-3-phosphate dehydrogenase 2 OS=Caenorhabditis elegans GN=gpd-2 PE=3 SV=2	36.4	6.8	4	254.4 (M:254.4)	2	8.5	1.25	34
35		true	RS12_CAEEL	40S ribosomal protein S12 OS=Caenorhabditis elegans GN=rps-12 PE=1 SV=2	15.1	6.2	1	239.0 (M:239.0)	3	20.7	2.91	35
36		true	MYO3_CAEEL	Myosin-3 OS=Caenorhabditis elegans GN=myo-3 PE=2 SV=1	225.4	5.5	1	237.0 (M:237.0)	3	1.9	13.70	36
37		true	RS14_CAEEL	40S ribosomal protein S14 OS=Caenorhabditis elegans GN=rps-14 PE=3 SV=1	16.2	10.4	1	230.5 (M:230.5)	1	7.2	0.40	37
38		true	ODP2_CAEEL	Dihydrolipoyllysine-residue acetyltransferase component of pyruvate dehydrogenase complex, mitochondrial OS=Caenorhabditis elegans GN=F23B12.5 PE=1 SV=1	53.4	8.4	1	225.7 (M:225.7)	2	6.9	6.57	38
39		true	MSP77_CAEEL	Major sperm protein 77/79 OS=Caenorhabditis elegans GN=msp-77 PE=2 SV=3	14.2	7.8	12	211.0 (M:211.0)	2	18.9	1.17	39
40		true	VDAC_CAEEL	Probable voltage-dependent anion-selective channel OS=Caenorhabditis elegans GN=vdac-1 PE=3 SV=2	29.9	9.2	1	208.2 (M:208.2)	3	13.4	2.28	40
41		true	RS4_CAEEL	40S ribosomal protein S4 OS=Caenorhabditis elegans GN=rps-4 PE=1 SV=1	29.0	10.5	1	208.1 (M:208.1)	7	27.4	2.56	41
42		true	RL7_CAEEL	60S ribosomal protein L7 OS=Caenorhabditis elegans GN=rpl-7 PE=3 SV=1	28.1	10.2	1	205.7 (M:205.7)	6	25.4	3.04	42
43		true	HSP7C_CAEEL	Heat shock 70 kDa protein C OS=Caenorhabditis elegans GN=hsp-3 PE=1 SV=2	73.0	5.0	1	196.5 (M:196.5)	4	8.2	6.38	43
44		true	RS2_CAEEL	40S ribosomal protein S2 OS=Caenorhabditis elegans GN=rps-2 PE=3 SV=1	28.9	10.1	1	194.1 (M:194.1)	5	16.9	0.69	44
45		true	VATA_CAEEL	V-type proton ATPase catalytic subunit A OS=Caenorhabditis elegans GN=vha-13 PE=1 SV=3	66.4	5.1	1	191.4 (M:191.4)	3	5.1	11.70	45
46		true	DIM_CAEEL	Disorganized muscle protein 1 OS=Caenorhabditis elegans GN=dim-1 PE=1 SV=3	71.8	8.1	1	184.0 (M:184.0)	5	12.0	6.22	46
47		true	RLA2_CAEEL	60S acidic ribosomal protein P2 OS=Caenorhabditis elegans GN=rpa-2 PE=3 SV=2	10.8	4.5	1	182.9 (M:182.9)	2	30.8	0.57	47
48		true	SYRC_CAEEL	Probable arginine--tRNA ligase, cytoplasmic OS=Caenorhabditis elegans GN=rrt-1 PE=3 SV=2	80.8	7.8	1	182.9 (M:182.9)	2	5.3	11.45	48
49		true	EIF3A_CAEEL	Eukaryotic translation initiation factor 3 subunit A OS=Caenorhabditis elegans GN=egl-45 PE=3 SV=1	124.3	9.1	1	182.9 (M:182.9)	2	2.5	5.40	49
50		true	ATPB_CAEEL	ATP synthase subunit beta, mitochondrial OS=Caenorhabditis elegans GN=atp-2 PE=1 SV=2	57.5	5.5	1	179.3 (M:179.3)	2	4.8	0.72	50
51		true	MDHM_CAEEL	Probable malate dehydrogenase, mitochondrial OS=Caenorhabditis elegans GN=mdh-2 PE=3 SV=1	35.1	9.4	1	179.0 (M:179.0)	5	14.7	2.15	51
52		true	RL44_CAEEL	60S ribosomal protein L44 OS=Caenorhabditis elegans GN=rpl-41 PE=3 SV=2	12.4	10.8	1	176.0 (M:176.0)	2	12.4	0.59	52
53		true	RS17_CAEEL	40S ribosomal protein S17 OS=Caenorhabditis elegans GN=rps-17 PE=3 SV=2	14.9	9.8	1	175.3 (M:175.3)	2	20.8	1.10	53
54		true	IFA2_CAEEL	Intermediate filament protein ifa-2 OS=Caenorhabditis elegans GN=ifa-2 PE=1 SV=1	67.1	5.9	1	173.0 (M:173.0)	2	4.6	0.88	54
55		true	FAR1_CAEEL	Fatty-acid and retinol-binding protein 1 OS=Caenorhabditis elegans GN=far-1 PE=3 SV=1	20.1	7.0	1	168.8 (M:168.8)	1	7.7	1.72	55
56		true	RL11_CAEEL	60S ribosomal protein L11 OS=Caenorhabditis elegans GN=rpl-11.1 PE=3 SV=1	22.7	10.0	1	166.3 (M:166.3)	2	7.1	5.93	56
57		true	EF1B2_CAEEL	Probable elongation factor 1-beta/1-delta 2 OS=Caenorhabditis elegans GN=eef-1B.2 PE=1 SV=4	28.2	4.9	1	165.8 (M:165.8)	1	6.8	0.93	57
58		true	PPN1_CAEEL	Papilin OS=Caenorhabditis elegans GN=mig-6 PE=1 SV=1	237.4	4.9	1	164.8 (M:164.8)	4	2.7	4.29	58
59		true	FABP6_CAEEL	Fatty acid-binding protein homolog 6 OS=Caenorhabditis elegans GN=lbp-6 PE=1 SV=1	15.6	6.8	1	162.2 (M:162.2)	2	16.3	2.59	59
60		true	KARG1_CAEEL	Probable arginine kinase F46H5.3 OS=Caenorhabditis elegans GN=F46H5.3 PE=1 SV=2	44.1	6.8	1	160.5 (M:160.5)	3	7.6	3.29	60
61		true	R23A2_CAEEL	60S ribosomal protein L23a 2 OS=Caenorhabditis elegans GN=rpl-25.2 PE=3 SV=1	16.3	10.6	2	160.0 (M:160.0)	2	16.4	0.59	61
62		true	TNNI2_CAEEL	Troponin I 2 OS=Caenorhabditis elegans GN=unc-27 PE=2 SV=2	27.5	5.5	1	152.8 (M:152.8)	1	15.3	0.83	62
63		true	LIN10_CAEEL	Protein lin-10 OS=Caenorhabditis elegans GN=lin-10 PE=1 SV=1	105.2	5.1	1	151.1 (M:151.1)	1	1.6	7.08	63
64		true	UCR1_CAEEL	Cytochrome b-c1 complex subunit 1, mitochondrial OS=Caenorhabditis elegans GN=ucr-1 PE=3 SV=2	51.7	6.1	1	148.5 (M:148.5)	3	8.1	4.81	64
65		true	CCHL_CAEEL	Probable cytochrome c-type heme lyase OS=Caenorhabditis elegans GN=cchl-1 PE=3 SV=1	31.2	6.2	1	142.6 (M:142.6)	1	7.1	2.88	65
66		true	VATL2_CAEEL	V-type proton ATPase 16 kDa proteolipid subunit 2/3 OS=Caenorhabditis elegans GN=vha-2 PE=2 SV=2	16.4	8.5	1	138.4 (M:138.4)	1	7.5	1.33	66
67		true	RL18_CAEEL	60S ribosomal protein L18 OS=Caenorhabditis elegans GN=rpl-18 PE=3 SV=1	21.0	11.4	1	137.0 (M:137.0)	1	6.9	0.36	67
68		true	EIF3C_CAEEL	Eukaryotic translation initiation factor 3 subunit C OS=Caenorhabditis elegans GN=eif-3.C PE=3 SV=2	103.8	5.8	1	136.8 (M:136.8)	2	3.1	5.78	68
69		true	IF4E3_CAEEL	Eukaryotic translation initiation factor 4E-3 OS=Caenorhabditis elegans GN=ife-3 PE=1 SV=2	28.2	5.9	1	136.4 (M:136.4)	1	8.8	0.12	69
70		true	UNC22_CAEEL	Twitchin OS=Caenorhabditis elegans GN=unc-22 PE=1 SV=3	788.5	5.8	1	136.4 (M:136.4)	3	0.4	4.81	70
71		true	RL40_CAEEL	Ubiquitin-60S ribosomal protein L40 OS=Caenorhabditis elegans GN=ubq-2 PE=3 SV=2	14.6	10.6	2	133.8 (M:133.8)	2	19.5	1.49	71
72		true	RL18A_CAEEL	60S ribosomal protein L18a OS=Caenorhabditis elegans GN=rpl-20 PE=3 SV=2	20.9	10.4	1	133.0 (M:133.0)	2	15.0	1.65	72
73		true	UNC87_CAEEL	Protein unc-87 OS=Caenorhabditis elegans GN=unc-87 PE=1 SV=3	62.7	9.1	1	123.8 (M:123.8)	3	6.5	1.89	73
74		true	TBA2_CAEEL	Tubulin alpha-2 chain OS=Caenorhabditis elegans GN=tba-2 PE=2 SV=1	49.9	4.9	3	123.5 (M:123.5)	4	10.0	1.70	74
75		true	MPCP_CAEEL	Phosphate carrier protein, mitochondrial OS=Caenorhabditis elegans GN=F01G4.6 PE=2 SV=1	36.6	9.1	1	121.3 (M:121.3)	5	14.4	7.65	75
76		true	PSA5_CAEEL	Proteasome subunit alpha type-5 OS=Caenorhabditis elegans GN=pas-5 PE=1 SV=1	27.2	5.3	1	120.6 (M:120.6)	2	9.7	3.82	76
77		true	RS28_CAEEL	40S ribosomal protein S28 OS=Caenorhabditis elegans GN=rps-28 PE=3 SV=1	7.4	10.7	1	119.9 (M:119.9)	2	32.3	6.63	77
78		true	MMSA_CAEEL	Probable methylmalonate-semialdehyde dehydrogenase [acylating], mitochondrial OS=Caenorhabditis elegans GN=alh-8 PE=1 SV=1	56.4	7.6	1	116.3 (M:116.3)	3	6.5	2.21	78
79		true	RL19_CAEEL	60S ribosomal protein L19 OS=Caenorhabditis elegans GN=rpl-19 PE=3 SV=1	23.6	11.4	1	115.5 (M:115.5)	3	13.1	2.04	79
80		true	U2AF2_CAEEL	Splicing factor U2AF 65 kDa subunit OS=Caenorhabditis elegans GN=uaf-1 PE=2 SV=2	55.4	6.1	1	115.2 (M:115.2)	1	2.8	5.61	80
81		true	RL4_CAEEL	60S ribosomal protein L4 OS=Caenorhabditis elegans GN=rpl-4 PE=1 SV=3	38.6	11.2	1	114.6 (M:114.6)	5	13.0	4.00	81

82		true	RL5_CAEEL	60S ribosomal protein L5 OS=Caenorhabditis elegans GN=rpl-5 PE=3 SV=1	33.4	9.8	1	114.3 (M:114.3)	4	18.4	6.18	82
83		true	PGL1_CAEEL	P granule abnormality protein 1 OS=Caenorhabditis elegans GN=pgl-1 PE=1 SV=1	78.4	4.9	1	111.4 (M:111.4)	2	5.5	6.17	83
84		true	PDI2_CAEEL	Protein disulfide-isomerase 2 OS=Caenorhabditis elegans GN=pdi-2 PE=1 SV=1	55.1	4.7	1	108.8 (M:108.8)	4	8.7	2.24	84
85		true	NACA_CAEEL	Nascent polypeptide-associated complex subunit alpha OS=Caenorhabditis elegans GN=Y65B4BR.5 PE=1 SV=1	22.1	5.0	1	107.5 (M:107.5)	2	11.2	5.40	85
86		true	ODPA_CAEEL	Probable pyruvate dehydrogenase E1 component subunit alpha, mitochondrial OS=Caenorhabditis elegans GN=T05H10.6 PE=3 SV=1	43.8	7.1	1	105.3 (M:105.3)	2	5.8	2.36	86
87		true	IFB1_CAEEL	Intermediate filament protein ifb-1 OS=Caenorhabditis elegans GN=ifb-1 PE=1 SV=1	67.1	5.9	1	100.7 (M:100.7)	2	2.9	2.36	87
88		true	LARP1_CAEEL	La-related protein 1 OS=Caenorhabditis elegans GN=larp-1 PE=3 SV=2	128.2	9.0	1	99.0 (M:99.0)	1	1.0	0.00	88
89		true	PDIA6_CAEEL	Probable protein disulfide-isomerase A6 OS=Caenorhabditis elegans GN=tag-320 PE=3 SV=1	47.7	5.8	1	98.3 (M:98.3)	2	7.3	4.32	89
90		true	YM67_CAEEL	Putative serine protease K12H4.7 OS=Caenorhabditis elegans GN=K12H4.7 PE=3 SV=2	56.5	5.8	1	98.2 (M:98.2)	3	5.5	3.40	90
91		true	HSP7D_CAEEL	Heat shock 70 kDa protein D OS=Caenorhabditis elegans GN=hsp-4 PE=1 SV=2	72.2	5.0	1	97.6 (M:97.6)	1	1.7	2.81	91
92		true	GBLP_CAEEL	Guanine nucleotide-binding protein subunit beta-2-like 1 OS=Caenorhabditis elegans GN=rack-1 PE=1 SV=3	35.8	6.4	1	93.8 (M:93.8)	2	5.8	2.62	92
93		true	LMN1_CAEEL	Lamin-1 OS=Caenorhabditis elegans GN=lmn-1 PE=1 SV=2	64.0	5.4	1	93.6 (M:93.6)	3	6.5	4.26	93
94		true	DENR_CAEEL	Density-regulated protein homolog OS=Caenorhabditis elegans GN=Y47D3A.21 PE=3 SV=1	20.3	5.4	1	93.3 (M:93.3)	1	7.3	2.39	94
95		true	RSP3_CAEEL	Probable splicing factor, arginine/serine-rich 3 OS=Caenorhabditis elegans GN=rsp-3 PE=1 SV=2	28.7	10.3	1	91.2 (M:91.2)	2	10.9	2.40	95
96		true	FBRL_CAEEL	rRNA 2'-O-methyltransferase fibrillarin OS=Caenorhabditis elegans GN=fib-1 PE=3 SV=1	36.4	10.3	1	90.9 (M:90.9)	1	3.7	1.00	96
97		true	AL7A1_CAEEL	Putative aldehyde dehydrogenase family 7 member A1 homolog OS=Caenorhabditis elegans GN=alh-9 PE=3 SV=2	57.0	6.3	1	90.7 (M:90.7)	3	5.5	5.31	97
98		true	ACOC_CAEEL	Probable cytoplasmic aconitate hydratase OS=Caenorhabditis elegans GN=aco-1 PE=1 SV=1	96.6	5.5	1	89.6 (M:89.6)	1	1.8	6.22	98
99		true	CALR_CAEEL	Calreticulin OS=Caenorhabditis elegans GN=crt-1 PE=1 SV=1	45.6	4.6	1	87.7 (M:87.7)	2	5.3	0.86	99
100		true	ASP6_CAEEL	Aspartic protease 6 OS=Caenorhabditis elegans GN=asp-6 PE=3 SV=1	41.5	5.6	1	85.2 (M:85.2)	3	9.3	2.09	100
101		true	ETFA_CAEEL	Probable electron transfer flavoprotein subunit alpha, mitochondrial OS=Caenorhabditis elegans GN=F27D4.1 PE=3 SV=2	34.4	9.2	1	84.9 (M:84.9)	3	12.0	1.53	101
102		true	ENO_CAEEL	Enolase OS=Caenorhabditis elegans GN=enol-1 PE=1 SV=3	46.6	5.5	1	84.9 (M:84.9)	2	7.4	7.29	102
103		true	ATPD_CAEEL	ATP synthase subunit delta, mitochondrial OS=Caenorhabditis elegans GN=F58F12.1 PE=1 SV=1	16.9	4.8	1	83.9 (M:83.9)	4	25.8	1.20	103
104		true	TBB2_CAEEL	Tubulin beta-2 chain OS=Caenorhabditis elegans GN=tbb-2 PE=3 SV=1	50.3	4.8	1	83.1 (M:83.1)	3	5.3	1.35	104
105		true	RL10_CAEEL	60S ribosomal protein L10 OS=Caenorhabditis elegans GN=rpl-10 PE=3 SV=1	24.7	10.4	1	82.1 (M:82.1)	1	4.7	0.80	105
106		true	FAR2_CAEEL	Fatty-acid and retinol-binding protein 2 OS=Caenorhabditis elegans GN=far-2 PE=1 SV=1	20.0	5.7	1	80.4 (M:80.4)	2	11.5	10.42	106
107		true	YP69_CAEEL	Uncharacterized protein B0495.9 OS=Caenorhabditis elegans GN=B0495.9 PE=4 SV=1	30.0	9.7	1	79.5 (M:79.5)	1	5.2	13.03	107
108		true	RS3A_CAEEL	40S ribosomal protein S3a OS=Caenorhabditis elegans GN=rps-1 PE=3 SV=2	28.9	9.6	1	78.9 (M:78.9)	3	12.8	3.76	108
109		true	MT2_CAEEL	Metallothionein-2 OS=Caenorhabditis elegans GN=mtl-2 PE=1 SV=2	6.6	7.3	1	77.3 (M:77.3)	3	34.9	2.31	109
110		true	RL3_CAEEL	60S ribosomal protein L3 OS=Caenorhabditis elegans GN=rpl-3 PE=2 SV=1	45.6	10.4	1	75.5 (M:75.5)	3	5.5	0.62	110
111		true	YUA6_CAEEL	Uncharacterized serine carboxypeptidase F13S12.6 OS=Caenorhabditis elegans GN=F13D12.6 PE=3 SV=1	50.1	6.3	1	72.4 (M:72.4)	1	2.4	2.64	111
112		true	MYO2_CAEEL	Myosin-2 OS=Caenorhabditis elegans GN=myo-2 PE=1 SV=2	222.9	6.0	1	71.3 (M:71.3)	2	1.4	2.20	112
113		true	RS5_CAEEL	40S ribosomal protein S5 OS=Caenorhabditis elegans GN=rps-5 PE=3 SV=1	23.1	9.8	1	70.6 (M:70.6)	3	12.4	2.69	113
114		true	RHO1_CAEEL	Ras-like GTP-binding protein rhoA OS=Caenorhabditis elegans GN=rho-1 PE=1 SV=1	21.6	6.0	1	69.7 (M:69.7)	1	8.9	5.38	114
115		true	PDI1_CAEEL	Protein disulfide-isomerase 1 OS=Caenorhabditis elegans GN=pdi-1 PE=3 SV=1	53.4	4.6	1	69.5 (M:69.5)	4	11.8	7.02	115
116		true	RL27_CAEEL	60S ribosomal protein L27 OS=Caenorhabditis elegans GN=rpl-27 PE=2 SV=1	15.7	10.8	1	69.3 (M:69.3)	3	13.2	3.20	116
117		true	OLA1_CAEEL	Obg-like ATPase 1 OS=Caenorhabditis elegans GN=ola-1 PE=3 SV=1	44.3	6.4	1	69.0 (M:69.0)	2	5.6	3.84	117
118		true	RL36_CAEEL	60S ribosomal protein L36 OS=Caenorhabditis elegans GN=rpl-36 PE=1 SV=3	11.9	11.7	1	67.6 (M:67.6)	2	20.2	3.17	118
119		true	RL6_CAEEL	60S ribosomal protein L6 OS=Caenorhabditis elegans GN=rpl-6 PE=1 SV=1	24.3	11.0	1	67.0 (M:67.0)	3	13.8	3.17	119
120		true	IPYR_CAEEL	Probable inorganic pyrophosphatase 1 OS=Caenorhabditis elegans GN=pyp-1 PE=3 SV=4	46.4	5.8	1	66.0 (M:66.0)	2	4.7	2.60	120
121		true	CSK2A_CAEEL	Casein kinase II subunit alpha OS=Caenorhabditis elegans GN=kin-3 PE=1 SV=1	42.2	6.4	1	65.4 (M:65.4)	2	6.7	10.06	121
122		true	CGH1_CAEEL	ATP-dependent RNA helicase cgh-1 OS=Caenorhabditis elegans GN=cgh-1 PE=1 SV=1	48.7	8.3	1	63.1 (M:63.1)	2	4.0	4.03	122
123		true	ACBP1_CAEEL	Acyl-CoA-binding protein homolog 1 OS=Caenorhabditis elegans GN=acbp-1 PE=3 SV=1	9.4	6.2	1	60.2 (M:60.2)	2	25.6	0.68	123
124		true	RL22_CAEEL	60S ribosomal protein L22 OS=Caenorhabditis elegans GN=rpl-22 PE=1 SV=3	14.9	9.8	1	60.2 (M:60.2)	2	15.4	0.85	124
125		true	CRI3_CAEEL	Conserved regulator of innate immunity protein 3 OS=Caenorhabditis elegans GN=cri-3 PE=1 SV=2	26.4	4.9	1	60.2 (M:60.2)	2	11.4	1.76	125
126		true	TNNT_CAEEL	Troponin T OS=Caenorhabditis elegans GN=mup-2 PE=2 SV=1	47.0	4.8	1	59.7 (M:59.7)	1	8.1	3.43	126
127		true	DLDH_CAEEL	Dihydrolipoyl dehydrogenase, mitochondrial OS=Caenorhabditis elegans GN=dld-1 PE=3 SV=2	52.6	7.6	1	59.1 (M:59.1)	2	4.0	3.50	127
128		true	U375A_CAEEL	UPF0375 protein C08F11.11 OS=Caenorhabditis elegans GN=C08F11.11 PE=1 SV=1	11.8	5.7	1	57.5 (M:57.5)	3	19.8	2.76	128
129		true	LE767_CAEEL	Very-long-chain 3-oxooacyl-coA reductase let-767 OS=Caenorhabditis elegans GN=let-767 PE=1 SV=2	34.3	9.5	3	56.3 (M:56.3)	2	10.1	3.98	129
130		true	YSI1_CAEEL	Uncharacterized protein F15G9.1 OS=Caenorhabditis elegans GN=F15G9.1 PE=4 SV=1	30.7	4.5	1	55.2 (M:55.2)	2	13.3	10.88	130
131		true	CLH_CAEEL	Probable clathrin heavy chain 1 OS=Caenorhabditis elegans GN=chc-1 PE=3 SV=1	191.4	5.7	1	54.4 (M:54.4)	2	1.4	3.74	131
132		true	VATG_CAEEL	Probable V-type proton ATPase subunit G OS=Caenorhabditis elegans GN=vha-10 PE=3 SV=1	14.5	9.6	1	54.3 (M:54.3)	2	15.1	1.34	132
133		true	PGK_CAEEL	Probable phosphoglycerate kinase OS=Caenorhabditis elegans GN=pgk-1 PE=1 SV=1	44.1	6.5	1	53.8 (M:53.8)	1	4.8	5.21	133
134		true	ALF2_CAEEL	Fructose-bisphosphate aldolase 2 OS=Caenorhabditis elegans GN=aldo-2 PE=2 SV=1	38.8	7.7	1	50.5 (M:50.5)	2	6.3	1.31	134
135		true	RL37A_CAEEL	60S ribosomal protein L37a OS=Caenorhabditis elegans GN=rpl-43 PE=3 SV=3	10.1	10.4	1	50.4 (M:50.4)	2	18.7	1.89	135
136		true	GCC7_CAEEL	Germ cell-expressed protein R06C7.1 OS=Caenorhabditis elegans GN=R06C7.1 PE=2 SV=1	105.4	8.7	1	50.3 (M:50.3)	1	1.2	1.97	136
137		true	SIP1_CAEEL	Stress-induced protein 1 OS=Caenorhabditis elegans GN=sip-1 PE=1 SV=1	17.8	7.9	1	49.9 (M:49.9)	2	13.8	2.37	137
138		true	RS6_CAEEL	40S ribosomal protein S6 OS=Caenorhabditis elegans GN=rps-6 PE=1 SV=1	28.1	10.8	1	49.7 (M:49.7)	2	8.1	0.69	138
139		true	RL9_CAEEL	60S ribosomal protein L9 OS=Caenorhabditis elegans GN=rpl-9 PE=3 SV=1	21.5	10.1	1	49.4 (M:49.4)	1	10.1	0.42	139
140		true	RS25_CAEEL	40S ribosomal protein S25 OS=Caenorhabditis elegans GN=rps-25 PE=3 SV=1	12.9	10.1	1	49.1 (M:49.1)	1	8.5	0.85	140
141		true	CYC21_CAEEL	Cytochrome c 2.1 OS=Caenorhabditis elegans GN=cyc-2.1 PE=1 SV=2	12.2	9.9	1	48.8 (M:48.8)	2	20.7	2.21	141
142		true	RL24_CAEEL	60S ribosomal protein L24 OS=Caenorhabditis elegans GN=rpl-24.1 PE=3 SV=1	17.8	11.3	1	48.6 (M:48.6)	2	12.6	4.19	142
143		true	VATH2_CAEEL	Probable V-type proton ATPase subunit H 2 OS=Caenorhabditis elegans GN=vha-15 PE=3 SV=1	54.2	5.9	1	48.4 (M:48.4)	1	3.0	4.03	143
144		true	H2B1_CAEEL	Histone H2B 1 OS=Caenorhabditis elegans GN=his-11 PE=1 SV=4	13.5	10.4	4	48.1 (M:48.1)	2	15.6	0.68	144
145		true	OAT_CAEEL	Probable ornithine aminotransferase, mitochondrial OS=Caenorhabditis elegans GN=C16A3.10 PE=3 SV=3	46.4	8.7	1	45.4 (M:45.4)	2	3.3	7.58	145
146		true	UNC89_CAEEL	Muscle M-line assembly protein unc-89 OS=Caenorhabditis elegans GN=unc-89 PE=1 SV=3	893.7	5.4	1	44.1 (M:44.1)	2	0.3	2.18	146
147		true	ATIF2_CAEEL	ATPase inhibitor mai-2, mitochondrial OS=Caenorhabditis elegans GN=mai-2 PE=3 SV=1	12.0	8.1	1	43.5 (M:43.5)	2	14.7	1.02	147
148		true	SYV_CAEEL	Valine--tRNA ligase OS=Caenorhabditis elegans GN=vrs-2 PE=1 SV=1	118.8	6.3	1	43.4 (M:43.4)	2	2.6	1.63	148
149		true	KC1A_CAEEL	Casein kinase I isoform alpha OS=Caenorhabditis elegans GN=kin-19 PE=3 SV=1	39.0	9.6	1	43.0 (M:43.0)	1	11.1	7.12	149
150		true	IFA1_CAEEL	Intermediate filament protein ifa-1 OS=Caenorhabditis elegans GN=ifa-1 PE=1 SV=2	66.5	6.1	1	42.6 (M:42.6)	1	1.7	2.19	150
151		true	ECHM_CAEEL	Probable enoyl-CoA hydratase, mitochondrial OS=Caenorhabditis elegans GN=ech-6 PE=1 SV=1	31.2	8.6	1	42.5 (M:42.5)	1	4.5	2.63	151
152		true	RL23_CAEEL	60S ribosomal protein L23 OS=Caenorhabditis elegans GN=rpl-23 PE=3 SV=1	14.9	10.5	1	42.4 (M:42.4)	1	7.1	0.23	152
153		true	DIG1_CAEEL	Mesocentin OS=Caenorhabditis elegans GN=dig-1 PE=1 SV=2	1372.5	4.1	1	42.2 (M:42.2)	1	0.1	5.39	153
154		true	RL31_CAEEL	60S ribosomal protein L31 OS=Caenorhabditis elegans GN=rpl-31 PE=3 SV=1	14.3	10.1	1	41.4 (M:41.4)	1	7.4	0.65	154
155		true	SYDC_CAEEL	Aspartate--tRNA ligase, cytoplasmic OS=Caenorhabditis elegans GN=drs-1 PE=3 SV=1	59.9	6.0	1	40.8 (M:40.8)	1	3.4	2.23	155
156		true	PSA7_CAEEL	Proteasome subunit alpha type-7 OS=Caenorhabditis elegans GN=pas-4 PE=1 SV=1	28.2	5.9	1	40.7 (M:40.7)	1	7.9	8.16	156
157		true	DHSD_CAEEL	Putative succinate dehydrogenase [ubiquinone] cytochrome b small subunit, mitochondrial OS=Caenorhabditis elegans GN=sdhd-1 PE=3 SV=1	15.8	9.4	1	40.5 (M:40.5)	1	6.9	2.51	157
158		true	F37C4_CAEEL	Protein F37C4.5 OS=Caenorhabditis elegans GN=F37C4.5 PE=1 SV=3	61.4	5.5	1	40.1 (M:40.1)	1	2.0	1.18	158
159		true	TDX1_CAEEL	Probable peroxiredoxin prdx-3 OS=Caenorhabditis elegans GN=prdx-3 PE=1 SV=1	24.9	6.9	1	39.4 (M:39.4)	2	7.5	1.83	159
160		true	SODC_CAEEL	Superoxide dismutase [Cu-Zn] OS=Caenorhabditis elegans GN=sod-1 PE=1 SV=2	18.7	6.1	1	37.8 (M:37.8)	1	7.2	1.44	160
161		true	RL35_CAEEL	60S ribosomal protein L35 OS=Caenorhabditis elegans GN=rpl-35 PE=3 SV=1	14.2	11.3	1	37.6 (M:37.6)	1	8.1	1.14	161
162		true	CWC22_CAEEL	Pre-mRNA-splicing factor CWC22 homolog OS=Caenorhabditis elegans GN=let-858 PE=2 SV=1	104.2	6.4	1	36.7 (M:36.7)	1	1.6	4.38	162
163		true	GLH1_CAEEL	ATP-dependent RNA helicase glh-1 OS=Caenorhabditis elegans GN=glh-1 PE=1 SV=3	79.7	5.6	1	34.0 (M:34.0)	1	6.2	12.61	163
164		true	PSA1_CAEEL	Proteasome subunit alpha type-1 OS=Caenorhabditis elegans GN=pas-6 PE=1 SV=1	28.3	6.5	1	33.1 (M:33.1)	1	5.8	9.51	164
165		true	YLA4_CAEEL	Uncharacterized protein C30C11.4 OS=Caenorhabditis elegans GN=C30C11.4 PE=1 SV=1	86.8	5.3	1	32.8 (M:32.8)	1	2.4	3.04	165
166		true	PYC1_CAEEL	Pyruvate carboxylase 1 OS=Caenorhabditis elegans GN=pyc-1 PE=1 SV=1	129.2	6.5	1	32.7 (M:32.7)	2	2.5	6.95	166
167		true	YRU4_CAEEL	Uncharacterized protein T09B9.4 OS=Caenorhabditis elegans GN=T09B9.4 PE=4 SV=1	59.6	6.5	1	32.7 (M:32.7)	1	2.1	17.34	167

168		true	RL8_CAEEL	60S ribosomal protein L8 OS=Caenorhabditis elegans GN=rpl-8 PE=3 SV=1	28.2	11.1	1	31.9 (M:31.9)	1	10.0	7.26	168
169		true	EF1B1_CAEEL	Probable elongation factor 1-beta/1-delta 1 OS=Caenorhabditis elegans GN=eef-1B.1 PE=1 SV=1	22.7	4.4	1	31.8 (M:31.8)	1	4.2	0.59	169
170		true	RS9_CAEEL	40S ribosomal protein S9 OS=Caenorhabditis elegans GN=rps-9 PE=3 SV=1	21.9	10.5	1	30.3 (M:30.3)	1	4.2	0.04	170
171		true	RL35A_CAEEL	60S ribosomal protein L35a OS=Caenorhabditis elegans GN=rpl-33 PE=1 SV=3	13.8	11.3	1	30.1 (M:30.1)	1	7.3	0.78	171
172		true	TTR16_CAEEL	Transthyretin-like protein 16 OS=Caenorhabditis elegans GN=ttr-16 PE=1 SV=2	14.7	5.2	1	30.1 (M:30.1)	1	8.3	4.75	172
173		true	TTR2_CAEEL	Transthyretin-like protein 2 OS=Caenorhabditis elegans GN=ttr-2 PE=1 SV=1	15.7	8.3	1	30.1 (M:30.1)	1	6.8	2.55	173
174		true	FABP2_CAEEL	Fatty acid-binding protein homolog 2 OS=Caenorhabditis elegans GN=lbp-2 PE=1 SV=1	18.8	6.2	1	30.1 (M:30.1)	1	6.2	2.14	174
175		true	RT25_CAEEL	Probable 28S ribosomal protein S25, mitochondrial OS=Caenorhabditis elegans GN=mrps-25 PE=3 SV=1	19.8	9.3	1	30.1 (M:30.1)	1	5.9	0.32	175
176		true	RAN_CAEEL	GTP-binding nuclear protein ran-1 OS=Caenorhabditis elegans GN=ran-1 PE=1 SV=1	24.2	7.0	1	30.1 (M:30.1)	1	5.1	3.09	176
177		true	LMP1_CAEEL	LAMP family protein lmp-1 OS=Caenorhabditis elegans GN=lmp-1 PE=2 SV=1	25.8	5.2	1	30.1 (M:30.1)	1	7.2	13.60	177
178		true	LEC1_CAEEL	32 kDa beta-galactoside-binding lectin OS=Caenorhabditis elegans GN=lec-1 PE=1 SV=1	31.8	6.1	1	30.1 (M:30.1)	1	4.7	4.34	178
179		true	AT1B1_CAEEL	Sodium/potassium-transporting ATPase subunit beta-1 OS=Caenorhabditis elegans GN=nkb-1 PE=1 SV=1	36.6	7.5	1	30.1 (M:30.1)	1	4.1	1.65	179
180		true	STIP1_CAEEL	Stress-induced-phosphoprotein 1 OS=Caenorhabditis elegans GN=sti-1 PE=1 SV=1	36.9	6.6	1	30.1 (M:30.1)	1	4.1	3.90	180
181		true	SYSC_CAEEL	Probable serine--tRNA ligase, cytoplasmic OS=Caenorhabditis elegans GN=srs-2 PE=3 SV=1	55.2	5.9	1	30.1 (M:30.1)	1	2.7	5.19	181
182		true	SYNC_CAEEL	Asparagine--tRNA ligase, cytoplasmic OS=Caenorhabditis elegans GN=nrs-1 PE=3 SV=1	61.1	5.9	1	30.1 (M:30.1)	1	2.2	10.18	182
183		true	KCC2D_CAEEL	Calcium/calmodulin-dependent protein kinase type II OS=Caenorhabditis elegans GN=unc-43 PE=1 SV=2	79.9	8.7	1	30.1 (M:30.1)	1	2.5	3.79	183
184		true	YQ83_CAEEL	GYF domain-containing protein C18H9.3 OS=Caenorhabditis elegans GN=C18H9.3/C18H9.2 PE=3 SV=3	113.4	9.1	1	30.1 (M:30.1)	1	1.2	2.30	184
185		true	ODO1_CAEEL	2-oxoglutarate dehydrogenase, mitochondrial OS=Caenorhabditis elegans GN=ogdh-1 PE=1 SV=2	115.6	6.3	1	30.1 (M:30.1)	1	4.1	9.88	185
186		true	COX6A_CAEEL	Probable cytochrome c oxidase subunit 6A, mitochondrial OS=Caenorhabditis elegans GN=tag-174 PE=3 SV=1	14.7	9.6	1	30.0 (M:30.0)	1	7.8	5.10	186
187		true	RL26_CAEEL	60S ribosomal protein L26 OS=Caenorhabditis elegans GN=rpl-26 PE=3 SV=1	16.1	11.3	1	29.7 (M:29.7)	1	7.7	1.82	187
188		true	RL10A_CAEEL	60S ribosomal protein L10a OS=Caenorhabditis elegans GN=rpl-10a PE=3 SV=1	24.1	9.9	1	29.6 (M:29.6)	1	6.0	1.28	188
189		true	SSP9_CAEEL	Sperm-specific class P protein 9/11 OS=Caenorhabditis elegans GN=ssp-9 PE=2 SV=1	11.0	6.7	4	29.5 (M:29.5)	1	7.3	1.94	189
190		true	H331_CAEEL	Histone H3.3 type 1 OS=Caenorhabditis elegans GN=his-71 PE=2 SV=2	15.3	11.3	3	29.3 (M:29.3)	1	5.1	0.79	190
191		true	TCPQ_CAEEL	T-complex protein 1 subunit theta OS=Caenorhabditis elegans GN=cct-8 PE=1 SV=3	59.7	5.8	1	28.8 (M:28.8)	1	2.2	5.42	191
192		true	YZVL_CAEEL	Uncharacterized NOP5 family protein K07C5.4 OS=Caenorhabditis elegans GN=K07C5.4 PE=3 SV=1	54.5	8.7	1	28.4 (M:28.4)	1	2.7	8.22	192
193		true	RL12_CAEEL	60S ribosomal protein L12 OS=Caenorhabditis elegans GN=rpl-12 PE=3 SV=1	17.8	9.5	1	26.7 (M:26.7)	1	5.5	0.52	193
194		true	CALM_CAEEL	Calmodulin OS=Caenorhabditis elegans GN=cmd-1 PE=1 SV=3	16.8	4.1	1	25.0 (M:25.0)	1	8.1	8.83	194
195		true	PRS10_CAEEL	Probable 26S protease regulatory subunit 10B OS=Caenorhabditis elegans GN=rpt-4 PE=1 SV=2	45.8	7.6	1	23.2 (M:23.2)	1	2.2	2.96	195
196		true	TIM9_CAEEL	Mitochondrial import inner membrane translocase subunit Tim9 OS=Caenorhabditis elegans GN=tin-9.1 PE=3 SV=1	10.2	7.7	1	21.1 (M:21.1)	1	12.2	7.10	196
197		true	PAT2_CAEEL	Integrin alpha pat-2 OS=Caenorhabditis elegans GN=pat-2 PE=1 SV=1	135.9	5.8	1	20.8 (M:20.8)	1	0.9	7.67	197
198		true	H15_CAEEL	Histone H1.5 OS=Caenorhabditis elegans GN=hil-5 PE=2 SV=3	23.4	10.8	1	20.3 (M:20.3)	1	4.0	3.61	198
199		true	ALF1_CAEEL	Fructose-bisphosphate aldolase 1 OS=Caenorhabditis elegans GN=aldo-1 PE=1 SV=1	39.2	6.3	1	20.3 (M:20.3)	1	6.0	11.06	199
200		true	SYTC_CAEEL	Threonine--tRNA ligase, cytoplasmic OS=Caenorhabditis elegans GN=trs-1 PE=3 SV=1	84.4	7.1	1	20.1 (M:20.1)	1	1.2	7.45	200
201		true	FABP9_CAEEL	Fatty acid-binding protein homolog 9 OS=Caenorhabditis elegans GN=lbp-9 PE=3 SV=1	16.9	7.6	1	20.0 (M:20.0)	1	5.3	0.87	201
202		true	METK1_CAEEL	Probable S-adenosylmethionine synthase 1 OS=Caenorhabditis elegans GN=sams-1 PE=1 SV=1	43.6	6.0	1	19.9 (M:19.9)	1	2.5	0.80	202
203		true	DEOC_CAEEL	Putative deoxyribose-phosphate aldolase OS=Caenorhabditis elegans GN=F09E5.3 PE=3 SV=1	33.1	7.0	1	19.9 (M:19.9)	1	4.3	4.31	203
204		true	STT3_CAEEL	Dolichyl-diphosphooligosaccharide--protein glycosyltransferase subunit STT3 OS=Caenorhabditis elegans GN=T12A2.2 PE=1 SV=1	85.1	8.9	1	19.9 (M:19.9)	1	2.0	19.79	204
205		true	ADF1_CAEEL	Actin-depolymerizing factor 1, isoforms a/b OS=Caenorhabditis elegans GN=unc-60 PE=1 SV=2	23.6	5.6	1	19.9 (M:19.9)	1	3.8	1.25	205
206		true	YOT9_CAEEL	Uncharacterized protein ZK632.9 OS=Caenorhabditis elegans GN=ZK632.9 PE=4 SV=3	6.6	9.2	1	19.9 (M:19.9)	1	17.7	0.92	206
207		true	IFB2_CAEEL	Intermediate filament protein ifb-2 OS=Caenorhabditis elegans GN=ifb-2 PE=1 SV=1	61.6	5.4	1	19.8 (M:19.8)	1	1.7	3.42	207
208		true	PRP19_CAEEL	Pre-mRNA-processing factor 19 OS=Caenorhabditis elegans GN=prp-19 PE=3 SV=2	53.2	5.7	1	19.8 (M:19.8)	1	2.0	0.28	208
209		true	TPIS_CAEEL	Triosephosphate isomerase OS=Caenorhabditis elegans GN=tpi-1 PE=1 SV=2	26.6	6.2	1	19.8 (M:19.8)	1	4.0	4.39	209
210		true	ODB2_CAEEL	Lipoamide acyltransferase component of branched-chain alpha-keto acid dehydrogenase complex, mitochondrial OS=Caenorhabditis elegans GN=ZK669.4 PE=3 SV=1	49.7	8.7	1	19.8 (M:19.8)	1	2.2	2.36	210
211		true	SYFB_CAEEL	Phenylalanine--tRNA ligase beta subunit OS=Caenorhabditis elegans GN=frs-2 PE=1 SV=2	66.0	5.7	1	19.8 (M:19.8)	1	1.5	4.85	211
212		true	RL28_CAEEL	60S ribosomal protein L28 OS=Caenorhabditis elegans GN=rpl-28 PE=1 SV=3	13.7	11.7	1	19.7 (M:19.7)	1	7.1	2.36	212
213		true	SYAC_CAEEL	Alanine--tRNA ligase, cytoplasmic OS=Caenorhabditis elegans GN=aars-2 PE=2 SV=1	106.7	5.5	1	19.4 (M:19.4)	1	1.0	2.11	213
214		true	YBYK_CAEEL	TPPP family protein C32E8.3 OS=Caenorhabditis elegans GN=C32E8.3 PE=1 SV=1	19.4	9.4	1	18.7 (M:18.7)	1	5.0	0.34	214
215		true	VMS1_CAEEL	Protein vms-1 OS=Caenorhabditis elegans GN=vms-1 PE=2 SV=2	71.3	6.3	1	18.5 (M:18.5)	1	2.1	0.74	215
216		true	CISY_CAEEL	Probable citrate synthase, mitochondrial OS=Caenorhabditis elegans GN=cts-1 PE=1 SV=1	51.5	7.7	1	16.9 (M:16.9)	1	2.4	0.02	216
217		true	STX5_CAEEL	Putative syntaxin-5 OS=Caenorhabditis elegans GN=syx-5 PE=3 SV=1	45.8	6.1	1	15.7 (M:15.7)	1	5.6	4.86	217
218		true	YZ10_CAEEL	Uncharacterized protein F08B12.4 OS=Caenorhabditis elegans GN=F08B12.4 PE=4 SV=2	10.3	6.4	1	15.5 (M:15.5)	1	9.8	1.12	218
219		true	U562_CAEEL	UPF0562 protein C29E4.12 OS=Caenorhabditis elegans GN=C29E4.12 PE=3 SV=1	12.1	9.5	1	14.4 (M:14.4)	1	9.3	6.97	219
220		true	NH2L1_CAEEL	NHP2-like protein 1 homolog OS=Caenorhabditis elegans GN=M28.5 PE=3 SV=1	14.0	7.7	1	13.8 (M:13.8)	1	9.4	4.39	220
221		true	COX5A_CAEEL	Cytochrome c oxidase subunit 5A, mitochondrial OS=Caenorhabditis elegans GN=cco-2 PE=1 SV=2	20.1	5.8	1	13.8 (M:13.8)	1	3.4	4.30	221
222		true	PHB2_CAEEL	Mitochondrial prohibitin complex protein 2 OS=Caenorhabditis elegans GN=phb-2 PE=1 SV=2	32.6	9.7	1	13.6 (M:13.6)	1	2.7	1.08	222
223		true	MED12_CAEEL	Mediator of RNA polymerase II transcription subunit 12 OS=Caenorhabditis elegans GN=dpy-22 PE=1 SV=2	400.5	8.5	1	13.5 (M:13.5)	1	0.7	0.69	223
224		true	PFD2_CAEEL	Prefoldin subunit 2 OS=Caenorhabditis elegans GN=pdf-2 PE=3 SV=1	16.0	5.4	1	13.5 (M:13.5)	1	6.4	4.29	224

Supplementary Table 3. One-hundred eighty proteins are shared between 3-day-old and 15-day-old worms

Row	Accession	Name
1	VIT6_CAEEL	Vitellogenin-6 OS=Caenorhabditis elegans GN=vit-6 PE=1 SV=5
2	VIT2_CAEEL	Vitellogenin-2 OS=Caenorhabditis elegans GN=vit-2 PE=1 SV=5
3	VIT5_CAEEL	Vitellogenin-5 OS=Caenorhabditis elegans GN=vit-5 PE=2 SV=2
4	VIT3_CAEEL	Vitellogenin-3 OS=Caenorhabditis elegans GN=vit-3 PE=1 SV=1
5	VIT1_CAEEL	Vitellogenin-1 OS=Caenorhabditis elegans GN=vit-1 PE=1 SV=2
6	MYO4_CAEEL	Myosin-4 OS=Caenorhabditis elegans GN=unc-54 PE=1 SV=2
7	HSP7A_CAEEL	Heat shock 70 kDa protein A OS=Caenorhabditis elegans GN=hsp-1 PE=1 SV=2
8	EF1A_CAEEL	Elongation factor 1-alpha OS=Caenorhabditis elegans GN=eft-3 PE=3 SV=1
9	TBB2_CAEEL	Tubulin beta-2 chain OS=Caenorhabditis elegans GN=tbb-2 PE=3 SV=1
10	HSP90_CAEEL	Heat shock protein 90 OS=Caenorhabditis elegans GN=daf-21 PE=1 SV=1
11	TBA2_CAEEL	Tubulin alpha-2 chain OS=Caenorhabditis elegans GN=tba-2 PE=2 SV=1
12	EF2_CAEEL	Elongation factor 2 OS=Caenorhabditis elegans GN=eef-2 PE=1 SV=4
13	CH60_CAEEL	Chaperonin homolog Hsp-60, mitochondrial OS=Caenorhabditis elegans GN=hsp-60 PE=1 SV=2
14	14331_CAEEL	14-3-3-like protein 1 OS=Caenorhabditis elegans GN=par-5 PE=1 SV=2
15	U375A_CAEEL	UPF0375 protein C08F11.11 OS=Caenorhabditis elegans GN=C08F11.11 PE=1 SV=1
16	TCTP_CAEEL	Translationally-controlled tumor protein homolog OS=Caenorhabditis elegans GN=tct-1 PE=1 SV=1
17	ALF2_CAEEL	Fructose-bisphosphate aldolase 2 OS=Caenorhabditis elegans GN=aldo-2 PE=2 SV=1
18	PPN1_CAEEL	Papilin OS=Caenorhabditis elegans GN=mig-6 PE=1 SV=1
19	HSP7D_CAEEL	Heat shock 70 kDa protein D OS=Caenorhabditis elegans GN=hsp-4 PE=1 SV=2
20	ATPA_CAEEL	ATP synthase subunit alpha, mitochondrial OS=Caenorhabditis elegans GN=H28O16.1 PE=1 SV=1
21	14332_CAEEL	14-3-3-like protein 2 OS=Caenorhabditis elegans GN=ftt-2 PE=1 SV=1
22	MYSP_CAEEL	Paramyosin OS=Caenorhabditis elegans GN=unc-15 PE=1 SV=1
23	SAHH_CAEEL	Adenosylhomocysteinase OS=Caenorhabditis elegans GN=ahcy-1 PE=1 SV=1
24	F37C4_CAEEL	Protein F37C4.5 OS=Caenorhabditis elegans GN=F37C4.5 PE=1 SV=3
25	ACON_CAEEL	Probable aconitate hydratase, mitochondrial OS=Caenorhabditis elegans GN=aco-2 PE=1 SV=2
26	PDI1_CAEEL	Protein disulfide-isomerase 1 OS=Caenorhabditis elegans GN=pdi-1 PE=3 SV=1
27	CYP7_CAEEL	Peptidyl-prolyl cis-trans isomerase 7 OS=Caenorhabditis elegans GN=cyn-7 PE=1 SV=2
28	GBLP_CAEEL	Guanine nucleotide-binding protein subunit beta-2-like 1 OS=Caenorhabditis elegans GN=rack-1 PE=1 SV=3
29	HSP7F_CAEEL	Heat shock 70 kDa protein F, mitochondrial OS=Caenorhabditis elegans GN=hsp-6 PE=1 SV=2
30	KARG1_CAEEL	Probable arginine kinase F46H5.3 OS=Caenorhabditis elegans GN=F46H5.3 PE=1 SV=2
31	IFB1_CAEEL	Intermediate filament protein ifb-1 OS=Caenorhabditis elegans GN=ifb-1 PE=1 SV=1
32	VDAC_CAEEL	Probable voltage-dependent anion-selective channel OS=Caenorhabditis elegans GN=vdac-1 PE=3 SV=2
33	RSSA_CAEEL	40S ribosomal protein SA OS=Caenorhabditis elegans GN=rps-0 PE=1 SV=3
34	FABP2_CAEEL	Fatty acid-binding protein homolog 2 OS=Caenorhabditis elegans GN=lbp-2 PE=1 SV=1
35	SIP1_CAEEL	Stress-induced protein 1 OS=Caenorhabditis elegans GN=sip-1 PE=1 SV=1
36	ATPB_CAEEL	ATP synthase subunit beta, mitochondrial OS=Caenorhabditis elegans GN=atp-2 PE=1 SV=2
37	YZ10_CAEEL	Uncharacterized protein F08B12.4 OS=Caenorhabditis elegans GN=F08B12.4 PE=4 SV=2
38	TTR2_CAEEL	Transthyretin-like protein 2 OS=Caenorhabditis elegans GN=ttr-2 PE=1 SV=1
39	ENO_CAEEL	Enolase OS=Caenorhabditis elegans GN=enol-1 PE=1 SV=3
40	SYDC_CAEEL	Aspartate--tRNA ligase, cytoplasmic OS=Caenorhabditis elegans GN=drs-1 PE=3 SV=1
41	HSP7C_CAEEL	Heat shock 70 kDa protein C OS=Caenorhabditis elegans GN=hsp-3 PE=1 SV=2
42	RL4_CAEEL	60S ribosomal protein L4 OS=Caenorhabditis elegans GN=rpl-4 PE=1 SV=3
43	RLA1_CAEEL	60S acidic ribosomal protein P1 OS=Caenorhabditis elegans GN=rla-1 PE=3 SV=2
44	FAR2_CAEEL	Fatty-acid and retinol-binding protein 2 OS=Caenorhabditis elegans GN=far-2 PE=1 SV=1
45	MDHM_CAEEL	Probable malate dehydrogenase, mitochondrial OS=Caenorhabditis elegans GN=mdh-2 PE=3 SV=1
46	LMN1_CAEEL	Lamin-1 OS=Caenorhabditis elegans GN=lmn-1 PE=1 SV=2
47	PDI2_CAEEL	Protein disulfide-isomerase 2 OS=Caenorhabditis elegans GN=pdi-2 PE=1 SV=1
48	RS8_CAEEL	40S ribosomal protein S8 OS=Caenorhabditis elegans GN=rps-8 PE=3 SV=1
49	IPYR_CAEEL	Probable inorganic pyrophosphatase 1 OS=Caenorhabditis elegans GN=pyp-1 PE=3 SV=4
50	RLA2_CAEEL	60S acidic ribosomal protein P2 OS=Caenorhabditis elegans GN=rpa-2 PE=3 SV=2
Row	Accession	Name
51	TTR16_CAEEL	Transthyretin-like protein 16 OS=Caenorhabditis elegans GN=ttr-16 PE=1 SV=2
52	UNC87_CAEEL	Protein unc-87 OS=Caenorhabditis elegans GN=unc-87 PE=1 SV=3

53	LEC1_CAEEL	32 kDa beta-galactoside-binding lectin OS=Caenorhabditis elegans GN=lec-1 PE=1 SV=1
54	FABP9_CAEEL	Fatty acid-binding protein homolog 9 OS=Caenorhabditis elegans GN=lbp-9 PE=3 SV=1
55	RS2_CAEEL	40S ribosomal protein S2 OS=Caenorhabditis elegans GN=rps-2 PE=3 SV=1
56	RLA0_CAEEL	60S acidic ribosomal protein P0 OS=Caenorhabditis elegans GN=rpa-0 PE=1 SV=3
57	RS3_CAEEL	40S ribosomal protein S3 OS=Caenorhabditis elegans GN=rps-3 PE=3 SV=1
58	RL7_CAEEL	60S ribosomal protein L7 OS=Caenorhabditis elegans GN=rpl-7 PE=3 SV=1
59	MYO2_CAEEL	Myosin-2 OS=Caenorhabditis elegans GN=myo-2 PE=1 SV=2
60	EF1B2_CAEEL	Probable elongation factor 1-beta/1-delta 2 OS=Caenorhabditis elegans GN=eef-1B.2 PE=1 SV=4
61	CALR_CAEEL	Calreticulin OS=Caenorhabditis elegans GN=crt-1 PE=1 SV=1
62	TPM1_CAEEL	Tropomyosin isoforms a/b/d/f OS=Caenorhabditis elegans GN=lev-11 PE=1 SV=1
63	MPCP_CAEEL	Phosphate carrier protein, mitochondrial OS=Caenorhabditis elegans GN=F01G4.6 PE=2 SV=1
64	BTF3_CAEEL	Transcription factor BTF3 homolog OS=Caenorhabditis elegans GN=icd-1 PE=1 SV=1
65	EF1B1_CAEEL	Probable elongation factor 1-beta/1-delta 1 OS=Caenorhabditis elegans GN=eef-1B.1 PE=1 SV=1
66	RS4_CAEEL	40S ribosomal protein S4 OS=Caenorhabditis elegans GN=rps-4 PE=1 SV=1
67	G3P2_CAEEL	Glyceraldehyde-3-phosphate dehydrogenase 2 OS=Caenorhabditis elegans GN=gpd-2 PE=3 SV=2
68	YSX1_CAEEL	Uncharacterized protein T28D9.1 OS=Caenorhabditis elegans GN=T28D9.1 PE=4 SV=2
69	DLDH_CAEEL	Dihydrolipoyl dehydrogenase, mitochondrial OS=Caenorhabditis elegans GN=dld-1 PE=3 SV=2
70	RL19_CAEEL	60S ribosomal protein L19 OS=Caenorhabditis elegans GN=rpl-19 PE=3 SV=1
71	RS3A_CAEEL	40S ribosomal protein S3a OS=Caenorhabditis elegans GN=rps-1 PE=3 SV=2
72	RS12_CAEEL	40S ribosomal protein S12 OS=Caenorhabditis elegans GN=rps-12 PE=1 SV=2
73	RS14_CAEEL	40S ribosomal protein S14 OS=Caenorhabditis elegans GN=rps-14 PE=3 SV=1
74	RS7_CAEEL	40S ribosomal protein S7 OS=Caenorhabditis elegans GN=rps-7 PE=3 SV=1
75	RL7A_CAEEL	60S ribosomal protein L7a OS=Caenorhabditis elegans GN=rpl-7A PE=1 SV=3
76	ETFA_CAEEL	Probable electron transfer flavoprotein subunit alpha, mitochondrial OS=Caenorhabditis elegans GN=F27D4.1 PE=3 SV=2
77	YLA4_CAEEL	Uncharacterized protein C30C11.4 OS=Caenorhabditis elegans GN=C30C11.4 PE=1 SV=1
78	RL5_CAEEL	60S ribosomal protein L5 OS=Caenorhabditis elegans GN=rpl-5 PE=3 SV=1
79	CISY_CAEEL	Probable citrate synthase, mitochondrial OS=Caenorhabditis elegans GN=cts-1 PE=1 SV=1
80	IFA2_CAEEL	Intermediate filament protein ifa-2 OS=Caenorhabditis elegans GN=ifa-2 PE=1 SV=1
81	MLE_CAEEL	Myosin, essential light chain OS=Caenorhabditis elegans GN=mlc-3 PE=1 SV=1
82	SYV_CAEEL	Valine--tRNA ligase OS=Caenorhabditis elegans GN=vrs-2 PE=1 SV=1
83	TCPQ_CAEEL	T-complex protein 1 subunit theta OS=Caenorhabditis elegans GN=cct-8 PE=1 SV=3
84	EIF3A_CAEEL	Eukaryotic translation initiation factor 3 subunit A OS=Caenorhabditis elegans GN=egl-45 PE=3 SV=1
85	OAT_CAEEL	Probable ornithine aminotransferase, mitochondrial OS=Caenorhabditis elegans GN=C16A3.10 PE=3 SV=3
86	ASP6_CAEEL	Aspartic protease 6 OS=Caenorhabditis elegans GN=asp-6 PE=3 SV=1
87	RL6_CAEEL	60S ribosomal protein L6 OS=Caenorhabditis elegans GN=rpl-6 PE=1 SV=1
88	CYC21_CAEEL	Cytochrome c 2.1 OS=Caenorhabditis elegans GN=cyc-2.1 PE=1 SV=2
89	MYO3_CAEEL	Myosin-3 OS=Caenorhabditis elegans GN=myo-3 PE=2 SV=1
90	RS5_CAEEL	40S ribosomal protein S5 OS=Caenorhabditis elegans GN=rps-5 PE=3 SV=1
91	ADF1_CAEEL	Actin-depolymerizing factor 1, isoforms a/b OS=Caenorhabditis elegans GN=unc-60 PE=1 SV=2
92	RL8_CAEEL	60S ribosomal protein L8 OS=Caenorhabditis elegans GN=rpl-8 PE=3 SV=1
93	RL18_CAEEL	60S ribosomal protein L18 OS=Caenorhabditis elegans GN=rpl-18 PE=3 SV=1
94	RS6_CAEEL	40S ribosomal protein S6 OS=Caenorhabditis elegans GN=rps-6 PE=1 SV=1
95	CALM_CAEEL	Calmodulin OS=Caenorhabditis elegans GN=cmd-1 PE=1 SV=3
96	ATPD_CAEEL	ATP synthase subunit delta, mitochondrial OS=Caenorhabditis elegans GN=F58F12.1 PE=1 SV=1
97	CRI3_CAEEL	Conserved regulator of innate immunity protein 3 OS=Caenorhabditis elegans GN=cri-3 PE=1 SV=2
98	RL22_CAEEL	60S ribosomal protein L22 OS=Caenorhabditis elegans GN=rpl-22 PE=1 SV=3
99	FABP6_CAEEL	Fatty acid-binding protein homolog 6 OS=Caenorhabditis elegans GN=lbp-6 PE=1 SV=1
100	RL40_CAEEL	Ubiquitin-60S ribosomal protein L40 OS=Caenorhabditis elegans GN=ubq-2 PE=3 SV=2
Row	Accession	Name
101	FAR1_CAEEL	Fatty-acid and retinol-binding protein 1 OS=Caenorhabditis elegans GN=far-1 PE=3 SV=1
102	YZVL_CAEEL	Uncharacterized NOP5 family protein K07C5.4 OS=Caenorhabditis elegans GN=K07C5.4 PE=3 SV=1
103	CGH1_CAEEL	ATP-dependent RNA helicase cgh-1 OS=Caenorhabditis elegans GN=cgh-1 PE=1 SV=1
104	PSA1_CAEEL	Proteasome subunit alpha type-1 OS=Caenorhabditis elegans GN=pas-6 PE=1 SV=1
105	ACBP1_CAEEL	Acyl-CoA-binding protein homolog 1 OS=Caenorhabditis elegans GN=acbp-1 PE=3 SV=1
106	SYSC_CAEEL	Probable serine--tRNA ligase, cytoplasmic OS=Caenorhabditis elegans GN=srs-2 PE=3 SV=1
107	RL9_CAEEL	60S ribosomal protein L9 OS=Caenorhabditis elegans GN=rpl-9 PE=3 SV=1
108	RL10A_CAEEL	60S ribosomal protein L10a OS=Caenorhabditis elegans GN=rpl-10a PE=3 SV=1
109	RL12_CAEEL	60S ribosomal protein L12 OS=Caenorhabditis elegans GN=rpl-12 PE=3 SV=1

110	LMP1_CAEEL	LAMP family protein lmp-1 OS=Caenorhabditis elegans GN=lmp-1 PE=2 SV=1
111	DIM_CAEEL	Disorganized muscle protein 1 OS=Caenorhabditis elegans GN=dim-1 PE=1 SV=3
112	VATA_CAEEL	V-type proton ATPase catalytic subunit A OS=Caenorhabditis elegans GN=vha-13 PE=1 SV=3
113	RS28_CAEEL	40S ribosomal protein S28 OS=Caenorhabditis elegans GN=rps-28 PE=3 SV=1
114	RL24_CAEEL	60S ribosomal protein L24 OS=Caenorhabditis elegans GN=rpl-24.1 PE=3 SV=1
115	PGK_CAEEL	Probable phosphoglycerate kinase OS=Caenorhabditis elegans GN=pgk-1 PE=1 SV=1
116	RS25_CAEEL	40S ribosomal protein S25 OS=Caenorhabditis elegans GN=rps-25 PE=3 SV=1
117	ODP2_CAEEL	Dihydrolipoyllysine-residue acetyltransferase component of pyruvate dehydrogenase complex, mitochondrial OS=Caenorhabditis elegans GN=F23B12.5 PE=1 SV=1
118	PDIA6_CAEEL	Probable protein disulfide-isomerase A6 OS=Caenorhabditis elegans GN=tag-320 PE=3 SV=1
119	IFA1_CAEEL	Intermediate filament protein ifa-1 OS=Caenorhabditis elegans GN=ifa-1 PE=1 SV=2
120	TPIS_CAEEL	Triosephosphate isomerase OS=Caenorhabditis elegans GN=tpi-1 PE=1 SV=2
121	RL3_CAEEL	60S ribosomal protein L3 OS=Caenorhabditis elegans GN=rpl-3 PE=2 SV=1
122	EF1G_CAEEL	Probable elongation factor 1-gamma OS=Caenorhabditis elegans GN=eef-1G PE=3 SV=1
123	RHO1_CAEEL	Ras-like GTP-binding protein rhoA OS=Caenorhabditis elegans GN=rho-1 PE=1 SV=1
124	PSA7_CAEEL	Proteasome subunit alpha type-7 OS=Caenorhabditis elegans GN=pas-4 PE=1 SV=1
125	FBRL_CAEEL	rRNA 2'-O-methyltransferase fibrillarin OS=Caenorhabditis elegans GN=fib-1 PE=3 SV=1
126	PSA5_CAEEL	Proteasome subunit alpha type-5 OS=Caenorhabditis elegans GN=pas-5 PE=1 SV=1
127	RL18A_CAEEL	60S ribosomal protein L18a OS=Caenorhabditis elegans GN=rpl-20 PE=3 SV=2
128	SYRC_CAEEL	Probable arginine--tRNA ligase, cytoplasmic OS=Caenorhabditis elegans GN=rrt-1 PE=3 SV=2
129	VATH2_CAEEL	Probable V-type proton ATPase subunit H 2 OS=Caenorhabditis elegans GN=vha-15 PE=3 SV=1
130	COX6A_CAEEL	Probable cytochrome c oxidase subunit 6A, mitochondrial OS=Caenorhabditis elegans GN=tag-174 PE=3 SV=1
131	NACA_CAEEL	Nascent polypeptide-associated complex subunit alpha OS=Caenorhabditis elegans GN=Y65B4BR.5 PE=1 SV=1
132	RS17_CAEEL	40S ribosomal protein S17 OS=Caenorhabditis elegans GN=rps-17 PE=3 SV=2
133	UCR1_CAEEL	Cytochrome b-c1 complex subunit 1, mitochondrial OS=Caenorhabditis elegans GN=ucr-1 PE=3 SV=2
134	PHB2_CAEEL	Mitochondrial prohibitin complex protein 2 OS=Caenorhabditis elegans GN=phb-2 PE=1 SV=2
135	RAN_CAEEL	GTP-binding nuclear protein ran-1 OS=Caenorhabditis elegans GN=ran-1 PE=1 SV=1
136	RL11_CAEEL	60S ribosomal protein L11 OS=Caenorhabditis elegans GN=rpl-11.1 PE=3 SV=1
137	OLA1_CAEEL	Obg-like ATPase 1 OS=Caenorhabditis elegans GN=ola-1 PE=3 SV=1
138	ODPA_CAEEL	Probable pyruvate dehydrogenase E1 component subunit alpha, mitochondrial OS=Caenorhabditis elegans GN=T05H10.6 PE=3 SV=1
139	SODC_CAEEL	Superoxide dismutase [Cu-Zn] OS=Caenorhabditis elegans GN=sod-1 PE=1 SV=2
140	RL26_CAEEL	60S ribosomal protein L26 OS=Caenorhabditis elegans GN=rpl-26 PE=3 SV=1
141	TNNT_CAEEL	Troponin T OS=Caenorhabditis elegans GN=mup-2 PE=2 SV=1
142	STT3_CAEEL	Dolichyl-diphosphooligosaccharide--protein glycosyltransferase subunit STT3 OS=Caenorhabditis elegans GN=T12A2.2 PE=1 SV=1
143	VATG_CAEEL	Probable V-type proton ATPase subunit G OS=Caenorhabditis elegans GN=vha-10 PE=3 SV=1
144	CCHL_CAEEL	Probable cytochrome c-type heme lyase OS=Caenorhabditis elegans GN=cchl-1 PE=3 SV=1
145	RS9_CAEEL	40S ribosomal protein S9 OS=Caenorhabditis elegans GN=rps-9 PE=3 SV=1
146	GLH1_CAEEL	ATP-dependent RNA helicase glh-1 OS=Caenorhabditis elegans GN=glh-1 PE=1 SV=3
147	STIP1_CAEEL	Stress-induced-phosphoprotein 1 OS=Caenorhabditis elegans GN=sti-1 PE=1 SV=1
148	TDX1_CAEEL	Probable peroxiredoxin prdx-3 OS=Caenorhabditis elegans GN=prdx-3 PE=1 SV=1
149	AT1B1_CAEEL	Sodium/potassium-transporting ATPase subunit beta-1 OS=Caenorhabditis elegans GN=nkb-1 PE=1 SV=1
150	SYTC_CAEEL	Threonine--tRNA ligase, cytoplasmic OS=Caenorhabditis elegans GN=trs-1 PE=3 SV=1
Row	Accession	Name
151	YQ83_CAEEL	GYF domain-containing protein C18H9.3 OS=Caenorhabditis elegans GN=C18H9.3/C18H9.2 PE=3 SV=3
152	RL35_CAEEL	60S ribosomal protein L35 OS=Caenorhabditis elegans GN=rpl-35 PE=3 SV=1
153	RL31_CAEEL	60S ribosomal protein L31 OS=Caenorhabditis elegans GN=rpl-31 PE=3 SV=1
154	DIG1_CAEEL	Mesocentin OS=Caenorhabditis elegans GN=dig-1 PE=1 SV=2
155	RL27_CAEEL	60S ribosomal protein L27 OS=Caenorhabditis elegans GN=rpl-27 PE=2 SV=1
156	TIM9_CAEEL	Mitochondrial import inner membrane translocase subunit Tim9 OS=Caenorhabditis elegans GN=tin-9.1 PE=3 SV=1
157	RL28_CAEEL	60S ribosomal protein L28 OS=Caenorhabditis elegans GN=rpl-28 PE=1 SV=3
158	RSP3_CAEEL	Probable splicing factor, arginine/serine-rich 3 OS=Caenorhabditis elegans GN=rsp-3 PE=1 SV=2
159	AL7A1_CAEEL	Putative aldehyde dehydrogenase family 7 member A1 homolog OS=Caenorhabditis elegans GN=alh-9 PE=3 SV=2
160	MMSA_CAEEL	Probable methylmalonate-semialdehyde dehydrogenase [acylating], mitochondrial OS=Caenorhabditis elegans GN=alh-8 PE=1 SV=1
161	RL23_CAEEL	60S ribosomal protein L23 OS=Caenorhabditis elegans GN=rpl-23 PE=3 SV=1
162	CSK2A_CAEEL	Casein kinase II subunit alpha OS=Caenorhabditis elegans GN=kin-3 PE=1 SV=1
163	LARP1_CAEEL	La-related protein 1 OS=Caenorhabditis elegans GN=larp-1 PE=3 SV=2
164	SYFB_CAEEL	Phenylalanine--tRNA ligase beta subunit OS=Caenorhabditis elegans GN=frs-2 PE=1 SV=2
165	EIF3C_CAEEL	Eukaryotic translation initiation factor 3 subunit C OS=Caenorhabditis elegans GN=eif-3.C PE=3 SV=2
166	LE767_CAEEL	Very-long-chain 3-oxooacyl-coA reductase let-767 OS=Caenorhabditis elegans GN=let-767 PE=1 SV=2

167	ALF1_CAEEL	Fructose-bisphosphate aldolase 1 OS=Caenorhabditis elegans GN=aldo-1 PE=1 SV=1
168	SYAC_CAEEL	Alanine--tRNA ligase, cytoplasmic OS=Caenorhabditis elegans GN=aars-2 PE=2 SV=1
169	TNNI2_CAEEL	Troponin I 2 OS=Caenorhabditis elegans GN=unc-27 PE=2 SV=2
170	METK1_CAEEL	Probable S-adenosylmethionine synthase 1 OS=Caenorhabditis elegans GN=sams-1 PE=1 SV=1
171	YOT9_CAEEL	Uncharacterized protein ZK632.9 OS=Caenorhabditis elegans GN=ZK632.9 PE=4 SV=3
172	RL44_CAEEL	60S ribosomal protein L44 OS=Caenorhabditis elegans GN=rpl-41 PE=3 SV=2
173	IF4E3_CAEEL	Eukaryotic translation initiation factor 4E-3 OS=Caenorhabditis elegans GN=ife-3 PE=1 SV=2
174	PGL1_CAEEL	P granule abnormality protein 1 OS=Caenorhabditis elegans GN=pgl-1 PE=1 SV=1
175	RL35A_CAEEL	60S ribosomal protein L35a OS=Caenorhabditis elegans GN=rpl-33 PE=1 SV=3
176	KC1A_CAEEL	Casein kinase I isoform alpha OS=Caenorhabditis elegans GN=kin-19 PE=3 SV=1
177	ATIF2_CAEEL	ATPase inhibitor mai-2, mitochondrial OS=Caenorhabditis elegans GN=mai-2 PE=3 SV=1
178	CWC22_CAEEL	Pre-mRNA-splicing factor CWC22 homolog OS=Caenorhabditis elegans GN=let-858 PE=2 SV=1
179	UNC22_CAEEL	Twitchin OS=Caenorhabditis elegans GN=unc-22 PE=1 SV=3
180	PRS10_CAEEL	Probable 26S protease regulatory subunit 10B OS=Caenorhabditis elegans GN=rpt-4 PE=1 SV=2

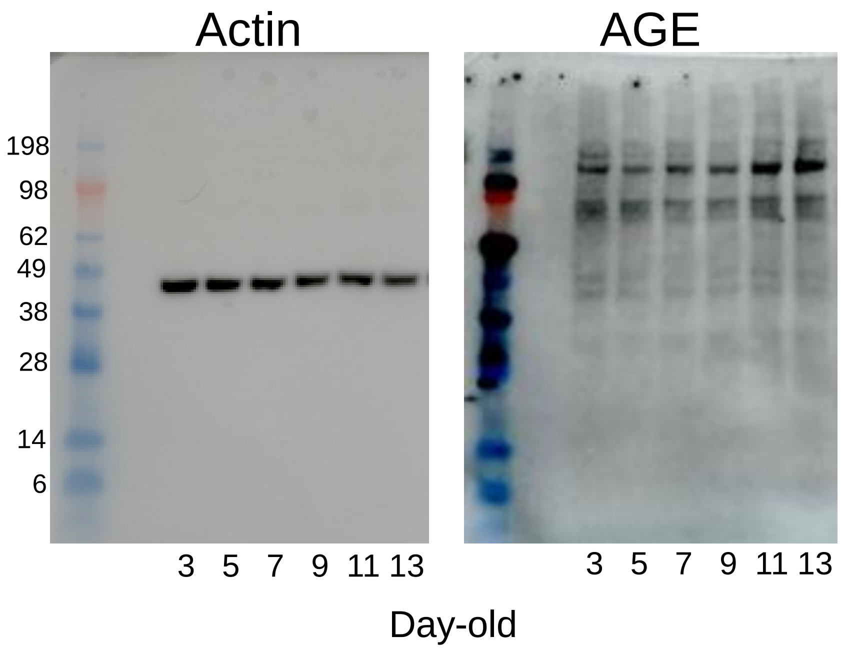

Supplementary Figure 1. Western blotting analysis of the AGE CML in samples extracted from worm (wild-type N2) populations of different ages. A representative photograph of three reproducible experiments is shown. Blots of Fig. 1c were cropped from this original photograph. A few size marker bands reacted with anti-CML antibody; this blot suggests that the proteinaceous markers also had been glycated.

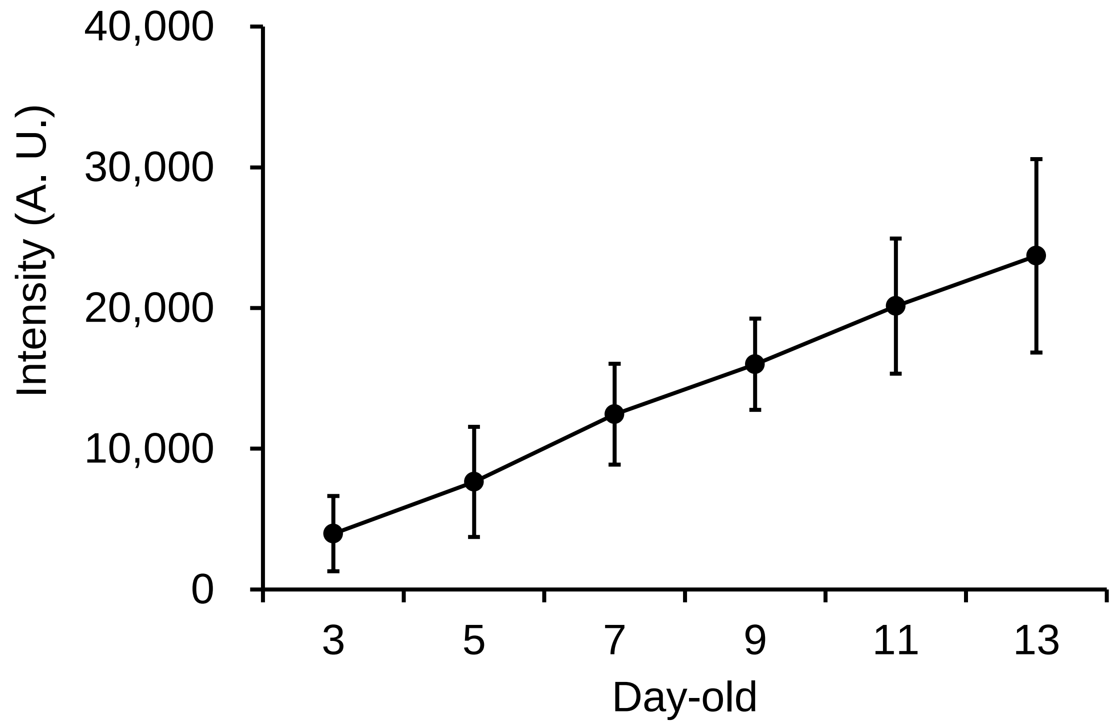

Supplementary Figure 2. Fluorescence spectrophotometry of individual worms measured by the non-invasive wrap-drop method. The data from Fig. 3b were transformed into a line showing means $\pm$ SD at each age.

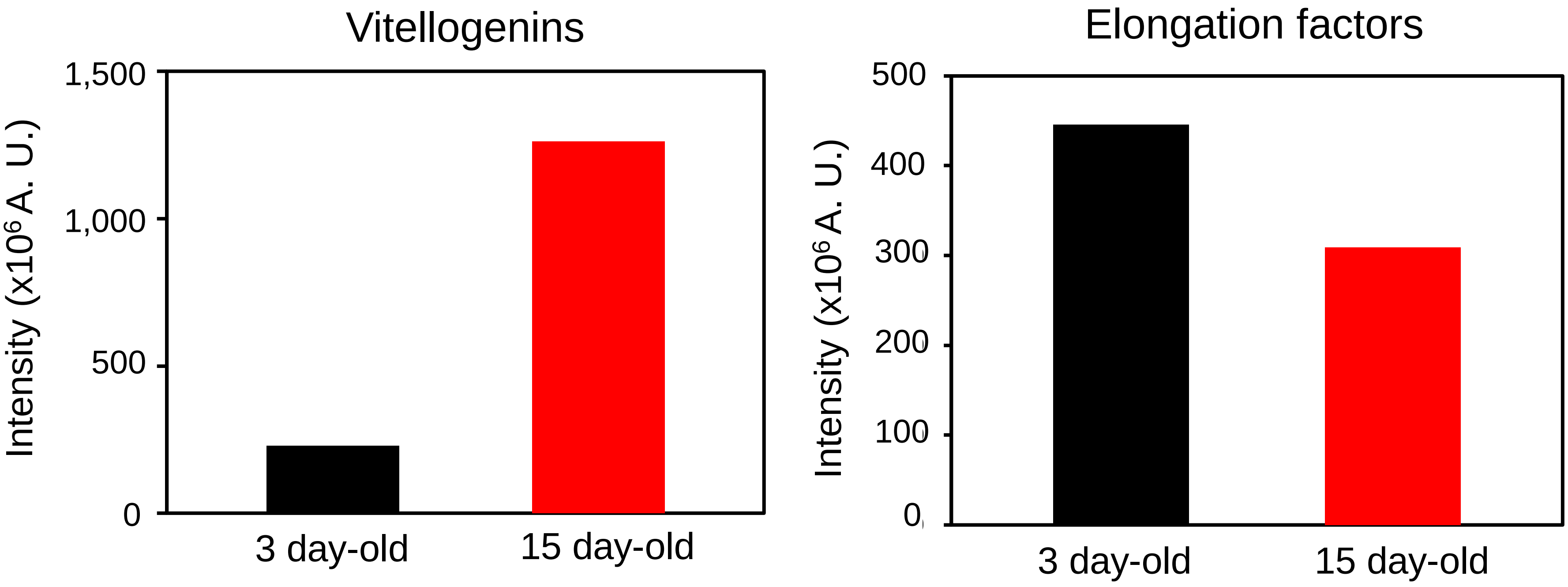

Supplementary Figure 3. Bars indicate the total amounts of fragment peaks corresponding to the vitellogenins 1 ~ 6 and elongation factors 1A, 1B1, 1G, and 2.
